# Results of a phase 1, randomized, placebo-controlled first-in-human trial of griffithsin formulated in a carrageenan vaginal gel

**DOI:** 10.1371/journal.pone.0261775

**Published:** 2022-01-20

**Authors:** Natalia Teleshova, Marla J. Keller, José A. Fernández Romero, Barbara A. Friedland, George W. Creasy, Marlena G. Plagianos, Laurie Ray, Patrick Barnable, Larisa Kizima, Aixa Rodriguez, Nadjet Cornejal, Claudia Melo, Gearoff Cruz Rodriguez, Sampurna Mukhopadhyay, Giulia Calenda, Shweta U. Sinkar, Thierry Bonnaire, Asa Wesenberg, Shimin Zhang, Kyle Kleinbeck, Kenneth Palmer, Mohcine Alami, Barry R. O’Keefe, Patrick Gillevet, Hong Hur, Yupu Liang, Gabriela Santone, Raina N. Fichorova, Tamara Kalir, Thomas M. Zydowsky

**Affiliations:** 1 Center for Biomedical Research, Population Council, New York, New York, United States of America; 2 Department of Medicine, Albert Einstein College of Medicine, Bronx, New York, United States of America; 3 Science Department, Borough of Manhattan Community College, New York, New York, United States of America; 4 University of Louisville, Louisville, Kentucky, United States of America; 5 Division of Cancer Treatment and Diagnosis, Molecular Targets Program, Center for Cancer Research and Natural Products Branch, Developmental Therapeutics Program, National Cancer Institute, Frederick, Maryland, United States of America; 6 George Mason University, Manassas, Virginia, United States of America; 7 Rockefeller University, New York, New York, United States of America; 8 Harvard Medical School, Boston, Massachusetts, United States of America; 9 Icahn School of Medicine at Mount Sinai, New York, New York, United States of America; University of Pittsburgh, UNITED STATES

## Abstract

HIV pre-exposure prophylaxis (PrEP) is dominated by clinical therapeutic antiretroviral (ARV) drugs. Griffithsin (GRFT) is a non-ARV lectin with potent anti-HIV activity. GRFT’s preclinical safety, lack of systemic absorption after vaginal administration in animal studies, and lack of cross-resistance with existing ARV drugs prompted its development for topical HIV PrEP. We investigated safety, pharmacokinetics (PK), pharmacodynamics (PD), and immunogenicity of PC-6500 (0.1% GRFT in a carrageenan (CG) gel) in healthy women after vaginal administration. This randomized, placebo-controlled, parallel group, double-blind first-in-human phase 1 study enrolled healthy, HIV-negative, non-pregnant women aged 24–45 years. In the open label period, all participants (n = 7) received single dose of PC-6500. In the randomized period, participants (n = 13) were instructed to self-administer 14 doses of PC-6500 or its matching CG placebo (PC-535) once daily for 14 days. The primary outcomes were safety and PK after single dose, and then after 14 days of dosing. Exploratory outcomes were GRFT concentrations in cervicovaginal fluids, PD, inflammatory mediators and gene expression in ectocervical biopsies. This trial is registered with ClinicalTrials.gov, number NCT02875119. No significant adverse events were recorded in clinical or laboratory results or histopathological evaluations in cervicovaginal mucosa, and no anti-drug (GRFT) antibodies were detected in serum. No cervicovaginal proinflammatory responses and no changes in the ectocervical transcriptome were evident. Decreased levels of proinflammatory chemokines (CXCL8, CCL5 and CCL20) were observed. GRFT was not detected in plasma. GRFT and GRFT/CG in cervicovaginal lavage samples inhibited HIV and HPV, respectively, *in vitro* in a dose-dependent fashion. These data suggest GRFT formulated in a CG gel is a safe and promising on-demand multipurpose prevention technology product that warrants further investigation.

## Introduction

HIV continues to be a major global public health issue, ranking as the leading cause of death in women aged 15–44 worldwide. In 2019, an estimated 38 million people globally were living with HIV [1]. Young women aged 15–24 are especially at risk, with approximately 5500 new infections each week worldwide [1]. In sub-Saharan Africa, five in six new infections among adolescents aged 15–19 years are among girls, and young women aged 15–24 years are twice as likely to be living with HIV than men of the same age [1].

Currently, once daily oral Truvada (tenofovir disoproxil fumarate-emtricitabine [TDF/FTC]) is the only product for HIV pre-exposure prophylaxis (PrEP) approved by the Food and Drug Administration (FDA) for cisgender women. Approval was based on the results of clinical trials demonstrating significantly reduced risk of HIV acquisition in cisgender men and transgender women who have sex with men (iPrEX) and in serodiscordant couples (Partners PrEP Study) [2, 3]. Truvada was shown to be efficacious in cisgender men and transgender women who have sex with men even if taken intermittently (STRAND) [4] or on-demand (IPERGAY) [5]. In contrast, daily Truvada did not demonstrate efficacy in two studies in cisgender women (FEM-PrEP and VOICE) [6, 7]. Low adherence rates [6, 7] potentially explain these results. Recent clinical trials (HPTN083 and HPTN 084) have demonstrated high efficacy of long-acting (LA) injectable Cabotegravir against HIV acquisition which was superior to Truvada [8, 9]. LA parenteral products (injectables and implants) could offer simpler administration regimens and may be preferred over daily oral regimens for some women. However, these products require health care provider delivery, have injection site and potential systemic side effects, and, for the case of implants, may not be easily removable. Alternatively, user-controlled vaginally administered PrEP (gel, fast dissolving vaginal insert (FDI), intravaginal rings (IVR), vaginal film) offers the distinct advantage of increasing local exposure to the active pharmaceutical ingredients (APIs) at the HIV transmission site and low to no systemic side effects.

Studies in the US and Africa demonstrated variability in preferences for HIV prevention products across groups and geographies and emphasized that women need options of different routes of PrEP administration and formulations suitable for their lives [10–12]. Expanding available PrEP options is likely to improve uptake of HIV prevention products just as contraceptive choice increased uptake, retention and contraceptive effectiveness (reviewed in [13, 14]). Therefore, it is critical to develop diverse PrEP products that align with preferences of people at risk of HIV infection.

In addition to adherence, other factors such as drug and dosage form-related characteristics (tenofovir distribution in colorectal tissues and female genital tissues [15]) and genital inflammation promoting susceptibility to HIV [16] may impact PrEP efficacy. Therefore, a thorough analysis of relationships between PrEP and mucosal environment (including transcriptome and proteome) may provide important data early in the product development process to identify PrEP candidates with favorable pharmacokinetics (PK) profiles across the conditions that do not induce perturbations in the mucosa that could lead to enhanced susceptibility to HIV and decreased PrEP efficacy.

The PrEP development pipeline has been dominated by repurposing approved therapeutic antiretroviral (ARV) drugs [13], which are undoubtedly an important addition to the toolbox of HIV prevention methods for women. However, a product that does not contain ARVs (particularly those approved for HIV treatment) offers several potential advantages. First, a product without an ARV is less likely to lead to drug-resistant HIV that may compromise subsequent ARV treatment options. Second, because the risk of resistance is low, non-ARV-based products are unlikely to require labeling indicating the need for HIV testing before and during use (once safety has been demonstrated in individuals with HIV). Removing the need for frequent HIV testing would overcome one of the barriers cited by individuals who are reluctant to use PrEP or who initiate PrEP and then discontinue [17–19]. Third, a non-ARV-based PrEP is likely to be approved faster by regulatory authorities as an over-the-counter (OTC) product than one with ARVs.

The Population Council is developing griffithsin (GRFT) as topical PrEP for on-demand use or sustained delivery. GRFT is a non-ARV lectin that has an outstanding anti-HIV activity (50% effective concentration (EC_50_) of 0.13 nM, and 90% effective concentration (EC_90_) of 0.58 nM) [20]. Unlike the “first generation” or broad-spectrum microbicides that did not demonstrate efficacy [21–29], GRFT acts by binding to the viral gp120 glycoprotein and blocking virus entry [30]. GRFT prevents both cell-free and cell-associated HIV transmission and blocks virus-cell fusion [31]. GRFT also has activity against other highly prevalent and morbid sexually transmitted infections (STIs), such as herpes simplex virus type-2 (HSV-2) and human papillomavirus (HPV), with overlapping HIV epidemiology that increase the risk of HIV acquisition and represent a significant public health burden in their own right [32]. GRFT inhibits HSV-2 by targeting viral entry and cell-to-cell mediated transmission [33, 34]. GRFT’s moderate activity against HPV is mediated by promoting HPV receptor internalization [34]. A GRFT multipurpose prevention technology (MPT) product that protects against HIV, HSV and HPV would be a promising addition to products in development based on anti-HIV and anti-HSV monoclonal antibodies [35, 36].

This report describes the results of a Phase 1 trial of PC-6500 (0.1% GRFT in a CG gel) in healthy female volunteers conducted in the Bronx, NY. The aim of this trial (Population Council Protocol No. 728) was to gather first-in-human safety data and support further development of GRFT in various topical formulations. The primary objectives were (1) to evaluate the safety of PC-6500 gel used vaginally for a single dose, and then once daily for 14 consecutive days of dosing, and (2) to assess PK of GRFT in blood after a single dose, and then after 14 days of dosing. Exploratory safety and pharmacodynamics (PD) objectives included analysis of soluble immune mediators, cervical transcriptome and anti-HIV/HPV activity of cervicovaginal secretions.

## Material and methods

### Trial design

A 14-day randomized, placebo-controlled, parallel group, double-blind Phase 1 trial of PC-6500, sponsored by the Population Council (New York, NY), was conducted at the Albert Einstein College of Medicine (Einstein) in the Bronx, NY. The main (randomized) period of the trial was preceded by a single-dose, open label (OL) period among a different group of participants. The protocol was approved by the Institutional Review Boards of the Population Council and Einstein prior to initiation of data collection. The protocol was submitted to the US Food and Drug Administration as IND # 123512 and registered at Clinical Trials.gov., number NCT02875119. All participants provided written informed consent before undergoing any procedures. Participants and staff at the Council and Einstein were blinded to the product assignment for the randomized portion of the study, but not time point, through the end of data collection.

### Study population

Healthy, HIV-negative, nonpregnant women aged 18–49 who were using an effective contraceptive method other than condoms or a vaginal ring, and who agreed to remain sexually abstinent during the study were invited to participate. Potential participants were assigned a unique identification number and screened for eligibility based on medical history, physical examination, pelvic examination, Pap smear if indicated, blood (CBC and chemistry) and STI testing, including HIV, chlamydia, gonorrhea, and trichomonas. Women with a laboratory abnormality, STI or abnormal Pap test were ineligible. Women with symptomatic BV, vulvovaginal candidiasis, or urinary tract infection at screening could be rescreened following completion of treatment.

### Study products, randomization, and blinding

All participants (n = 7) in the OL period were to receive one single 4 g dose of PC-6500 administered vaginally by a study clinician.

In the randomized period, 20 participants were instructed to self-administer 14 doses of PC-6500 or its matching CG placebo (PC-535) once daily for 14 days; OL participants were not eligible for the randomized period to prevent participants using both PC-6500 and placebo, which could confound data interpretation. Day 1/Dose 1 was scheduled as soon as possible after menstruation ended to maximize the likelihood that all 14 days of dosing would occur during the non-bleeding days of the menstrual cycle. Participants administered five of the 14 doses in the clinic with a study staff member in the room, but with a curtain drawn for privacy. The other nine doses were to be self-administered at home at approximately the same time each day. Both gels (Table 1) were manufactured and packaged in identical individually wrapped, prefilled, single-use, metered dose applicators (HTI Plastics; Lincoln, NE) at the Population Council’s Good Manufacturing Practices facility (Center for Biomedical Research, New York, NY). PC-6500 was manufactured using recombinant plant-produced GRFT [20]. The two study gels (PC-6500 and PC-535) differed imperceptibly in appearance; both were translucent with a faint beige color.

**Table 1:** Composition of PC-6500 and PC-535 (placebo) gel.

Ingredients	Composition/Quantity (g)	Percent (%w/w)	Function
**PC-6500**			
Griffithsin Solution (GRFT content 18.9 mg/mL)	108.98^a^	5.45^b^	API
WFI Quality Sterile Filtered Water	1812.78	90.64	Solvent
Sodium Acetate Trihydrate	5.24	0.26	Buffering agent
Sodium chloride	7.00	0.35	Osmolality adjuster
Carrageenan	62.00	3.10	Gel base/ Vehicle
Methylparaben	4.00	0.20	Preservative
1N Hydrochloric Acid	q.s.	q.s.	pH Adjuster
1N Sodium Hydroxide	q.s	q.s.	pH Adjuster
**PC-535**			
WFI Quality Sterile Filtered Water	1919.76	95.99	Solvent
Sodium Acetate Trihydrate	5.24	0.26	Buffering agent
Sodium chloride	7.00	0.35	Osmolality adjuster
Carrageenan	64.00	3.20	Gel base/ Vehicle
Methylparaben	4.00	0.20	Preservative
1N Hydrochloric Acid	q.s.	q.s.	pH adjuster
1N Sodium Hydroxide	q.s	q.s.	pH adjuster

^a^ GRFT content = 2.06g.

^b^ GRFT weight % = 0.1%.

As needed, HCl and NaOH were used to bring the in-process pH into the 6.8 to 7.5 range.

A Population Council statistician not associated with the trial created the randomization scheme that pre-assigned participants to either PC-6500 or placebo gel in a ratio of 7:3. Half of the participants were assigned blood and cervicovaginal lavage (CVLs) sample collection at 4h post dose; half were assigned to the 8h time point. Having different two time points for CVL collection allowed us to explore the length of time product remains present and active in the vagina. Randomization occurred in blocks of ten. Study staff received a list of kit numbers and specimen time point randomization assignments for 20 participants. All investigators, study staff and participants were blinded to product assignments. The post-dose specimen time points (4h, 8h and 24h) were not blinded. Due to inclement weather, specimen collection for one participant in the PC-6500 group was done at 6h instead of 8h post first dose.

Due to slower than anticipated accrual, it was not feasible to enroll all 20 women within the time and budget available. Therefore, the protocol was amended so that starting with the tenth enrollee, participants were assigned a kit number and time point, on a case-by-case basis, to maximize the number of women assigned to product vs. placebo, and to maintain the balance in post-dose specimen collection time points.

### Outcome measures and analytical methods

The schedule of visits and procedures performed in the OL and randomized periods of the study are presented in Tables 2 and 3, respectively. Outcome measures and analytical methods are described in detail below. The schedule was designed to collect BL samples and 24h post last gel samples (randomized phase) in late luteal phase to minimize effects of the menstrual cycle on exploratory outcomes.

**Table 2:** Visits and assessments, open label period.

Visit/Study Day	Visit 1: Enrollment/Day 1	Visit 2: Day 2	Visit 3: Day 8
	SINGLE DOSE IN CLINIC	24H POST-DOSE	CLOSING
**SAFETY/EXPOSURE**			
Adverse events	Pre- and post-dose	24h post-dose	Final safety assessment
EKG	Pre-dose	24h post-dose	N/A
Vital signs	Pre- and post-dose	24h post-dose	Final safety assessment
Urinalysis	If indicated	If indicated	Final safety assessment
Physical exam	If indicated	If indicated	Final safety assessment
Pelvic exam	Pre-dose	If indicated	Final safety assessment
Clinical labs (hematology, chemistry, coagulation)	Pre- and post-dose	24h post-dose	Final safety assessment
Used applicator collected for DSA testing	Post-dose	N/A	N/A
**PK**			
Plasma blood draw	Pre-dose and 0.5,1,2,3,4,6,8,10,12h post-dose	24h post-dose	N/A
**EXPLORATORY**			
CVLs to measure API concentration and PD	Pre-dose	24h post-dose	N/A

N/A—not applicable.

API—active pharmaceutical ingredient.

CVLs—cervicovaginal lavage samples.

DSA—dye stain applicator.

EKG—electrocardiogram.

PD—pharmacodynamics.

PK- pharmacokinetics.

**Table 3:** Visits and assessments, randomized period.

Visit/Study Day	BASELINE	Day 1	Day 3	Day 8	Day 11	Day 14	Day 15	Day 21	Day 28
		DOSE 1	DOSE 3	DOSE 8	DOSE 11	DOSE 14		FINAL SAFETY	
Adverse events	Yes	Pre-/post-dose	Pre-/post-dose	Pre-/post-dose	Pre-/post-dose	Pre-/post-dose	Yes	Yes	Yes
Vital signs	Yes	Pre-/post-dose	Pre-/post-dose	Pre-/post-dose	Pre-/post-dose	Pre-/post-dose	Post-dose	Yes	Yes
Physical exam	If indicated	If indicated	If indicated	If indicated	If indicated	If indicated	If indicated	Yes	If indicated
Pelvic exam	Yes	Pre-dose	If indicated	Pre-dose	Pre-dose	If indicated	If indicated	Yes	N/A
Urinalysis	If indicated	If indicated	If indicated	If indicated	If indicated	If indicated	If indicated	Yes	If indicated
Vaginal/cervical biopsy	Yes	N/A	N/A	N/A	N/A	N/A	Yes	N/A	N/A
Clinical labs	If indicated	Pre-dose	Pre-dose	Pre-dose	Pre-dose	Pre-dose	N/A	Yes	If indicated
DSA testing	N/A	Post-dose	Post-dose	Post-dose	Post-dose	Post-dose	N/A	N/A	N/A
ADA (serum)	N/A	Yes	N/A	N/A	N/A	Yes	N/A	Yes	Yes
Plasma PK	N/A	Pre-dose; 4 or 8h post-dose	Single PK	Single PK	Single PK	Pre-dose; 0.5,1,2,4,6,8h post-dose	24h post- dose 14	N/A	N/A
API, PD, immune mediators (CVLs)	N/A	Pre-dose; 4 or 8h post-dose	N/A	N/A	N/A	N/A	24h post-dose 14	N/A	N/A
Cervical transcriptome (biopsy)	Yes	N/A	N/A	N/A	N/A	N/A	Yes	N/A	N/A

N/A—not applicable.

ADA- anti-drug antibodies.

API—active pharmaceutical ingredient.

CVLs—cervicovaginal lavage samples.

DSA—dye stain assay.

PD—pharmacodynamics.

PK- pharmacokinetics.

### Clinical safety and exposure

Safety was evaluated at each study visit (Tables 2 and 3). Safety endpoints included treatment emergent adverse events (TEAEs) and medical significance, in the investigator’s judgment, of abnormalities in physical exams, pelvic exams, cervical and vaginal biopsies, and clinical laboratory parameters once product had been administered. AEs were coded using the Medical Dictionary for Regulatory Activities (MedDRA, Version 21.0), and graded according to the Division of AIDS Table for Grading the Severity of Adult and Pediatric Adverse Events, Version 1.0 and the Female Genital Table [37] for Use in Microbicide Studies.

Safety data were summarized by treatment group (OL, randomized PC-6500, placebo). Exposure was calculated by participant based on the number of applicators inserted vaginally, as measured by dye stain assay (DSA) test (sensitivity 93%, specificity 100%) [38, 39].

### Rapid Stain Identification (RSID) test

Swabs and CVLs were tested for presence of semenogelin using the RSID test (Independent Forensics, Lombard, IL).

### Histopathology

The histopathological assessment was performed by board-certified gynecologic pathologist using inflammation scoring as detailed in S1 Table.

### PK and concentrations of GRFT in CVLs

Concentrations of GRFT in plasma and in CVLs collected using 10 ml of sterile normal saline were measured by a validated indirect sandwich ELISA. 96-well Immulon 2HB microplates (Thermo Scientific, Rockford, IL) were pre-coated with HIV-1_BaL_ gp-120 (NIH Reagent Program Cat#49610, Germantown, MD). After blocking, the samples were added to the plate in duplicate. A goat anti-GRFT detection antibody (Pacific Immunology, Ramona, CA) was added and the mixture, followed by a rabbit anti-goat-HRP secondary antibody (Southern Biotech, Birmingham, AL). Ultra-TMB substrate (Thermo Scientific) was added followed by sulfuric acid (Thermo Scientific). Plates were washed and read on the Emax microplate reader (Molecular Devices, Sunnyvale, CA) at 450 nm (570 nm for reference) using the SoftMax Pro GxP 5.4.6 software (Molecular Devices). The lower limits of quantification (LLOQs) were 10 ng/mL and 1.25 ng/mL for plasma and CVL, respectively.

### Cytokine and chemokine (CC/CK) concentrations in CVL supernatants before and after gel use

Interleukin (IL)-1β (lower limit of detection (LLD) 0.3pg/mL), IL1 RA (LLD 13.1 pg/mL), IL-6 (LLD 0.5 pg/mL), CCL5 (LLD 0.19 pg/mL), CXCL8 (LLD 0.4 pg/mL), CCL20 (LLD 70.4 pg/mL) were measured using an electrochemiluminescence (ECL) platform (Meso Scale Discovery (MSD), Gaithersburg, MD) [40]. Each ECL immunoassay was optimized to allow detection of each biomarker within the linearity concentration range in the clarified CVL samples. IL1RA was tested in duplicates at 50- and 100-fold dilutions and when values exceeded the precision range of the assay, at an additional 1000-fold dilution. Other mediators were tested at a 2-fold or a 4-fold dilution, and where applicable undiluted, to generate duplicate values within linearity. The serial dilution of CVL samples resulted in reproducible values within the linearity range of each assay proving by accepted immunoassay methodology [41, 42] no matrix interference due to potential presence of study gel or other CVL matrix components. In addition, to rule out interference with the presence of gels, serial dilutions of a CG-containing gel were prepared in saline and mixed with equal volumes of biomarker solutions of known concentrations. Full CC/CK recovery was achieved in the concentration range of CG detected in CVL [43].

### RNA sequencing

Total RNA was isolated from ectocervical biopsy tissues taken from participants in the randomized study at BL pre-dose and post-dose 14 (one biopsy at each time point) and frozen in RNA*later* (Ambion) following the manufacturer’s instructions (RNeasy Fibrous Tissue Mini Kit (Qiagen; Germantown, MD)). The quality and purity of the extracted RNA were measured by the Agilent Bioanalyzer (Agilent, Santa Clara, CA). RNA was labeled, sequenced at the Rockefeller University (RU) Genomics Center by using Illumina TruSeq technology (75bp, >30M coverage) and analyzed as we previously published [44]. A multidimensional scaling plot (MDS) plot showing expression of top 500 genes in two dimensions was prepared within edgeR (doi: 10.1093/bioinformatics/btp616). To visualize gene expression patterns before and after gel treatment, a heatmap was plotted on the log transformation of count per million (logCPM) through R heatmap package (Kolde R, Package ’pheatmap’ for R. Version 1.0.8 (2015). Specifically, the top 500 variant genes (based on *p* adj. value) were selected to represent the heatmap. The volcano plots of gene expression according to the fold change and false discovery rate (FDR) adjusted *p* values were prepared through in-house R code.

### ADAs in serum

ELISA plates were coated with 20 μg/mL of GRFT. Following blocking and washes, serum samples and NM479 standards were added to the plate. For the samples, collected human serum was diluted 1:50. For the standard curve, 2X concentrated NM479 (1000 ng/mL– 0.98 ng/mL) was diluted 1:1 in 25X diluted serum (Innovative Research), for a final serum dilution of 50X and final NM479 concentrations ranging from 500 ng/mL to 0.49 ng/mL. Then biotinylated KQ-GRFT was added to the plates. KQ-GRFT is a modified version of Q-GRFT that contains a lysine at the N-terminus of the protein. Then plates were washed followed by incubation with horseradish peroxidase conjugated with streptavidin (SA-HRP) (Pierce). ADAs were detected using TMB (SeraCare), and the reaction was stopped with addition of sulfuric acid. OD 450 nm was measured on a Biotek Synergy HT plate reader.

### Anti-HIV-1_ADA-M_ activity of CVLs in TZM-bl MAGI assay

The multinuclear activation of a galactosidase indicator (MAGI) assay in TZM-bl cells (NIH AIDS Reagent Program) [45] was modified for evaluation of activity of GRFT in CVLs. Briefly, TMZ-bl cells cultured in 96-well white-opaque flat bottom microplates (Thermo Scientific) received different dilutions of non-clarified CVLs. GRFT solutions (between 100 and 0.015 ng/mL) prepared by diluting CMB-BDS-0900-003 GRFT stock (17 mg/mL) with cell culture medium were used as a control in the antiviral assay. All wells (including virus controls but excluding cell controls) were immediately challenged with 100–200 HIV-1_ADA-M_ infectious particles and stained using the MAGI assay following incubation [45]. All CVL dilutions and controls were tested in triplicate.

### Anti-HPV activity in CVLs

The anti-HPV activity of selected CVL samples was tested in HeLa cells using the luciferase assay [46, 47]. Briefly, HeLa cells cultured overnight at 10^5^ cells/mL in 96-well clear flat bottom microplates (Thermo Scientific) were incubated with different dilutions of non-clarified CVLs in triplicate. A 3% carrageenan gel (Population Council, lot#160613B6500 placebo TZ) diluted to between 1000 and 0.15 ng/mL with cell culture medium was used as a control in the antiviral assay. All wells (including virus controls but excluding cell controls) were immediately challenged with HPV16 PsV (5x10^5^ copies per well) and stained using the Pierce Firefly Luciferase Glow Assay (Thermo Scientific) [46] following incubation. All CVL dilutions and controls were tested in triplicate.

### Anti-HIV-1_BaL_ activity in CVLs in ectocervical explants

Ectocervical explants (5x5mm) were prepared as we previously described [48], stimulated with PHA (5 μg/mL) and IL-2 (100 U/mL) and then challenged with 500 TCID_50_ HIV-1_BaL_/explant (in duplicate) in the presence of non-clarified CVLs. HIV-1_BaL_ was generated and titered as previously described [43]. Ten μL of viral stock diluted in CVL (500 TCID_50_ of HIV_BaL_; 1.79μl of virus and 8.21μl of CVL) was applied on the top of the epithelium for 2h at 37°C in 5% CO_2_ followed by a wash out. Tissues were then cultured in complete DMEM for 14 days (d). Where feasible, untreated control (culture medium) was included. Also, to discriminate between the viral inoculum and *de novo* viral replication lamivudine (3TC: 4μL of 500μM stock applied on the epithelium) control was included. Tissue culture supernatants were collected right after washes (d0) and during culture (d3, 7, 11, 14). Infection was monitored with validated HIV *gag* reverse transcription-quantitative PCR (qRT-PCR) with LLOQ 20,000 copies/mL. Any value below LLOQ was set to 200001/√2 = 1099.74. Endpoint “soft” (SOFT; maximal viral growth) and cumulative (CUM) (d3 to 14) analyses were performed [43, 49].

### Epithelial integrity and ectocervical tissue viability in vitro

We explored if exposure to PC-6500 induces tissue toxicity, which may interfere with testing of anti-HIV_BaL_ activity of CVLs. Effects of PC-6500 on epithelial integrity and ectocervical tissue viability were analyzed by Hematoxylin and Eosin (H&E) staining and the MTT assay [3-(4,5-dimethyl-2-thiazolyl)-2,5-diphenyl-2H-tetrazolium bromide] assay [50], respectively. Human ectocervical tissues without gross pathological changes were obtained from routine hysterectomies through the National Disease Research Interchange (NDRI; Philadelphia, P) and transported overnight (ON) in RPMI medium. 5x5mm and 3x3mm explants were prepared as we previously described for macaque and human cervicovaginal tissues [48, 50]. Undiluted gels, PC-6500 (batches 131004B6500TR and 141027A6500TZ) and PC-535 (batch 130918A525TR), were applied on the epithelial surface of 5x5m explants and left ON (polarized cultures). Alternatively, 3x3mm explants were immersed in 1:30 and 1:100 diluted gels ON. Following incubation, tissues were processed for H&E staining (polarized cultures) or the MTT assay (immersion cultures).

### Statistical analysis

As is typical for first-in-human studies, no formal sample size calculation was carried out for this study. A sample size of seven participants in the single dose OL portion of the study, and 20 participants in the randomized portion (14 active; 6 placebo) was deemed sufficient to evaluate the safety of PC-6500 and to assess the PK of GRFT. Number and percent of participants with TEAEs are summarized by treatment arm and relationship and toxicity. Descriptive statistics are presented by treatment arm for demographic and baseline characteristics and for clinical endpoints. Analysis was done with SAS Version 9.4.

Log-normal generalized linear mixed models were used to analyze changes in CC/CK concentrations in CVL adjusting for treatment type. Treatment (PC-6500 or placebo) was a fixed effect in the model and a random effect for each participant was included. Values below LLD were assumed LLD/10. Pairwise differences were calculated using Tukey-adjusted 95% confidence intervals (CIs).

For transcriptome analysis, edgeR* v 3.16.5 [51] was used to normalize the samples and Voom from limma# v 3.30.11 was applied to estimate the differential log fold change in the expression of genes. An FDR adjusted *p* value <0.05 was used to determine if there was a change in cervical gene expression post gel application.

For analysis of anti-HIV-1_ADA-M_ activity of CVLs in TZM-bl MAGI assay, the EC_50_ and 95% CIs were calculated using a curve-fitting analysis (GraphPad Prism 5.0 Software, Inc., La Jolla, CA). GRFT concentrations and anti-HIV activity in CVLs were correlated using the Spearman correlation analysis (GraphPad Prism). Similarly, for analysis of anti-HPV activity in CVLs, the EC_50_ and 95% CIs were calculated using a curve-fitting analysis (GraphPad Prism).

For analysis of anti-HIV-1_BaL_ activity of CVLs in ectocervical explants, log-normal generalized linear mixed models were used. The models examined the relationship between SOFT and CUM as the independent variables and treatment type, CVL collection time and their interaction as the dependent variables (fixed effects). A random intercept for each participant was included. Pairwise comparisons were calculated and stepdown simulation-adjusted 95% CIs reported. GRFT concentrations and anti-HIV activity in CVLs were correlated using the Spearman correlation analysis (GraphPad Prism).

For analysis of tissue viability after exposure to gels *in vitro*, log-normal generalized linear mixed models with random intercepts were used. Optical density (OD) 570/tissue weight (g) was the response variable. Predictors were gel treatments. Tissue donor was included as a random effect (SAS).

## Results

### Disposition and demographics

**Fig 1:**
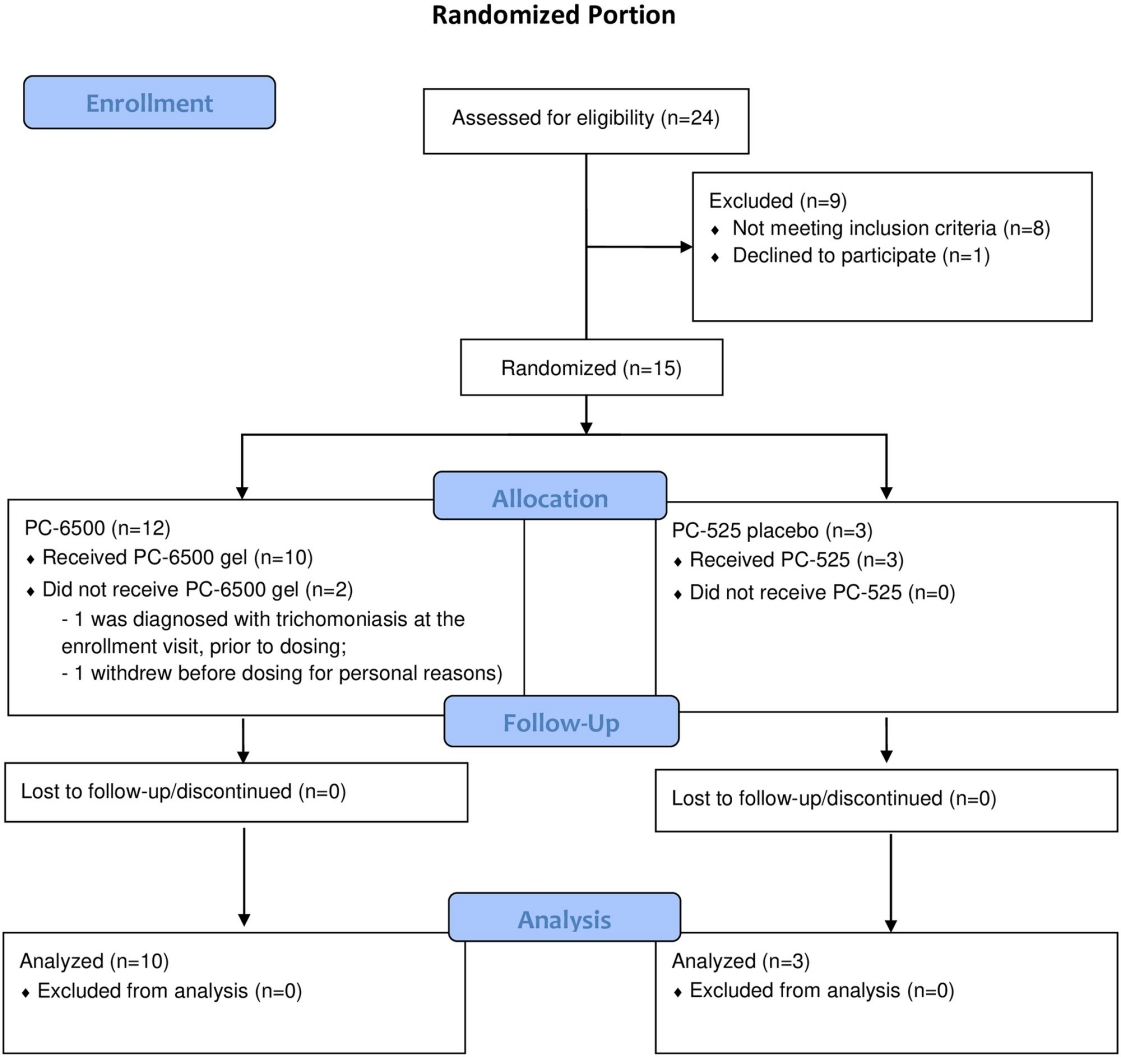
CONSORT flow diagram.

Seven participants were enrolled in the single dose OL period, all of whom completed the study (October-November 2017). As summarized in CONSORT Fig 1, 24 women were screened for the randomized portion of the study and 15 participants enrolled: 12 in the PC-6500 group and 3 in the placebo group. Thirteen participants (10/12 PC-6500; 3/3 placebo) completed the study (March-August 2018). Of the nine women who did not enroll, one chose to withdraw after starting the screening process and the other eight were ineligible due to underlying conditions (n = 6), including anemia, irregular bleeding, and trichomonas; inability to tolerate the pelvic exam (n = 1) and the investigator’s discretion (n = 2). Data collection ended based on the timeframe and resources available from the donor. Two participants in the PC-6500 arm withdrew prior to dosing, one due to a trichomonas infection detected at enrollment, and the other for personal reasons.

Participants in both periods of the study were approximately 32 years of age, on average, representing a mix of races, ethnicities and other background characteristics (see Table 4).

**Table 4:** Participant demographics (n = 20).

	Open Label PC-6500		Randomized PC-6500		Randomized Placebo		Overall	
	(n = 7)		(n = 10)		(n = 3)		(n = 20)	
**Age (Years)**								
Mean (SD)	31.9 (7.90)		31.9 (5.67)		33.3 (10.41)		32.1 (6.83)	
Range	25–43		24–40		25–45		24–45	
	**n**	**(%)**	**n**	**(%)**	**n**	**(%)**	**n**	**(%)**
**Ethnicity**								
Hispanic or Latina	1	14.3%	2	20.0%	0	0.0%	3	15.0%
Not Hispanic or Latina	6	85.7%	8	80.0%	3	10%	17	85.0%
**Race** ^a^								
Black or African American	2	28.6%	4	40.0%	2	66.7%	8	40.0%
White	4	57.1%	7	70.0%	1	33.3%	12	60.0%
Asian	0	0.0%	1	10.0%	0	0.0%	1	5.0%
Other	1	14.3%	0	0.0%	0	0.0%	1	5.0%
**Marital Status**								
Married/Cohabiting	4	57.1%	5	50.0%	1	33.3%	10	50.0%
Single	2	28.6%	5	50.0%	2	66.7%	9	45.0%
Divorced	1	14.3%	0	0.0%	0	0.0%	1	5.0%
**Level of Education**								
College Graduate	5	71.4%	8	80.0%	2	66.7%	15	75.0%
Some College	2	28.6%	2	20.0%	1	33.3%	5	25.0%

^a^ Participants could select more than one race; total may be greater than 100%.

### Exposure

**Fig 2:**
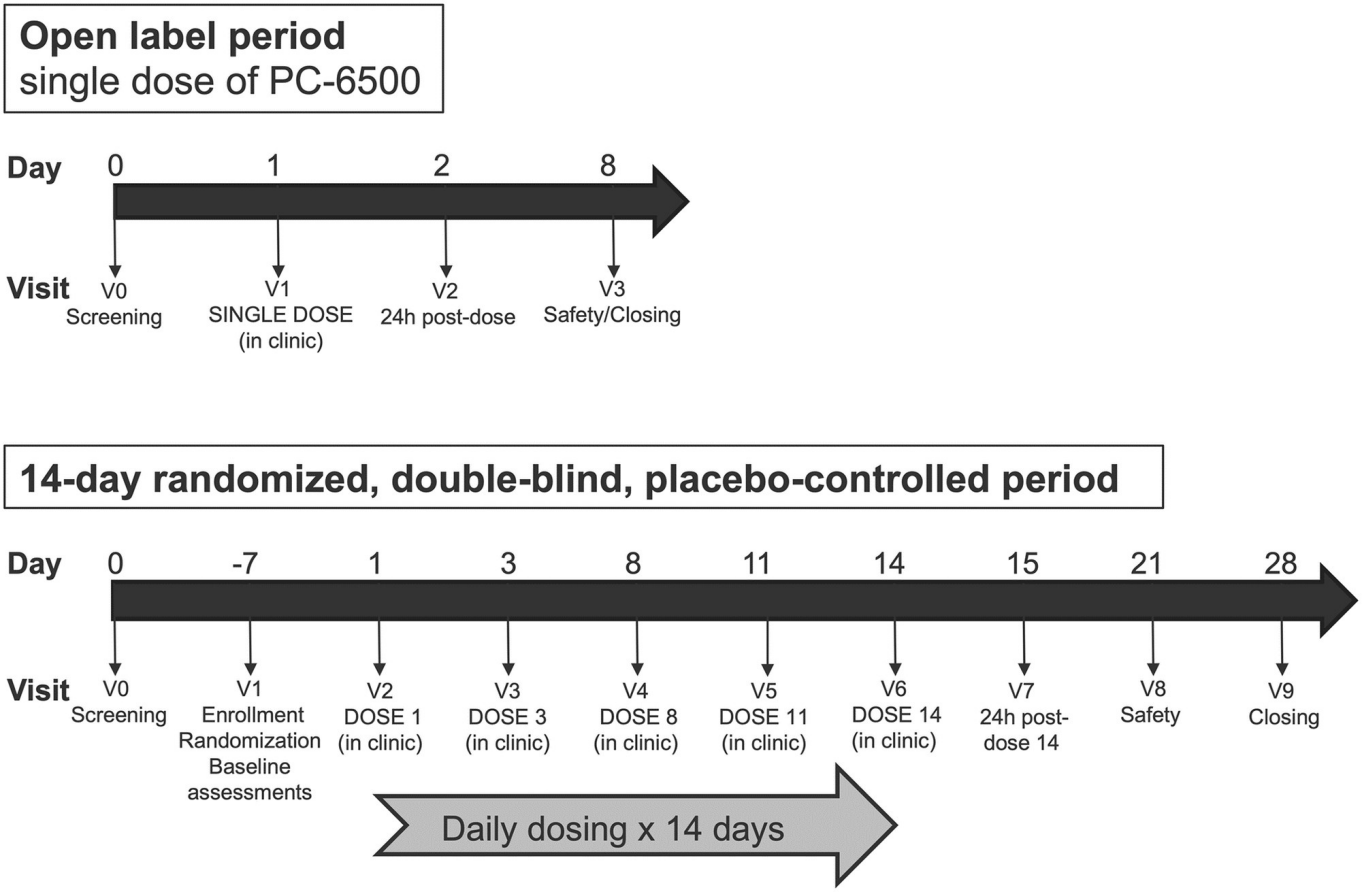
Clinical trial design.

Fig 2 illustrates the study design, including in-clinic and at-home dosing. All 7 participants in the OL period received one PC-6500 dose administered by the study clinician. The median dose exposure was 13 (range 3–14) in the randomized PC-6500 group and 14 (range 8–14) in the placebo group. Two of the three participants in the placebo group inserted all 14 doses per dye stain applicator (DSA) testing [38, 39]. In the randomized PC-6500 group, five of the ten participants inserted all 14 doses per DSA testing. Of the five participants in the randomized PC-6500 group who did not insert all applicators per the DSA, two inserted 13/14 doses, one inserted 12/14 doses, one inserted 8/14 doses and one appeared to have inserted only 1 out of 14 doses, including 4 of the 5 doses self-inserted in the clinic.

### Clinical safety

A total of 34 treatment emergent adverse events (TEAEs) were reported (Table 5) in five out of seven participants in the OL period and in 11 out of 13 participants (8 PC-6500, 3 placebo) in the randomized period. Only one event (vaginal discharge reported by a woman in the randomized PC-6500 group) was considered possibly related to study drug. The event was moderate in severity and resolved spontaneously without any intervention.

**Table 5:** Adverse event summary.

	Open Label PC-6500 (n = 7)	Randomized PC-6500 (n = 10)	Randomized Placebo (n = 3)	Overall (n = 20)
**Number of participants with any TEAE, n (%)**	5 (71.4%)	8 (80%)	3 (100%)	16 (80%)
With 1 TEAE	3 (42.9%)	3 (30%)	1 (33.3%)	7 (35%)
With 2 TEAEs	2 (28.6%)	1 (10%)	1 (33.3%)	4 (20%)
With 3 or more TEAEs	0 (0%)	4 (50%)	1 (33.3%)	5 (25%)
**Number of events**	**7**	**21**	**6**	**34**
**Relationship, n (%)**				
Not Related	7 (100.0%)	14 (66.7%)	4 (66.7%)	25 (73.5%)
Unlikely	-	6 (28.6%)	2 (33.3%)	8 (23.5%)
Possible	-	1 (4.8%)	-	1 (2.9%)
**Toxicity grade, n (%)**				
Grade 1	7 (100.0%)	17 (81.0%)	6 (100%)	30 (88.2%)
Grade 2	-	3 (14.3%)	-	3 (8.8%)
Grade 3	-	1 (4.8%) ^a^	-	1 (2.9%)
Vaginal discharge^b^	-	1 (4.8%)	-	1 (2.9%)

^a^AE with Grade 3 was for Activated partial thromboplastin time prolonged and marked as Grade 3 per DAIDS guidelines. The lab result was a probable lab error as the repeat test was within normal limits.

^b^Possibly related to the study product.

TEAE—treatment emergent adverse events.

Most adverse events (AEs) were transient and mild. Three TEAEs were judged to be of moderate severity: hyperkalemia, thrombocytopenia and hypophosphatemia, none of which were related to study drug or required additional interventions; these TEAEs occurred in 2 participants in the PC-6500 arm. TEAEs that required additional interventions were seasonal allergies (medication), and musculoskeletal pain (medication).

No serious AEs occurred. There were no clinically significant abnormalities found in physical or pelvic exams, and no clinically relevant shifts in any laboratory parameters.

### Histopathology

Ectocervical and vaginal biopsies were collected from 15 participants. All 15 participants had baseline (BL) biopsies and 13 had biopsies collected both at BL and one day after last gel application (10 PC-6500, 3 placebo) (n = 1 ectocervical and n = 1 vaginal). Evaluation of histologic data disclosed no findings of dysplasia or hyperplasia at BL or post gel administration. Most of the biopsies had mild to moderate inflammation (grades 1–2) in the epithelium/stroma at BL and post PC-6500 and placebo gel administration (S1 Table). In paired BL and post gel samples within PC-6500 group, the semiquantitative scores in cervical epithelium and stroma ranged from 0 to 2.5+ at BL and from 0 to 2 post gel. In vaginal epithelium and stroma the scores ranged from 0 to 1+ at BL and from 0 to 2 post gel. In placebo group, the scores in cervical epithelium and stroma ranged from 0 to 1 at BL and from 0.5 to 1+ post gel. In vaginal epithelium and stroma the scores ranged from 0 to 2 at BL and from 0 to 2+ post gel. No worsening of inflammation post gel administration was evident (S1 Table).

### GRFT PK in plasma

A total of 77 plasma samples from the OL period were collected, of which 23 were unevaluable due to coagulation resulting in a non-specific background. A total of 54 (71%) samples were analyzed. All samples from the randomized period were analyzed. GRFT levels in all samples were below LLOQ (10 ng/mL). As GRFT was undetectable after a single dose, or after multiple doses, no PK parameters were calculated.

### GRFT concentrations in CVLs

**Fig 3:**
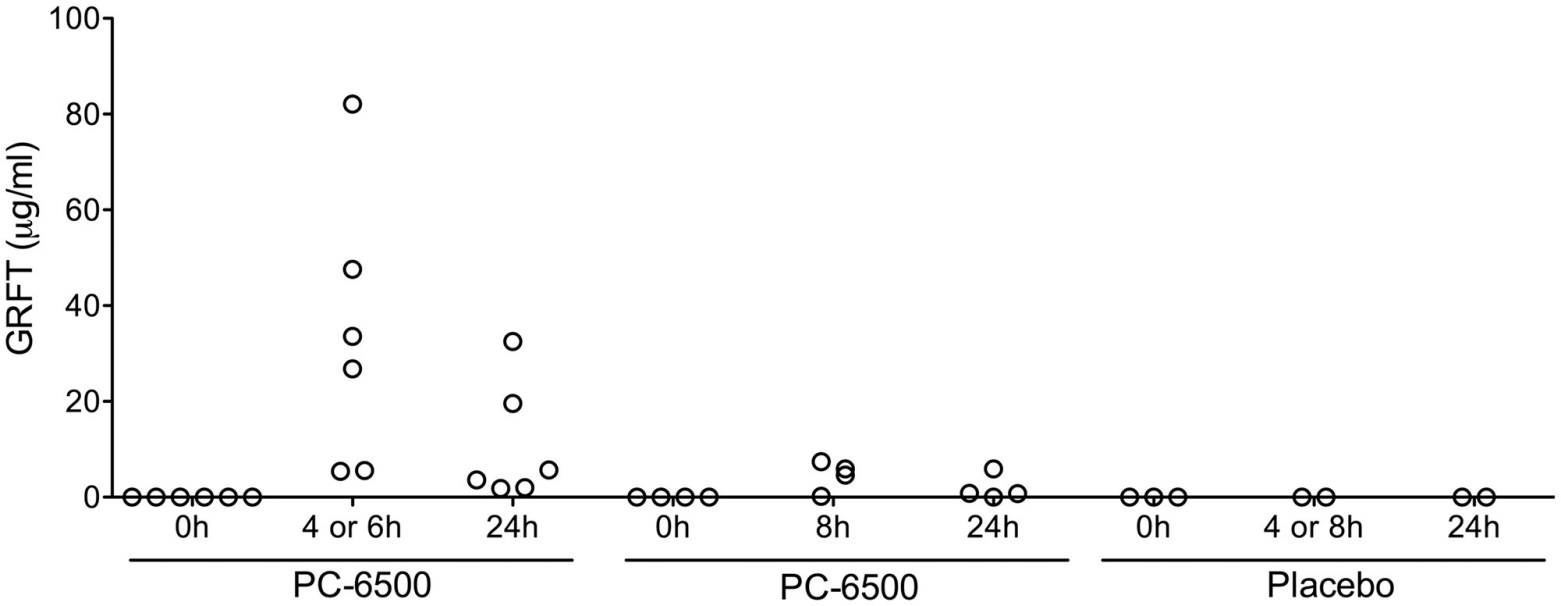
GRFT concentrations in CVL from participants in the randomized phase. GRFT concentrations 4h (or 6h) or 8h after single gel application and 24h after last gel application. 0h represents pre-gel dose BL. Each symbol represents an individual subject. Data below LLOQ are shown as 0. BL and 4h CVL samples from one subject in PC-6500 group were suspected to be switched at the time of collection. This has been adjusted for the presentation. Non-specific background was detected in 4h and 24h time points CVLs from a single subject in placebo group. These data were excluded.

Among participants randomized to PC-6500, CVLs collected at BL, 4h (or 6h) and 8h after single gel administration and 24h after dose 14 were evaluated. GRFT concentrations post gel administration ranged from between 0.0 and 82.1 μg/mL (Fig 3).

### CC/CK concentrations in CVLs

**Fig 4:**
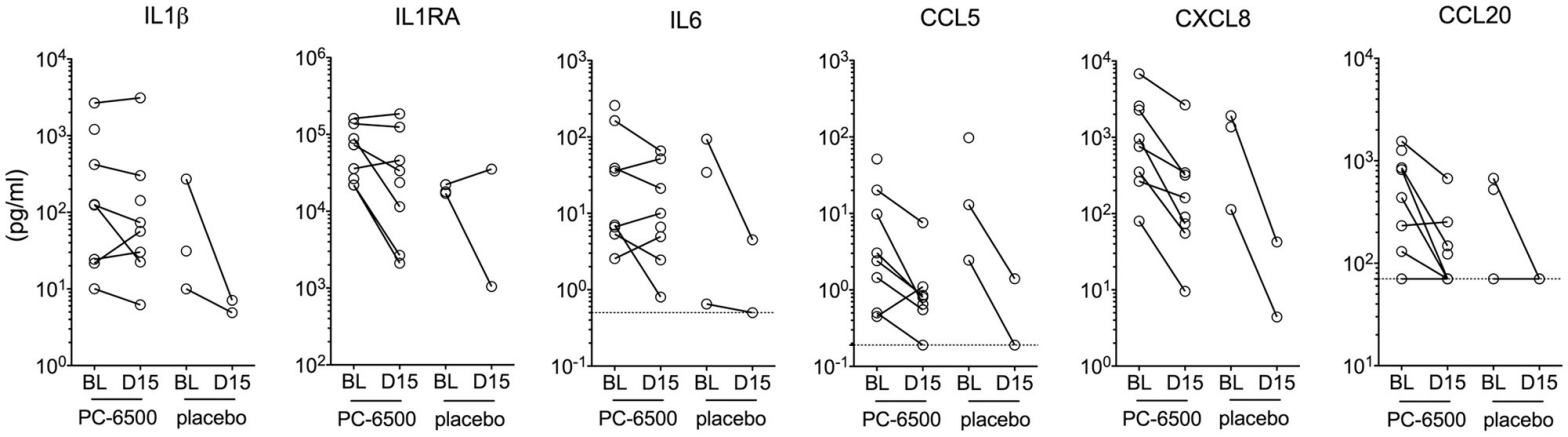
Concentrations of mediators of inflammation in CVLs. CVLs from participants in the randomized study (n = 10 PC-6500, n = 3 placebo) were collected pre-dose at BL and 24h post last gel application (D15) using saline. One subject (PC-6500 group) was excluded from the analysis as BL and 4h time-point CVLs were suspected to be switched at the time of collection. CVL samples with suspected blood (one post placebo gel, one post PC-6500 and one BL sample in PC-6500 group) were excluded from the analysis. Dotted lines represent lower limit of detection (LLD).

Biomarkers of inflammation were chosen for their established role in vaginal inflammation and HIV risk, reliable detection in cervicovaginal secretions, and clinically validated acceptance for microbicide safety evaluation [41, 52, 53]. CC/CK were measured in CVLs collected at BL and 24h post last gel administration in the randomized study (Fig 4). Concentrations of IL1β (least square mean ratio for post PC-6500 dosing: BL = 0.800, 95% CI [0.342,1.870], IL1RA (ratio = 0.364, 95% CI [0.119, 1.111]) and IL6 (ratio = 0.496, 95% CI [0.161, 1.529]) after repeated PC-6500 gel application were similar to BL. Decreased concentrations of CXCL8 after administration of both PC-6500 and placebo gels (ratio = 0.199, 95% CI [0.104, 0.380]; 0.032 [0.002, 0.466], respectively); decreased concentrations of CCL5 after administration of PC-6500 (ratio = 0.191, 95% CI [0.051, 0.720]) and decreased concentrations of CCL20 after administration of PC-6500 (ratio = 0.112, 95% CI [0.023, 0.546]) were detected. As RSID testing of CVLs and swabs did not detect semen in any samples, interference of immune mediators present in semen with the results was ruled out.

### Cervical tissue transcriptome

**Fig 5:**
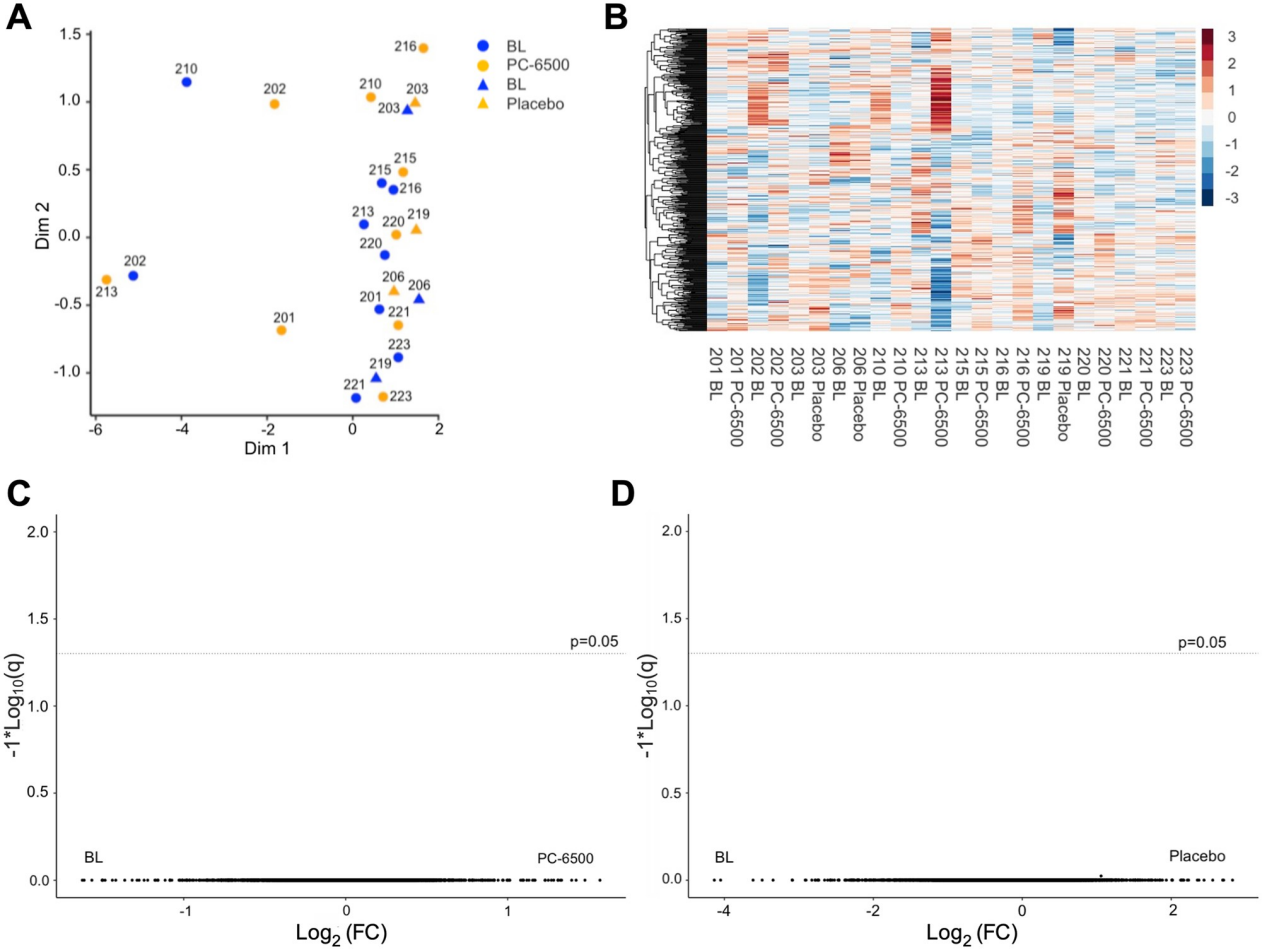
Ectocervical tissue transcriptome before and after repeated PC-6500 or placebo gel administration. Ectocervical gene expression in biopsies collected at BL and 24h after 14 days repeated gel administration is shown as **(A)** MDS plot, **(B)** heatmap and **(C, D)** volcano plots. MDS plot was prepared through plot MDS function within edgeR (doi: 10.1093/bioinformatics/btp616). The heatmap was constructed by edgeR using top 500 genes based on LogCPM. Induction (red) and inhibition (blue) of expression (z-scores) are shown. The volcano plots of gene expression at BL and post gel administration according to the fold change and adjusted *p* values (q < 0.05) were prepared through in-house R code. The y-axis corresponds to the mean expression value of log10 (FDR; q), and the x-axis displays the log2 fold change (FC) value. Samples from 9 subjects in PC-6500 group and from n = 3 subjects in placebo group were analyzed. Due to poor RNA quality, sample from one subject in PC-6500 group was excluded from the analysis.

Gene expression was analyzed in ectocervical biopsies collected at BL and 24h post last gel administration in the randomized period. An MDS plot revealed that most of the analyzed samples clustered together (Fig 5A), suggesting similar tissue gene expression before and after gel exposure. No specific patterns of gene expression before or after gel exposure were evident on the heatmap presentation (Fig 5B). Gene expression after repeated gel administration was similar to BL, as shown on the volcano plots (Fig 5C and 5D).

### ADAs

ADAs were evaluated in the randomized period of the study. No ADAs were detected at the tested time points (BL; d14, d21 and d28 post first gel application). Only one participant had a low positive optical density (OD) value of approximately 0.1 at BL. However, because we did not observe any increase in the later time points, we attributed the positive signal at BL to be an artifact or a pre-existing immune response to something that cross-reacts with GRFT.

### Pharmacodynamics

#### Anti-HIV-1_ADA-M_ and anti-HPV16 PsV activities in CVLs

**Fig 6:**
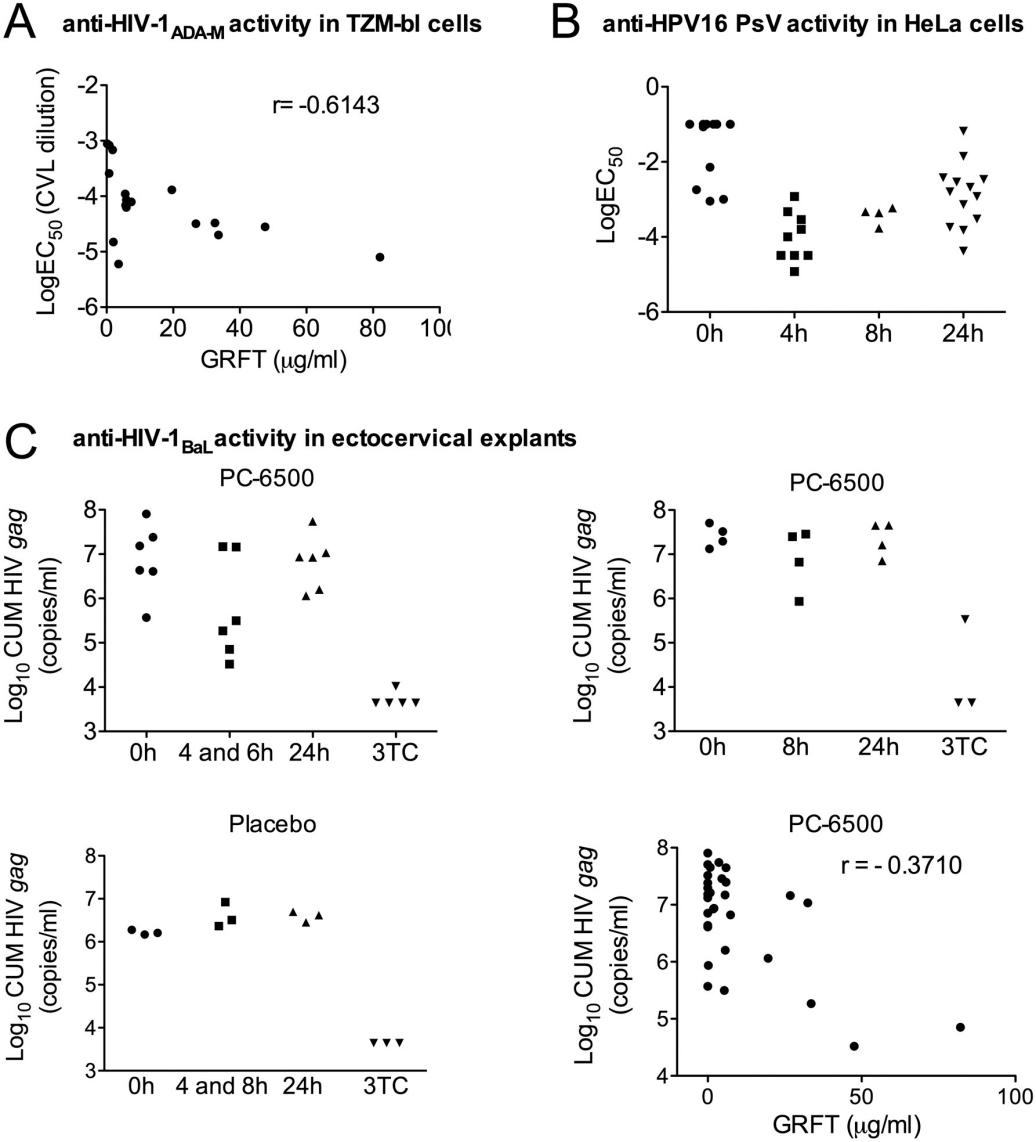
CVL activity against HIV-1 and HPV16 PsV. **(A)** TMZ-bl cells were incubated with cell-free HIV-1_ADA-M_ and different dilutions of CVLs collected at BL, 4 or 8h post single GRFT/CG gel administration and 24h after 14 day repeated gel administration (randomized study). The EC_50_ and 95% CI were calculated using a curve-fitting analysis with GraphPad Prism. The Spearman correlation analysis demonstrated that the higher the concentration of GRFT in CVL, the more potent the antiviral activity against HIV-1_ADA-M_ in the MAGI assay. **(B)** HeLa cells were incubated with HPV16 PsV and different dilutions of CVLs collected at BL, 4 or 8h post single PC-6500 gel administration and 24h post last gel administration (randomized study). The EC_50_ and 95% CI were calculated using a curve-fitting analysis with GraphPad Prism. **(C)** Polarized cervical explant cultures were challenged with 500 TCID_50_ HIV-1_BaL_ in the presence of CVLs collected at BL; 4, 6 or 8h post single and 24h after 14 day repeated gel administration (randomized study) applied on the epithelial surface for 2h (two explants per condition). Tissues were washed and cultured for 14d. Infection was monitored by HIV *gag* qRT-PCR using supernatants collected every 3-4d. 3TC (4μl of 500μM stock applied on the epithelium) control was included where feasible. The Spearman correlation analysis demonstrated that the higher the concentration of GRFT in CVLs, the more potent the antiviral activity against HIV-1_BaL_.

The antiviral activity of CVLs was tested in well-established TZM-bl and luciferase assays, as we previously published [45, 46]. EC_50_ values in the TZM-bl assay (0.7 ng/mL ± 0.6) based on GRFT concentrations suggest that CVL components did not affect the antiviral properties of GRFT. We were unable to estimate EC_50_ values in two CVLs where GRFT was quantified due to a sub-optimal curve fitting analysis (R square <0.8 and 95% CIs higher than 1log). As shown in Fig 6A, samples with high GRFT concentrations resulted in lower EC_50_ values based on CVL dilution; the more GRFT in the sample, the more dilution required to reach the EC_50_ value. Anti-HPV activity was evaluated in all CVLs from participants in the placebo and PC-6500 groups. CVLs recovered 4, 8 or 24h after gel application had EC_50_ values between 0.066 and 0.000032 (based on sample dilution). The mean EC_50_ values, based on CVL dilution factor, with the 95% CIs, were 0.071 [0.045; 0.097], 0.00026 [0.00004; 0.00055], 0.00042 [0.00013; 0.00069], 0.0074 [0.0034; 0.018] for CVLs collected at BL, 4, 8 and 24 hours after gel application, respectively. EC_50_ values from CVLs collected at all three time points post gel application were lower than BL EC_50_ values (Fig 6B). Lack of anti-HPV activity in CVL samples was noted for one participant in PC-6500 gel group, who appeared to have inserted only 1 of 14 doses according to the DSA.

#### Anti-HIV-1_BaL_ activity of CVLs in human ectocervical explants

Our previously published methodology for testing CVL activity in explants using an immersion tissue culture model [43] was adapted for use with polarized tissue cultures. PHA/IL2 activated explants were challenged with HIV-1_BaL_ in the presence of CVLs. Viral growth kinetics in individual experiments are shown in S1 Fig. SOFT and CUM analyses demonstrated a decrease in tissue HIV-1_BaL_ infection after exposure to CVLs collected 4 and 6h post PC-6500 gel application compared to placebo gel vs. 0h CVL (SOFT ratio = -4.27, 95% CI [-7.34, -1.20]; CUM ratio = -4.06, 95% CI [-6.96, -1.17]) (Fig 6C). No decrease in infection was observed after exposure to CVLs collected 8h post PC-6500 gel application compared to placebo gel vs. 0h (SOFT ratio = -1.10, 95% CI [-5.00, 2.80]; CUM ratio = -1.05, 95% CI [-4.73, 2.63]) or at 24h vs. 0h (SOFT ratio = -0.98, 95% CI [-3.64, 1.68]; CUM ratio = -1.01, 95% CI [-3.52, 1.50]) (Fig 6C). GRFT concentrations in CVLs directly correlated with HIV-1_BaL_ inhibition (Fig 6C). To rule out potential contribution of toxicity in these experiments, epithelial integrity and tissue viability post gel application were evaluated *in vitro*. No changes in epithelial integrity (e.g., epithelial fractures) or decreased viability were observed (S2A and S2B Fig).

## Discussion

In this first-in-human trial of 0.1% GRFT in a CG gel (PC-6500) there was no measurable systemic absorption, and no safety signals were detected in healthy, HIV-negative women using the gel for up to 14 consecutive daily doses. PC-6500 remained active against HIV and HPV in the presence of vaginal fluids *in vitro*. These results concur with preclinical assessments of GRFT and the GRFT/CG combination by the Population Council and others, demonstrating lack of systemic absorption, safety and activity against HIV and other STIs (HPV and HSV) [33, 34, 54].

No significant histopathological changes in cervical and vaginal mucosa and no ADA were detected. Consistent with this data, toxicology studies in small animal models following repeated vaginal administration of a GRFT/CG gel or intravenous (IV) administration of GRFT demonstrated no adverse events and showed that GRFT/CG gel is non-irritating. IV administration of GRFT in rats resulted in no detectable anti-drug antibodies (ADAs) [54].

Furthermore, rather than an increase in inflammatory CC/CK post gel application, a decrease in concentrations of CXCL8, CCL5 and CCL20 was detected post PC-6500 administration. Notably, CXCL8 and CCL20 are induced early during vaginal SIV infection in macaques followed by secondary inflammatory process likely driven by CCL5 and other CK-producing cells, leading to recruitment of immune cells and fueling infection [55]. Increased gene expression of CXCL8 and CCL20 in the cervix following exposure to Nonoxynol-9 (N9) gel is considered to be a “harm signal” [56]. CCL5 is involved in both the blocking of HIV binding to CCR5 on target cells and the recruitment of these target cells to the female genital tract [57–59]. The decreased concentrations of CXCL8, CCL5 and CCL20 post gel administration suggest a possible anti-inflammatory effect of PC-6500 gel.

Analysis of changes in gene expression may provide additional safety information in PrEP trials. PC-6500 or placebo gel did not induce changes in the ectocervical tissue transcriptome.

CVLs from women who used PC-6500 showed potent anti-HIV activity in cell-based and explant assays. In the polarized cervical explant model, which allows application of CVL and HIV on the epithelial surface mimicking the *in vivo* scenario, infection inhibition was detected after exposure to CVLs collected 4h or 6h post gel administration, but not 8h post gel. These data are consistent with *in vivo* data demonstrating significant protection of GRFT/CG FDI against vaginal simian human immunodeficiency virus (SHIV SF162P3) challenge [54]. DMPA-treated macaques were challenged with high dose vaginal SHIV SF162P3 4h after vaginal administration of either GRFT/CG FDIs (2 of 10 infected) or control CG FDIs containing all the same components except GRFT (10 of 10 infected). The activity of GRFT against SHIV SF162P3 infection in mucosal targets (in explants) was predictive of *in vivo* efficacy [54]. GRFT/CG FDIs were also effective against HSV-2 in mice [54]. Consistent with our published data on anti-HPV activity of CG and GRFT [34, 54], CVLs from participants in the PC-6500 and placebo groups inhibited HPV *in vitro*.

The study had several limitations. Although no sample size calculation was carried out, which is typical for first-in-human studies, the small sample size resulted in imbalance in baseline characteristics and may have been underpowered to detect some PD effects and may have limited the safety, PK and PD assessments. The potential limitations of the small sample size were compounded by imperfect adherence in half of the women in the PC-6500 group, which may have further limited the chances of detecting safety signals. In particular, the DSA results that indicated one participant inserted only a single dose of PC-6500 highlights the importance of emphasizing and assessing adherence even in early-stage clinical trials [60]. The study was not designed to assess intra-person heterogeneity or to formally monitor ADA response, which involves testing of samples over an extended period of time. Measurement of cervicovaginal fluid dilution in CVL was not feasible. Variation in the cervicovaginal fluid volume could have contributed to the variation in detected GRFT concentrations, CC/CK concentrations and could have affected PD results. Although we did not detect CG-mediated anti-HIV activity in CVLs derived from subjects administered placebo gel, we cannot exclude contribution of CG to the observed infection inhibition [43, 61]. Given modest anti-HPV activity of GRFT, it is likely that observed anti-HPV activity in the CVLs was mediated by CG [34].

In conclusion, PC-6500 represents a promising on-demand MPT product. PC-6500 containing potent non-ARV lectin GRFT may be preferred for individuals at high risk of HIV acquisition and who do not know their HIV status, decreasing the risk of developing drug resistance. Due to the anti-HPV and anti-HSV-2 activity of GRFT and CG, PC-6500 may also decrease HPV infection and HSV-2 infection/shedding, bolstering the anti-HIV activity of this prevention product. Lack of significant adverse events in clinical, laboratory or histopathological evaluations points to the safety of this MPT product.

Overall, a GRFT-containing on-demand or sustained-release product would fill an important gap in the current ARV-dominated prevention product pipeline. Data from this trial support the further clinical testing of GRFT/CG formulations for informing doses of GRFT for future trials and other GRFT delivery systems.

## Supporting information

S1 Fig HIV-1_BaL_ infection kinetics in ectocervical tissues. PHA/IL-2 activated polarized ectocervical explants were challenged with HIV-1_BaL_ in the presence of CVLs collected at **(A)** BL (0h CVL), 4h post first gel dose (4h CVL) and 24h post last dose (24h CVL); **(B)** 0h, 6h post first gel dose (6h CVL) and 24h post last dose; **(C)** 0h, 8h post first gel dose (8h CVL) and 24h post last dose. Controls included tissues challenged in the presence of medium (Control) or 3TC (diluted in medium). All CVLs collected from an individual subject were tested using ectocervical tissues from a single tissue donor. Each graph represents results using CVLs from an individual subject. Shown are MEAN±SEM HIV *gag* copies/mL of two explants per condition. One subject in placebo group had single explants included in control and 3TC conditions. BL and 4h CVL samples from one subject in PC-6500 group were suspected to be switched at the time of collection. This has been adjusted for the presentation and statistical analysis.
(TIF)

S2 Fig PC-6500 is not toxic to human ectocervical mucosa. **(A)** Polarized human ectocervical explants were cultured for ~18h in the presence of neat gels vs. medium applied on the epithelium (single explant/condition). To assess epithelial integrity after exposure to the gels, tissues were washed, paraffin-embedded, and stained with H&E. Representative of at least 3 experiments is shown. **(B)** Alternatively, tissues were immersed in medium containing diluted PC-6500 (vs. medium, diluted placebo and Gynol controls) (n = 2–3 explants/condition). Tissue viability was determined using MTT assay (OD_570_ of the formazan product was normalized to the dry weight of the explants). Each symbol indicates an individual donor and the Mean±SEM of the Log_10_ OD_570_/g of tissue for each condition is shown.
(TIF)

S1 File (PDF)

S1 Checklist (DOC)

S1 Table Histopathology. (DOCX)

## References

[1] http://www.unaids.org/en/resources/fact-sheet

[2] Grant RM, Lama JR, Anderson PL, McMahan V, Liu AY, Vargas L, et al. Preexposure chemoprophylaxis for HIV prevention in men who have sex with men. N Engl J Med. 2010;363(27):2587–99. doi: 10.1056/NEJMoa1011205 21091279 PMC3079639

[3] Baeten JM, Donnell D, Ndase P, Mugo NR, Campbell JD, Wangisi J, et al. Antiretroviral prophylaxis for HIV prevention in heterosexual men and women. N Engl J Med. 2012;367(5):399–410. doi: 10.1056/NEJMoa1108524 22784037 PMC3770474

[4] Anderson PL, Glidden DV, Liu A, Buchbinder S, Lama JR, Guanira JV, et al. Emtricitabine-tenofovir concentrations and pre-exposure prophylaxis efficacy in men who have sex with men. Sci Transl Med. 2012;4(151):151ra25. doi: 10.1126/scitranslmed.3004006 22972843 PMC3721979

[5] Molina JM, Charreau I, Spire B, Cotte L, Chas J, Capitant C, et al. Efficacy, safety, and effect on sexual behaviour of on-demand pre-exposure prophylaxis for HIV in men who have sex with men: an observational cohort study. Lancet HIV. 2017;4(9):e402–e10. doi: 10.1016/S2352-3018(17)30089-9 28747274

[6] Van Damme L, Corneli A, Ahmed K, Agot K, Lombaard J, Kapiga S, et al. Preexposure prophylaxis for HIV infection among African women. N Engl J Med. 2012;367(5):411–22. doi: 10.1056/NEJMoa1202614 22784040 PMC3687217

[7] Marrazzo JM, Ramjee G, Richardson BA, Gomez K, Mgodi N, Nair G, et al. Tenofovir-based preexposure prophylaxis for HIV infection among African women. N Engl J Med. 2015;372(6):509–18. doi: 10.1056/NEJMoa1402269 25651245 PMC4341965

[8] https://www.hptn.org/news-and-events/press-releases/hptn-083-study-demonstrates-superiority-cabotegravir-prevention-hiv

[9] https://www.hptn.org/news-and-events/press-releases/hptn-084-study-demonstrates-superiority-of-cab-la-to-oral-tdfftc-for

[10] Kwakwa HA, Bessias S, Sturgis D, Mvula N, Wahome R, Coyle C, et al. Attitudes Toward HIV Pre-Exposure Prophylaxis in a United States Urban Clinic Population. AIDS Behav. 2016;20(7):1443–50. doi: 10.1007/s10461-016-1407-9 27115399

[11] Luecke EH, Cheng H, Woeber K, Nakyanzi T, Mudekunye-Mahaka IC, van der Straten A, et al. Stated product formulation preferences for HIV pre-exposure prophylaxis among women in the VOICE-D (MTN-003D) study. J Int AIDS Soc. 2016;19(1):20875. doi: 10.7448/IAS.19.1.20875 27247202 PMC4887458

[12] Corneli A, Perry B, McKenna K, Agot K, Ahmed K, Taylor J, et al. Participants’ Explanations for Nonadherence in the FEM-PrEP Clinical Trial. J Acquir Immune Defic Syndr. 2016;71(4):452–61. doi: 10.1097/QAI.0000000000000880 26536315

[13] Hendrix CW. HIV Antiretroviral Pre-Exposure Prophylaxis: Development Challenges and Pipeline Promise. Clin Pharmacol Ther. 2018;104(6):1082–97. doi: 10.1002/cpt.1227 30199098 PMC6925668

[14] Ross J, Stover J. Use of modern contraception increases when more methods become available: analysis of evidence from 1982–2009. Glob Health Sci Pract. 2013;1(2):203–12. doi: 10.9745/GHSP-D-13-00010 25276533 PMC4168565

[15] Cottrell ML, Yang KH, Prince HM, Sykes C, White N, Malone S, et al. A Translational Pharmacology Approach to Predicting Outcomes of Preexposure Prophylaxis Against HIV in Men and Women Using Tenofovir Disoproxil Fumarate With or Without Emtricitabine. J Infect Dis. 2016;214(1):55–64. doi: 10.1093/infdis/jiw077 26917574 PMC4907409

[16] Passmore JA, Jaspan HB, Masson L. Genital inflammation, immune activation and risk of sexual HIV acquisition. Curr Opin HIV AIDS. 2016;11(2):156–62. doi: 10.1097/COH.0000000000000232 26628324 PMC6194860

[17] Emmanuel G, Folayan M, Undelikwe G, Ochonye B, Jayeoba T, Yusuf A, et al. Community perspectives on barriers and challenges to HIV pre-exposure prophylaxis access by men who have sex with men and female sex workers access in Nigeria. BMC Public Health. 2020;20(1):69. doi: 10.1186/s12889-020-8195-x 31941469 PMC6964078

[18] Ortblad KF, Chanda MM, Musoke DK, Ngabirano T, Mwale M, Nakitende A, et al. Acceptability of HIV self-testing to support pre-exposure prophylaxis among female sex workers in Uganda and Zambia: results from two randomized controlled trials. BMC Infect Dis. 2018;18(1):503. doi: 10.1186/s12879-018-3415-z 30286737 PMC6172754

[19] Whitfield THF, John SA, Rendina HJ, Grov C, Parsons JT. Why I Quit Pre-Exposure Prophylaxis (PrEP)? A Mixed-Method Study Exploring Reasons for PrEP Discontinuation and Potential Re-initiation Among Gay and Bisexual Men. AIDS Behav. 2018;22(11):3566–75. doi: 10.1007/s10461-018-2045-1 29404756 PMC6077114

[20] O’Keefe BR, Vojdani F, Buffa V, Shattock RJ, Montefiori DC, Bakke J, et al. Scaleable manufacture of HIV-1 entry inhibitor griffithsin and validation of its safety and efficacy as a topical microbicide component. Proc Natl Acad Sci U S A. 2009;106(15):6099–104. doi: 10.1073/pnas.0901506106 19332801 PMC2662964

[21] Garg AB, Nuttall J, Romano J. The future of HIV microbicides: challenges and opportunities. Antivir Chem Chemother. 2009;19(4):143–50. doi: 10.1177/095632020901900401 19374141

[22] Kreiss J, Ngugi E, Holmes K, Ndinya-Achola J, Waiyaki P, Roberts PL, et al. Efficacy of nonoxynol 9 contraceptive sponge use in preventing heterosexual acquisition of HIV in Nairobi prostitutes. JAMA. 1992;268(4):477–82. 1320133

[23] Roddy RE, Zekeng L, Ryan KA, Tamoufe U, Weir SS, Wong EL. A controlled trial of nonoxynol 9 film to reduce male-to-female transmission of sexually transmitted diseases. N Engl J Med. 1998;339(8):504–10. doi: 10.1056/NEJM199808203390803 9709043

[24] Van Damme L, Ramjee G, Alary M, Vuylsteke B, Chandeying V, Rees H, et al. Effectiveness of COL-1492, a nonoxynol-9 vaginal gel, on HIV-1 transmission in female sex workers: a randomised controlled trial. Lancet. 2002;360(9338):971–7. doi: 10.1016/s0140-6736(02)11079-8 12383665

[25] Abdool Karim SS, Richardson BA, Ramjee G, Hoffman IF, Chirenje ZM, Taha T, et al. Safety and effectiveness of BufferGel and 0.5% PRO2000 gel for the prevention of HIV infection in women. AIDS. 2011;25(7):957–66. doi: 10.1097/QAD.0b013e32834541d9 21330907 PMC3083640

[26] Skoler-Karpoff S, Ramjee G, Ahmed K, Altini L, Plagianos MG, Friedland B, et al. Efficacy of Carraguard for prevention of HIV infection in women in South Africa: a randomised, double-blind, placebo-controlled trial. Lancet. 2008;372(9654):1977–87. doi: 10.1016/S0140-6736(08)61842-5 19059048

[27] Halpern V, Ogunsola F, Obunge O, Wang CH, Onyejepu N, Oduyebo O, et al. Effectiveness of cellulose sulfate vaginal gel for the prevention of HIV infection: results of a Phase III trial in Nigeria. PLoS One. 2008;3(11):e3784. doi: 10.1371/journal.pone.0003784 19023429 PMC2582655

[28] Van Damme L, Govinden R, Mirembe FM, Guedou F, Solomon S, Becker ML, et al. Lack of effectiveness of cellulose sulfate gel for the prevention of vaginal HIV transmission. N Engl J Med. 2008;359(5):463–72. doi: 10.1056/NEJMoa0707957 18669425

[29] McCormack S, Ramjee G, Kamali A, Rees H, Crook AM, Gafos M, et al. PRO2000 vaginal gel for prevention of HIV-1 infection (Microbicides Development Programme 301): a phase 3, randomised, double-blind, parallel-group trial. Lancet. 2010;376(9749):1329–37. doi: 10.1016/S0140-6736(10)61086-0 20851460 PMC2956883

[30] Alexandre KB, Gray ES, Pantophlet R, Moore PL, McMahon JB, Chakauya E, et al. Binding of the mannose-specific lectin, griffithsin, to HIV-1 gp120 exposes the CD4-binding site. J Virol. 2011;85(17):9039–50. doi: 10.1128/JVI.02675-10 21697467 PMC3165825

[31] Mori T, O’Keefe BR, Sowder RC 2nd, Bringans S, Gardella R, Berg S, et al. Isolation and characterization of griffithsin, a novel HIV-inactivating protein, from the red alga Griffithsia sp. J Biol Chem. 2005;280(10):9345–53. doi: 10.1074/jbc.M411122200 15613479

[32] Fernandez-Romero JA, Deal C, Herold BC, Schiller J, Patton D, Zydowsky T, et al. Multipurpose prevention technologies: the future of HIV and STI protection. Trends Microbiol. 2015;23(7):429–36. doi: 10.1016/j.tim.2015.02.006 25759332 PMC4490993

[33] Nixon B, Stefanidou M, Mesquita PM, Fakioglu E, Segarra T, Rohan L, et al. Griffithsin protects mice from genital herpes by preventing cell-to-cell spread. J Virol. 2013;87(11):6257–69. doi: 10.1128/JVI.00012-13 23536670 PMC3648100

[34] Levendosky K, Mizenina O, Martinelli E, Jean-Pierre N, Kizima L, Rodriguez A, et al. Griffithsin and Carrageenan Combination To Target Herpes Simplex Virus 2 and Human Papillomavirus. Antimicrob Agents Chemother. 2015;59(12):7290–8. doi: 10.1128/AAC.01816-15 26369967 PMC4649164

[35] Politch JA, Cu-Uvin S, Moench TR, Tashima KT, Marathe JG, Guthrie KM, et al. Safety, acceptability, and pharmacokinetics of a monoclonal antibody-based vaginal multipurpose prevention film (MB66): A Phase I randomized trial. PLoS Med. 2021;18(2):e1003495. doi: 10.1371/journal.pmed.1003495 33534791 PMC7857576

[36] Morris GC, Wiggins RC, Woodhall SC, Bland JM, Taylor CR, Jespers V, et al. MABGEL 1: first phase 1 trial of the anti-HIV-1 monoclonal antibodies 2F5, 4E10 and 2G12 as a vaginal microbicide. PLoS One. 2014;9(12):e116153. doi: 10.1371/journal.pone.0116153 25546420 PMC4278856

[37] https://rsc.niaid.nih.gov/sites/default/files/daidsgradingcorrectedv21.pdf.

[38] Wallace A, Thorn M, Maguire RA, Sudol KM, Phillips DM. Assay for establishing whether microbicide applicators have been exposed to the vagina. Sex Transm Dis. 2004;31(8):465–8. doi: 10.1097/01.olq.0000135986.35216.ba 15273578

[39] Wallace AR, Teitelbaum A, Wan L, Mulima MG, Guichard L, Skoler S, et al. Determining the feasibility of utilizing the microbicide applicator compliance assay for use in clinical trials. Contraception. 2007;76(1):53–6. doi: 10.1016/j.contraception.2006.10.012 17586138

[40] Thurman AR, Kimble T, Herold B, Mesquita PM, Fichorova RN, Dawood HY, et al. Bacterial Vaginosis and Subclinical Markers of Genital Tract Inflammation and Mucosal Immunity. AIDS Res Hum Retroviruses. 2015;31(11):1139–52. doi: 10.1089/aid.2015.0006 26204200 PMC4651020

[41] Fichorova RN. Guiding the vaginal microbicide trials with biomarkers of inflammation. J Acquir Immune Defic Syndr. 2004;37 Suppl 3:S184–93. 16419271 PMC2643374

[42] Tate J, Ward G. Interferences in immunoassay. Clin Biochem Rev. 2004;25(2):105–20. 18458713 PMC1904417

[43] Villegas G, Calenda G, Zhang S, Mizenina O, Kleinbeck K, Cooney ML, et al. In Vitro Exposure to PC-1005 and Cervicovaginal Lavage Fluid from Women Vaginally Administered PC-1005 Inhibits HIV-1 and HSV-2 Infection in Human Cervical Mucosa. Antimicrob Agents Chemother. 2016;60(9):5459–66. doi: 10.1128/AAC.00392-16 27381393 PMC4997862

[44] Mukhopadhyay S, Liang Y, Hur H, Villegas G, Calenda G, Reis A, et al. Comparative transcriptome analysis of the human endocervix and ectocervix during the proliferative and secretory phases of the menstrual cycle. Sci Rep. 2019;9(1):13494. doi: 10.1038/s41598-019-49647-3 31530865 PMC6749057

[45] Begay O, Jean-Pierre N, Abraham CJ, Chudolij A, Seidor S, Rodriguez A, et al. Identification of personal lubricants that can cause rectal epithelial cell damage and enhance HIV type 1 replication in vitro. AIDS Res Hum Retroviruses. 2011;27(9):1019–24. doi: 10.1089/AID.2010.0252 21309617 PMC3161103

[46] Kizima L, Rodriguez A, Kenney J, Derby N, Mizenina O, Menon R, et al. A potent combination microbicide that targets SHIV-RT, HSV-2 and HPV. PLoS One. 2014;9(4):e94547. doi: 10.1371/journal.pone.0094547 24740100 PMC3989196

[47] Rodriguez A, Kleinbeck K, Mizenina O, Kizima L, Levendosky K, Jean-Pierre N, et al. In vitro and in vivo evaluation of two carrageenan-based formulations to prevent HPV acquisition. Antiviral Res. 2014;108:88–93. doi: 10.1016/j.antiviral.2014.05.018 24909570 PMC4116815

[48] Barnable P, Calenda G, Ouattara L, Gettie A, Blanchard J, Jean-Pierre N, et al. A MIV-150/zinc acetate gel inhibits SHIV-RT infection in macaque vaginal explants. PLoS One. 2014;9(9):e108109. doi: 10.1371/journal.pone.0108109 25259616 PMC4178065

[49] Richardson-Harman N, Lackman-Smith C, Fletcher PS, Anton PA, Bremer JW, Dezzutti CS, et al. Multisite comparison of anti-human immunodeficiency virus microbicide activity in explant assays using a novel endpoint analysis. J Clin Microbiol. 2009;47(11):3530–9. doi: 10.1128/JCM.00673-09 19726602 PMC2772583

[50] Aravantinou M, Singer R, Derby N, Calenda G, Mawson P, Abraham CJ, et al. The nonnucleoside reverse transcription inhibitor MIV-160 delivered from an intravaginal ring, but not from a carrageenan gel, protects against simian/human immunodeficiency virus-RT Infection. AIDS Res Hum Retroviruses. 2012;28(11):1467–75. doi: 10.1089/aid.2012.0080 22816564 PMC3484820

[51] Robinson MD, McCarthy DJ, Smyth GK. edgeR: a Bioconductor package for differential expression analysis of digital gene expression data. Bioinformatics. 2010;26(1):139–40. doi: 10.1093/bioinformatics/btp616 19910308 PMC2796818

[52] Mauck C, Chen PL, Morrison CS, Fichorova RN, Kwok C, Chipato T, et al. Biomarkers of Cervical Inflammation and Immunity Associated with Cervical Shedding of HIV-1. AIDS Res Hum Retroviruses. 2016;32(5):443–51. doi: 10.1089/AID.2015.0088 26650885 PMC4845652

[53] Schwartz JL, Mauck C, Lai JJ, Creinin MD, Brache V, Ballagh SA, et al. Fourteen-day safety and acceptability study of 6% cellulose sulfate gel: a randomized double-blind Phase I safety study. Contraception. 2006;74(2):133–40. doi: 10.1016/j.contraception.2006.02.008 16860051

[54] Derby N, Lal M, Aravantinou M, Kizima L, Barnable P, Rodriguez A, et al. Griffithsin carrageenan fast dissolving inserts prevent SHIV HSV-2 and HPV infections in vivo. Nat Commun. 2018;9(1):3881. doi: 10.1038/s41467-018-06349-0 30250170 PMC6155161

[55] Li Q, Estes JD, Schlievert PM, Duan L, Brosnahan AJ, Southern PJ, et al. Glycerol monolaurate prevents mucosal SIV transmission. Nature. 2009;458(7241):1034–8. doi: 10.1038/nature07831 19262509 PMC2785041

[56] Smith-McCune K, Chen JC, Greenblatt RM, Shanmugasundaram U, Shacklett BL, Hilton JF, et al. Unexpected Inflammatory Effects of Intravaginal Gels (Universal Placebo Gel and Nonoxynol-9) on the Upper Female Reproductive Tract: A Randomized Crossover Study. PLoS One. 2015;10(7):e0129769. doi: 10.1371/journal.pone.0129769 26177352 PMC4503751

[57] Schall TJ, Bacon K, Toy KJ, Goeddel DV. Selective attraction of monocytes and T lymphocytes of the memory phenotype by cytokine RANTES. Nature. 1990;347(6294):669–71. doi: 10.1038/347669a0 1699135

[58] Taub DD, Lloyd AR, Wang JM, Oppenheim JJ, Kelvin DJ. The effects of human recombinant MIP-1 alpha, MIP-1 beta, and RANTES on the chemotaxis and adhesion of T cell subsets. Adv Exp Med Biol. 1993;351:139–46. doi: 10.1007/978-1-4615-2952-1_15 7524282

[59] Cocchi F, DeVico AL, Garzino-Demo A, Arya SK, Gallo RC, Lusso P. Identification of RANTES, MIP-1 alpha, and MIP-1 beta as the major HIV-suppressive factors produced by CD8+ T cells. Science. 1995;270(5243):1811–5. doi: 10.1126/science.270.5243.1811 8525373

[60] Ozdemir V, Endrenyi L. A New Approach to Measure Adherence to Medicines Using Biomarkers and Sensors. OMICS. 2019;23(7):334–7. doi: 10.1089/omi.2019.0092 31199695

[61] Haaland RE, Chaowanachan T, Evans-Strickfaden T, van de Wijgert JH, Kilmarx PH, McLean CA, et al. Carrageenan-based gel retains limited anti-HIV-1 activity 8–24 hours after vaginal application by HIV-infected Thai women enrolled in a phase I safety trial. J Acquir Immune Defic Syndr. 2012;61(5):e71–3. doi: 10.1097/QAI.0b013e318271c8f9 23183152 PMC5549010

