# Supplementary figures and images for: Results of a phase 1, randomized, placebo-controlled first-in-human trial of griffithsin formulated in a carrageenan vaginal gel
Source: PLoS One. 2022 Jan 20;17(1):e0261775. doi: 10.1371/journal.pone.0261775 (PMC8775213; doi:10.1371/journal.pone.0261775)

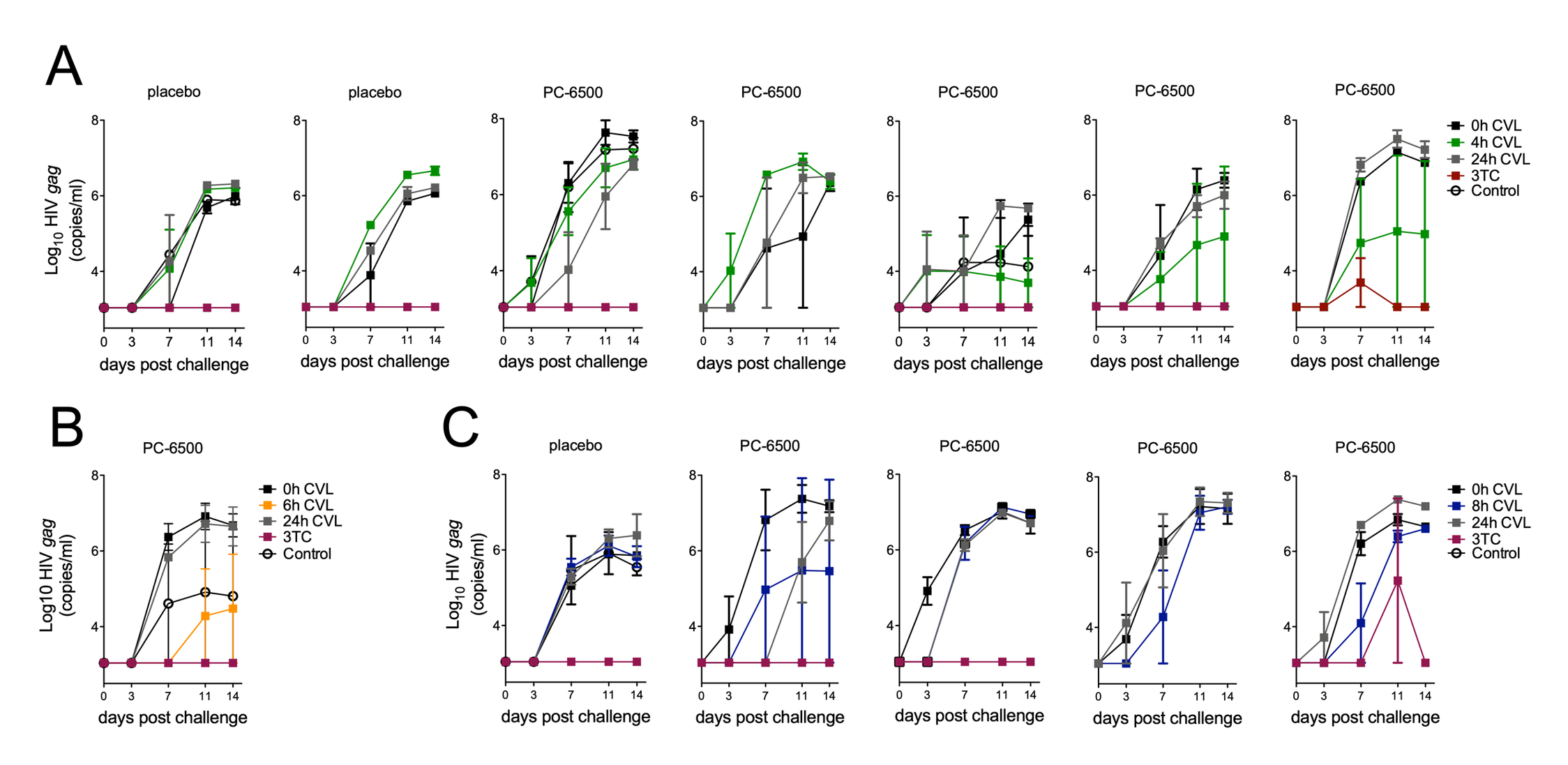

Supplement: S1 Fig — PHA/IL-2 activated polarized ectocervical explants were challenged with HIV-1BaL in the presence of CVLs collected at (A) BL (0h CVL), 4h post first gel dose (4h CVL) and 24h post last dose (24h CVL); (B) 0h, 6h post first gel dose (6h CVL) and 24h post last dose; (C) 0h, 8h post first gel dose (8h CVL) and 24h post last dose. Controls included tissues challenged in the presence of medium (Control) or 3TC (diluted in medium). All CVLs collected from an individual subject were tested using ectocervical tissues from a single tissue donor. Each graph represents results using CVLs from an individual subject. Shown are MEAN±SEM HIV gag copies/mL of two explants per condition. One subject in placebo group had single explants included in control and 3TC conditions. BL and 4h CVL samples from one subject in PC-6500 group were suspected to be switched at the time of collection. This has been adjusted for the presentation and statistical analysis. (TIF) [file pone.0261775.s001.tif]

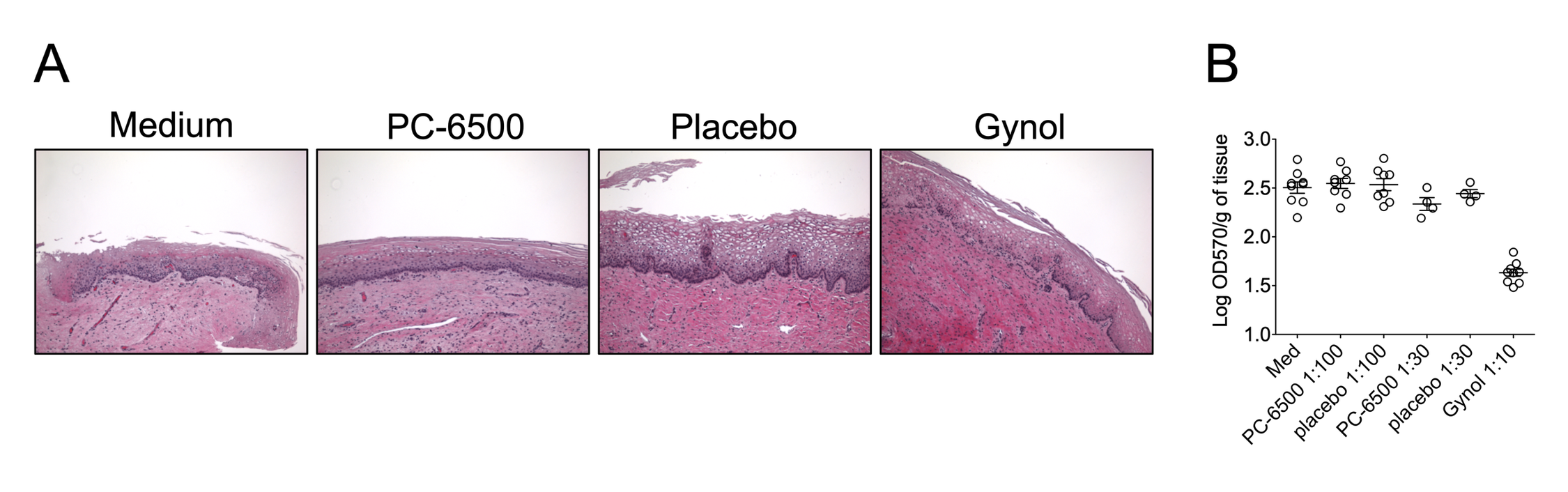

Supplement: S2 Fig — (A) Polarized human ectocervical explants were cultured for ~18h in the presence of neat gels vs. medium applied on the epithelium (single explant/condition). To assess epithelial integrity after exposure to the gels, tissues were washed, paraffin-embedded, and stained with H&E. Representative of at least 3 experiments is shown. (B) Alternatively, tissues were immersed in medium containing diluted PC-6500 (vs. medium, diluted placebo and Gynol controls) (n = 2–3 explants/condition). Tissue viability was determined using MTT assay (OD570 of the formazan product was normalized to the dry weight of the explants). Each symbol indicates an individual donor and the Mean±SEM of the Log10 OD570/g of tissue for each condition is shown. (TIF) [file pone.0261775.s002.tif]
