# Supplementary material for: Results of a phase 1, randomized, placebo-controlled first-in-human trial of griffithsin formulated in a carrageenan vaginal gel
Source: PLoS One. 2022 Jan 20;17(1):e0261775. doi: 10.1371/journal.pone.0261775 (PMC8775213; doi:10.1371/journal.pone.0261775)
Supplement: S1 File — (PDF) [file pone.0261775.s003.pdf]

**Study Title:**	A Phase 1 Trial to Evaluate the Safety, Pharmacokinetics (PK) and Pharmacodynamics (PD) of PC-6500 (Griffithsin [GRFT] in a Carrageenan Gel) in Healthy Women
**Study Number:**	Protocol 728
**IND Number:**	123512
**Registration:**	NCT 02875119
**Study Phase:**	1
**Product Name:**	PC-6500 (Griffithsin [GRFT] in a carrageenan gel)
**Sponsor:**	The Population Council, Inc.
**Study Manager:**	Barbara A. Friedland, MPH
**Medical Monitor:**	George W. Creasy, MD

**Version 1.0:**	Approved by Population Council IRB, 4/12/16
**Amendment 1**	Approved by Population Council IRB, 2/9/17<br>Approved by Einstein IRB, 8/7/17
**Amendment 2**	Approved by Population Council IRB, 1/18/18
**Amendment 3**	Approved by Population Council IRB, 5/30/18

This Protocol is provided for informational purposes only. The content of the Protocol is not intended to be relied upon for any research study other than PC-6500.

Statement of Compliance

The study will be carried out in accordance with Good Clinical Practice (GCP) as required by the following:

- *US Code of Federal Regulations applicable to clinical studies (CFR Title 21 Part 50 and Part 312)*
- *International Conference on Harmonization (ICH) GCP E6*

SIGNATURE PAGE

The signature below constitutes the approval of this protocol and the attachments, and provides the necessary assurances that this trial will be conducted according to all stipulations of the protocol, including all statements regarding confidentiality, and according to local legal and regulatory requirements and applicable US federal regulations and ICH guidelines.

Principal Investigator signature: ____________________________________

Principal Investigator (print name): ___________________________________

Date signed: ________________

TABLE OF CONTENTS

1. LIST OF ABBREVIATIONS IV
2. PROTOCOL SYNOPSIS 1
3. KEY ROLES 4
4. INTRODUCTION 7
5. STUDY PRODUCT 9
    5.1 Griffithsin (GRFT) 9
    5.2 PC-6500 (GRFT in a CG gel) 10
    5.3 Phase 1 clinical trial plan 13
    5.4 CG placebo (PC-535) 14
6. OBJECTIVES 14
    6.1 Primary 14
    6.2 Exploratory 14
7. STUDY DESIGN 14
    7.1 Rationale for Study Design 14
    7.2 Duration of Study 15
    7.3 Duration of Treatment 15
    7.4 Duration of Participation 15
    7.5 Description of Participation 15
    7.6 Endpoints 18
    7.7 Criteria for Proceeding to the Randomized Period (Main Study) 18
    7.8 Site 19
8. STUDY POPULATION 19
    8.1 Selection of Study Population 19
    8.2 Recruitment 19
    8.3 Inclusion Criteria 19
    8.4 Exclusion Criteria 20
9. STUDY PRODUCT USE 21
    9.1 Description of Study Products 21
    9.2 Justification, Selection, and Timing of Dose 22
    9.3 Summary Table of Study Product Use 22
    9.4 Method of Assigning Study Subjects to Study Intervention Groups 23
    9.5 Randomization 24
    9.6 Blinding 24
    9.7 Dispensing Study Product 24
    9.8 Concomitant Medications and Devices 25
    9.9 Restrictions 25
    9.10 Study Product Exposure 25
    9.11 Study Product Packaging and Labeling 26
    9.12 Study Product Storage and Accountability 26
10. STUDY PROCEDURES 27

10.1 Informed Consent Process 27
10.2 Medical History 27
10.3 Medication History 27
10.4 Documentation of HIV Status 28
10.5 Physical Examination 28
10.6 Pelvic Examination 28
10.7 Biopsies for Histology (randomized period only) 28
10.8 Clinical Laboratory Tests 28
*If dipstick urinalysis indicates abnormal results, urine specimen will be sent to MMC laboratory for complete urinalysis and, if indicated, urine culture. 29
10.9 Specimens for Research Purposes 29
11. STUDY EVALUATIONS: OPEN-LABEL PERIOD 30
11.1 OL Safety Period, Visit 0: Screening Visit (≤30 days before Enrollment) 30
11.2 OL Safety Period, Visit 1: Enrollment/Dosing (Day 1) 32
11.3 OL Safety Period, Visit 2: 24-Hour PK Visit (Day 2) 35
11.4 OL Safety Period, Visit 3: Safety Follow Up/Closing (Day 8) 36
12. STUDY EVALUATIONS: RANDOMIZED PERIOD 36
12.1 Randomized Period, Visit 0: Screening Visit (≤45 Days before Visit 1/Enrollment) 37
12.2 Randomized Period, Visit 1: Enrollment/Baseline (≥ 7 days before Visit 2/Day 1) 38
12.3 Randomized Period, Visit 2: Dose #1 (Day 1) 39
12.4 Randomized Period, Visits 3-5: Doses 3, 8, 11 (Days 3, 8 and 11) 42
12.5 Randomized Period, Visit 6: Final dose (Day 14) 43
12.6 Randomized Period, Visit 7: Final PK assessment (Day 15) 45
12.7 Randomized Period, Visit 8: Final Safety Visit (Day 21) 46
12.8 Randomized Period, Visit 9: Closing visit (Study Day 28) 47
12.9 Unscheduled Visits 47
13. OTHER STUDY EVALUATIONS/PROCEDURES 48
13.1 Pharmacokinetic/Pharmacodynamic Sampling 48
13.2 Biohazard Containment 48
13.3 Dye Stain Assay 48
13.4 Follow-up Procedures for Subjects who Discontinue Study Product 48
13.5 Subjects who Become Pregnant 48
13.6 Protocol Deviations and Violations 49
14. SAFETY ASSESSMENTS 49
14.1 Safety Monitoring 49
14.2 Adverse Events Definitions and Reporting Requirements 49
14.3 Serious Adverse Events Definitions and Reporting Requirements 50
14.4 Notification to Sponsor of Immediately Reportable Events (IRE) 52
14.5 Medical Emergencies and Emergency Protocol Deviations 53
14.6 Unblinding Procedures (randomized period only) 54
14.7 Sponsor Reporting Obligations 54

15. CLINICAL MANAGEMENT 54
15.1 Grading System 54
15.2 Dose Modification Instructions 55
15.3 Discontinuation of Study Product in Response to Adverse Events 55
15.4 Management of Specific Toxicities 55
15.5 Criteria for Early Termination of Study Participation 55
15.6 Subject Withdrawal 56
15.7 Pregnancy and Pregnancy Outcome 56
16. QUALITY CONTROL AND ASSURANCE 56
17. STATISTICAL METHODS 57
17.1 Overview and Summary of Design 57
17.2 General Considerations 57
17.3 Determination of Sample Size 57
17.4 Analysis Populations 57
17.5 Endpoints 57
17.6 Safety Analysis 58
17.7 Statistical Analysis of PK Measurements 59
17.8 Statistical Analysis of Exploratory Endpoints 59
17.9 Exposure Analysis 60
18. DATA HANDLING 60
18.1 Data Management Responsibilities 60
18.2 Source Documents 60
19. CLINICAL SITE MONITORING 61
20. HUMAN SUBJECTS PROTECTION 61
20.1 Institutional Review Boards 61
20.2 Study Coordination 62
20.3 Risk-Benefit Statement 62
20.4 Informed Consent Process 63
20.5 Confidentiality 63
21. PUBLICATION POLICY 64
REFERENCES 65
APPENDIX 1: SCHEDULE OF VISITS AND PROCEDURES FOR OL SAFETY RUN-IN (N=7) 68
APPENDIX 2: SCHEDULE OF VISITS AND PROCEDURES FOR RANDOMIZED PERIOD (N=20) 69
APPENDIX 3. DOCUMENTATION OF INFORMED CONSENT FOR OL SAFETY RUN-IN 72
APPENDIX 4. DOCUMENTATION OF INFORMED CONSENT FOR RANDOMIZED PERIOD 84
APPENDIX 5. DAIDS GRADING CRITERIA 99

1. LIST OF ABBREVIATIONS

ACOG	American Congress of Obstetricians and Gynecologists
ADA	Anti-drug antibodies
AE	Adverse event
AIDS	Acquired Immune Deficiency Syndrome
ALB	Albumin
ALK Phos	Alkaline phosphatase
ALT	Alanine aminotransferase (SGPT)
API	Active pharmaceutical ingredient
ARV	Antiretroviral
ASCUS	Atypical cells of undetermined significance
AST	Aspartate aminotransferase (SGOT)
AUC	Area under the time-concentration curve
β-hCG	Beta human chorionic gonadotropin
BUN	Blood urea nitrogen
BV	Bacterial vaginosis
Ca^{2+}	Calcium
CBC	Complete blood count
CBR	Center for Biomedical Research
CDC	Centers for Disease Control and Prevention
CFR	Code of Federal Regulations
CG	Carrageenan
Cl^-	Chloride
Cl/F	Clearance (apparent)
C_{max}	Maximum drug concentration
C_{min}	Minimum drug concentration
CRA	Clinical research associate
CRC	Clinical research center
CRF	Case report form
CT	Chlamydia trachomatis
CTA	Clinical trial agreement
CVL	Cervicovaginal lavage
DAIDS	Division of AIDS (NIH)
DSA	Dye stain assay
D-PBS	Dulbecco's phosphate buffered saline
EC_{50}	Half minimal effective concentration
EDC	Electronic data capture
EIA	Enzyme immunoassay
EKG	Electrocardiogram
ELISA	Enzyme-linked immunosorbent assay
FDA	Food and Drug Administration
FDI	Fast-dissolve insert
GC	Griffithsin carrageenan
GCP	Good Clinical Practice
GGT	Gamma glutamyl transferase
GLP	Good Laboratory Practice
GMP	Good Manufacturing Practice
GRAS	Generally recognized as safe
GRFT	Griffithsin

Hct	Hematocrit
HCV	Hepatitis C virus
HEC	Hydroxyethyl cellulose ("universal placebo")
Hgb	Hemoglobin
HIPAA	Health Information Portability and Accountability Act
HIV	Human Immunodeficiency Virus
HPV	Human papillomavirus
HSV-1	Herpes simplex virus-Type 1
HSV-2	Herpes simplex virus-Type 2
ICF	Informed consent form
ICH	International Conference on Harmonization
ICTR	Institute for Clinical and Translational Research
IND	Investigational New Drug
IRB	Institutional Review Board
IRE	Immediately reportable event
IUD	Intrauterine device
IUS	Intrauterine system
IV	Intravenous
IVR	Intravaginal ring
K^+	Potassium
KDa	Kilodaltons (molecular weight)
KOH	Potassium hydroxide
LDH	Lactic dehydrogenase
LLNA	Local lymph node assay
MedDRA	Medical Dictionary for Regulatory Activities
MLLNA	Murine local lymph node assay
MMC	Montefiore Medical Center
MOA	Mechanism of action
mRNA	Messenger RNA
MSD	Meso Scale Discovery
MTD	Maximum tolerated dose
MTT	3-(4,5-Dimethylthiazol-2-yl)-2,5-Diphenyltetrazolium Bromide
Na+	Sodium
NAAT	Nucleic acid amplification test
NCI	National Cancer Institute (NIH)
NIH	National Institutes of Health
NG	Neisseria gonorrhea
nM	Nanomolar
NNRTI	Non-nucleoside reverse transcriptase inhibitor
NOAEL	No observed adverse effect level
OC	Oral contraception/contraceptive
OHRP	Office for Human Research Protections
OL	Open label
OTC	Over-the-counter
PBMC	Peripheral blood mononuclear cells
PBS	Phosphate buffered saline
PD	Pharmacodynamics
PEP	Post-exposure prophylaxis
pfu	Plaque forming unit
PI	Principal Investigator
PK	Pharmacokinetics

PrEP	Pre-exposure prophylaxis
PsV	Pseudovirus
PT	Prothombin time
PTID	Participant ID number
PTT	Partial thromboplastin time
RBC	Red blood cell
RNA	Ribonucleic acid
RSID	Rapid Stain Identification (of human semen)
RT	Reverse transcriptase
RTI	Reproductive tract infection
RVI	Rabbit vaginal irritation (study)
SAE	Serious adverse event
SAP	Statistical analysis plan
SCM	Specimen collection manual
SHIV-RT	Simian/Human Immunodeficiency Virus
SOP	Standard operating procedure
SSP	Study specific procedures (manual)
STI	Sexually transmitted infection
TasP	Treatment as Prevention
TEAE	Treatment emergent adverse event
$T_{1/2}$	Half life
T_{max}	Time to maximum concentration
TPP	Target Product Profile
TV	Trichomonas vaginalis
UA	Urinalysis
UTI	Urinary tract infection
VMMC	Voluntary male medical circumcision
WBC	White blood cell

2. PROTOCOL SYNOPSIS

Study Title: A Phase 1 Trial to Evaluate the Safety, Pharmacokinetics (PK) and Pharmacodynamics (PD) of PC-6500 (Griffithsin [GRFT] in a Carrageenan [CG] Gel) in Healthy Women

Short Title: Safety, PK and PD of GRFT for vaginal use

Clinical Phase: Phase 1

IND Sponsor: Population Council, Inc.

Protocol Chair: George W. Creasy, MD

Sample Size: 27 female subjects (7 in open-label single-dose period; 20 in randomized, double-blind multiple-dose period)

Study Population: Healthy HIV-seronegative women aged 18-49 who are sexually abstinent during the study

Study Site: One US site, Albert Einstein College of Medicine (Einstein), Bronx, NY

Study Duration: Approximately 8 days of active participation per subject in the open label (OL) period (n=7) and 45 days of active participation per subject in the randomized, placebo-controlled, double-blind period (n=20); approximately 10 months for overall accrual.

Study Design: In the OL safety run-in period, 7 (seven) women will receive a single 4 g dose of PC-6500 (GRFT in a CG gel) with PK assessments through 12h and a final assessment on the following day at 24h, a PD assessment 24h post-dose; and a final safety assessment one week later (Day 8). After the first 7 women have received the single dose of PC-6500 gel and all safety/PK/PD assessments have been completed, there will be a pause in recruitment while data are reviewed. Data from the OL period will inform the subsequent PK and PD assessments in the main, randomized period of the study.

The main study will enroll 20 new subjects in a randomized, placebo-controlled, double-blind design to assess safety, PK and PD of 4 g PC-6500 used once daily for 14 consecutive days. Subjects in the main study will be randomized to PC-6500 gel (n=14) or PC-535 (CG placebo) gel (n=6). Subjects will also be randomized to one of two time points (4 hours or 8 hours) for blood and cervicovaginal lavage (CVL) specimen collection after Dose 1. At baseline (at least 7 days before Dose 1) and after Dose 14, biopsies, CVLs, and vaginal swabs will be collected to quantify concentrations of GRFT and CG (CVL); to measure antiviral activity in cell-based

assays and mucosal explants (CVL); to evaluate the impact of PC-6500 on gene expression in tissues (cervical biopsy), concentrations of genital tract immune mediators (CVL), impact on anti-*E. coli* activity (CVL), and the vaginal microbiome (vaginal swab); and to assess histological changes (vaginal and cervical biopsies). Antibodies will be measured before gel use, after 14 days of daily gel application (Day 14), one week after dosing ends (Day 21), and two weeks after dosing ends (Day 28) to assess potential anti-drug antibodies (ADA) against GRFT.

Study Products:

**Active Investigational Gel:**<br>0.1% GRFT (1 mg/1 mL)	**Test:** PC-6500 (GRFT in a carrageenan gel)<br>OL period: one dose of 4 g PC-6500 gel<br><br>Randomized period: 4 g PC-6500 gel, once daily for 14 consecutive days
**Placebo Gel:**<br>3% carrageenan gel	Randomized period: 4 g PC-535 (CG gel),<br>once daily for 14 consecutive days

Product Randomization and Time Point Stratification:

**Time point after Dose 1**	**GRFT Gel 4 g**	**CG Gel 4 g**	**TOTAL**
4h	n=up to 7	n=up to 3	n=10
8h	n=up to 7	n=up to 3	n=10
TOTAL	n=up to 14	n=up to 6	N=20

Primary Objectives:

1) To evaluate the **safety** of PC-6500 gel used vaginally for a single dose, and then for 14 consecutive days of dosing.
2) To assess the **pharmacokinetics (PK)** of GRFT in blood after a single dose, and then after 14 days of dosing.

Primary Endpoints:

1) Safety
   - Number and percent of subjects with treatment emergent adverse events (TEAEs), serious adverse events (SAEs), and TEAEs leading to premature discontinuation.
   - Number, percent and, in the investigator's judgment, medical significance of abnormalities in physical exams, pelvic exams, and biopsies once product has been administered.
   - Number, percent and, in the investigator's judgment, medical significance of abnormalities in clinical laboratory parameters.

2) Pharmacokinetics (PK) of GRFT
Evaluation of concentrations of GRFT in blood during and after 1 day (the initial dose); then during and after 14 days of daily use of GRFT vaginal gel, with estimates of:

- Area under the time-concentration curve ($AUC_{0\text{-last}}$; $AUC_{0\text{-}\infty}$)
- Peak concentration (C_{max})
- Trough concentration (C_{min})
- Time to peak concentration (T_{max})
- Elimination half-life ($T_{½}$)
- Apparent clearance (Cl/F)

Exploratory Objectives:
1) To determine the **concentrations** of GRFT and CG in CVLs after a single gel application and after 14 days of daily use of PC-6500 gel.
2) To determine the **pharmacodynamics (PD)** of GRFT and CG in CVLs.
3) To evaluate the impact of GRFT gel on **gene expression** in tissues and on concentrations of genital tract **immune mediators**.
4) To evaluate the impact of GRFT gel on the **vaginal microbiome**.
5) To assess whether topical use of GRFT gel leads to systemic **anti-drug antibodies** (ADA).
6) To measure the association of CVL drug levels with **anti-HIV activity**.
7) To assess the impact of GRFT on **anti-*E. coli* activity** in CVLs.

Exploratory Endpoints:
1) Post-treatment concentrations of GRFT and CG in CVLs.
2) Changes between baseline and after treatment in anti-HIV activity of CVLs in cell-based assays and mucosal explants.
3) Changes between baseline and after treatment in transcriptome analysis of gene expression in tissues and in concentrations of immune mediators in CVLs.
4) Changes between baseline and after treatment in the vaginal microbiome.
5) Changes between baseline and after treatment in ADA detection in blood.
6) Association between CVL drug levels with anti-HIV activity.
7) Changes between baseline and after treatment in E. coli activity in CVLs.

3. KEY ROLES

Site Principal Investigator
Marla J. Keller, MD
Professor, Departments of Medicine, Obstetrics & Gynecology and Women's Health
Albert Einstein College of Medicine
1300 Morris Park Avenue
Block Building, Room 512
Bronx, NY 10461
Tel: 718-430-3240
Fax: 718-430-8879
marla.keller@einstein.yu.edu

Protocol Chair/Medical Monitor
George W. Creasy, MD
Population Council
Center for Biomedical Research (CBR)
Weiss Building – 6th Floor
1230 York Ave.
New York, NY 10065
Tel: 212-327-8748
gcreasy@popcouncil.org

Population Council Team

Clinical Project Manager/Monitor
Barbara Friedland, MPH
Population Council (CBR)
1230 York Ave.
NY, NY 10065
Tel: 212-327-7045
bfriedland@popcouncil.org

Medical Safety
Mohcine Alami, MD
Director, Global Medical Safety
Population Council
4301 Connecticut Avenue, NW
Washington, DC 20008
Tel: 202-237-9410
malami@popcouncil.org

Senior Statistical and Data Quality Manager
Marlena Plagianos, MS
Population Council (CBR)
Weiss Building – 6th Floor
1188 York Ave.
New York, NY 10065
Tel: 212-327-8794
mplagianos@popcouncil.org

Regulatory Affairs
Daniel Loeven, MS
Acting Director, Global Regulatory Affairs for
Research Population Council (CBR)
Weiss Building – 6th Floor
1188 York Ave.
New York, NY 10065
Phone: 212-327-7188
dloeven@popcouncil.org

Population Council Co-Investigators:

José Fernández-Romero, PhD
Virology
Population Council (CBR)
Weiss Building – 4th Floor
1188 York Ave.
New York, NY 10065
Tel: 212-327-8729
jromero@popcouncil.org

Shweta Ugaonkar, PhD
Formulation and Manufacturing
Population Council (CBR)
Weiss Building – 4th Floor
1188 York Ave.
New York, NY 10065
Tel: 212-327-8355
sugaonkar@popcouncil.org

Natalia Teleshova, MD, PhD
Pharmacodynamics
Population Council (CBR)
Weiss Building – 5th Floor
1188 York Ave.
New York, NY 10065
Tel: 212-327-8759
nteleshova@popcouncil.org

Tom Zydowsky, PhD
Analytical and Formulation
Population Council (CBR)
Weiss Building – 4th Floor
1188 York Ave.
New York, NY 10065
Tel: 212-327-8744
tzydowsky@popcouncil.org

Einstein team

Jessica Atrio, MD
Assistant Professor
Department of Obstetrics & Gynecology
and Women's Health
1695 Eastchester Road
Bronx, NY 10461
Tel: 718-405-8200
jatrio@montefiore.org

Rebecca Barnett
Study Coordinator
Albert Einstein College of Medicine
1300 Morris Park Avenue
Block Building, Room 514
Bronx, NY 10461
Tel: 718-430-3253
Fax: 718-430-8879
rebecca.barnett@einstein.yu.edu

Laurie Ray, NP
Nurse Coordinator
Albert Einstein College of Medicine
1300 Morris Park Avenue
Block Building, Room 514
Bronx, NY 10461
Tel: 718-430-3061
Fax: 718-430-8879
laurie.ray@einstein.yu.edu

Eleftheria Zias, RPh
Clinical Research Pharmacist
Investigational Drug Services
Department of Pharmacy
Montefiore Medical Center - Weiler Division
1825 Eastchester Road
Bronx, NY 10461
718-904-2422 Office
718-904-2158 Fax
ezias@montefiore.org

Laboratory Locations:

Montefiore Medical Center
Department of Pathology
111 East 210th Street
Bronx, NY 10467

Population Council
Center for Biomedical Research (CBR)
Weiss Building – 4th and 5th Floors
1188 York Ave.
New York, NY 10065

Raina Fichorova, M.D., Ph.D.
Associate Professor
Harvard Medical School
Director, Laboratory of Genital Tract Biology
Department of Obstetrics, Gynecology and Reproductive Biology
Brigham and Women's Hospital
221 Longwood Avenue RF468
Boston, MA 02115
Office Tel: 617-278-0625
Lab Tel: 617-525-6845/6
Cell: 617-823-1598
GSM: 617-784-6403
Fax: 617-713-3018
rfichorova@rics.bwh.harvard.edu

Patrick M. Gillevet, Ph.D.
Director, Microbiome Analysis Center
Professor, Department of Biology
Affiliated Professor, School of Systems Biology
George Mason University
Prince William Campus
10900 University Boulevard, MSN 4D4
Room Occoquan-426
Manassas, Virginia 20110
Tel: 703-993-1057
Fax: 703-993-8430
pgilleve@gmu.edu

Tamara Kalir, MD, PhD
Associate Professor, Pathology
Assistant Professor, Obstetrics, Gynecology and Reproductive Science
Icahn School of Medicine
Department of Pathology
Box 1194
One Gustave L. Levy Place
New York, NY 10029
Tel: 212-241-3784
tamara.kalir@mountsinai.org

Dr. Yupu Liang
Director of Bioinformatics Program
Center for Clinical and Translational Science
Rockefeller University
1230 York Ave.
New York, NY 10065
Tel: 212-327-7923
liangy@rockefeller.edu

Dr. Betsy Herold Laboratory
Albert Einstein College of Medicine
1225 Morris Park Avenue
Van Etten Building, Room 6A-03
Bronx, NY 10461

4. INTRODUCTION

AIDS is the leading cause of death among women 15-44 years old, globally (UNAIDS 2014). Young women are disproportionately affected, particularly in sub-Saharan Africa, where HIV prevalence in some countries is more than seven times higher in 15-24-year-old females than in males of the same age (UNAIDS 2015). Despite the increase in biomedical prevention strategies that have been found effective in the last few years, most do not meet women's needs. Voluntary medical male circumcision (VMMC) has been shown to reduce the risk of HIV infection in males by 60% or more (Auvert et al. 2005; Bailey et al. 2007; Gray et al. 2007), but does not reduce women's risk (Wawer et al. 2009). Multiple trials of antiretrovirals (ARVs) used by HIV-infected or uninfected individuals have demonstrated effectiveness, yet there are an array of challenges limiting their utility for many women at risk of HIV. For example, treatment as prevention (TasP) has been shown in clinical trials to nearly eliminate HIV transmission from an infected person to his or her partner (Cohen et al. 2011), yet it requires the HIV-infected partner to know his or her status, to be on ARV treatment, to be adherent to treatment and to be virally suppressed, all of which are out of control of the uninfected partner. A number of trials have also demonstrated that ARVs taken daily or intermittently for pre-exposure prophylaxis (PrEP) can reduce HIV risk by as much as 86% (Grant et al. 2010; Baeten et al. 2012; Thigpen et al. 2012; McCormack et al. 2016; Molina et al. 2015). Despite promising trial results, PrEP is not yet available in many countries where women are at greatest risk. In addition, data have shown that fear of side effects or stigmatization (by people assuming PrEP users are already HIV positive) may prevent women from being adherent to daily PrEP use (van der Straten et al. 2015). Therefore, alternative prevention methods are needed that women will find feasible and acceptable.

For nearly 25 years, scientists have been working to develop vaginal microbicides to expand women's options to protect themselves against HIV and other sexually transmitted infections (STIs) (Elias et al. 1994). The World Health Organization estimates that more than one million people contract STIs each day (Fernández-Romero et al. 2015); human papillomavirus (HPV) and herpes simplex virus (HSV), in particular, are non-curable viral STIs representing a huge public health burden. Microbicides are products designed to be applied topically to vaginal and/or rectal tissue that can be used discretely by females (or males) to protect themselves against HIV and other STIs. Products that protect women (and men) against HIV and other STIs that increase susceptibility to HIV, such as herpes simplex virus-type 2 (HSV-2), human papillomavirus (HPV) and *Trichomonas vaginalis* (TV), would make a major contribution to public health globally. The current pipeline of microbicide candidates includes ARV-based and non-ARV-based products being developed in multiple delivery modes (such as gels, tablets, films, injectables and intravaginal rings [IVRs]) for on-demand or long-acting use (Garg et al. 2010). Of the first candidate microbicides to complete advanced-stage testing (Tolley et al. 2014), all formulated as intravaginal gels, only the ARV-based 1% tenofovir gel was shown to reduce the risk of HIV acquisition in women in the CAPRISA 004 trial (Abdool Karim et al. 2010). However, two subsequent Phase 3 trials of vaginally applied 1% tenofovir gel found no reduction in HIV risk (FACTS 2015; Marrazzo et al. 2015). Currently, the most promising candidate microbicide is a one-month IVR containing the non-nucleoside reverse transcriptase inhibitor (NNRTI), dapivirine. In two recently completed Phase 3 efficacy trials, HIV risk was reduced by 30% in women assigned to the dapivirine IVR compared to those using a non-medicated IVR (Baeten et al. 2016; Nel et al. 2016).

Although the current ARV-based microbicides in development will be an important addition to the toolbox of HIV-prevention methods for women, a microbicide that does not contain ARVs (particularly those used for treatment) offers several potential advantages. First, non-ARV-based

products are less likely to need labeling that requires HIV screening prior to and during use (once safety has been demonstrated in HIV-positive individuals). Second, a product without an ARV reduces the likelihood of emergence of drug-resistant HIV viruses that may compromise subsequent ARV treatment options. Third, a non-ARV-based microbicide is likely to be approved more quickly by regulatory authorities as an over-the-counter (OTC) product than one with ARVs. To this end, the Population Council is developing griffithsin (GRFT) as a microbicide for sustained-delivery or on-demand use with the support of the US Agency for International Development (USAID; Cooperative Agreement number AID-OAA-A-14-00009). The Council's overarching goal is to develop a product that prevents HIV and other STIs, including HSV, HPV and TV, which is affordable through public-sector programs.

The aim of this first-in-human trial of GRFT formulated in a CG gel (PC-6500) is to support the further development of GRFT for both on-demand use (such as in a fast-dissolve insert [FDI]) and sustained release, such as via an IVR. It is expected that the results of the trial will demonstrate that GRFT is safe and well tolerated and that there is negligible systemic absorption of GRFT in healthy HIV-negative women. The data from this trial will be important for informing decisions about doses for future trials and other formulations.

5. STUDY PRODUCT

5.1 Griffithsin (GRFT)

GRFT is a novel anti-HIV protein that was discovered at the National Cancer Institute (NCI) of the US National Institutes of Health (NIH) through a program evaluating natural products for their potential anti-HIV activity (Mori et al. 2005). GRFT was initially isolated from an aqueous extract of the red alga *Griffithsia* sp. (Mori et al. 2005) and can be produced in multigram quantities in tobacco plants (O'Keefe et al. 2009). GRFT's anti-HIV activity, which is even stronger than other promising lectins (Huskens et al. 2012), has prompted its development as a candidate microbicide to prevent HIV. GRFT has been shown to block both cell-free and cell-associated HIV entry into target cells (Huskens et al. 2012). Cell-bound GRFT has also been shown to retain its antiviral activity *in vitro* (Kouokam et al. 2011). GRFT has been shown to block HIV replication *in vitro* at subnanomolar (EC50 values 0.02 to 0.8nM) concentrations (Mori et al. 2005; Emau et al. 2007). Both *in vitro* and *in vivo* studies have demonstrated that GRFT also has activity against HSV-2 (Nixon et al. 2013; Levendosky et al. 2015), hepatitis C virus (HCV) (Meuleman et al. 2011; Takebe et al. 2013), and TV (Chatterjee et al. 2015). In addition, GRFT has been shown to have moderate *in vitro* activity (~1µM EC50) against HPV16, 18, and 45 pseudoviruses (PsVs) (Levendosky et al. 2015).

GRFT is a 12.77 KDa (121-amino acid sequence) carbohydrate-binding lectin that has specificity for high-mannose carbohydrates on the surface of HIV and other enveloped viruses, including HCV, severe acute respiratory syndrome coronavirus (SARS-CoV), and Ebola (Mori et al. 2005). GRFT's mechanism of action has been well characterized. GRFT targets viral entry by binding to high-mannose oligosaccharides on gp120 (Ziolkowska et al. 2006; Ziolkowska et al. 2007; Banerjee et al. 2012). GRFT does not impede binding of HIV to CD4, but prevents gp120 interaction with HIV co-receptors (Alexandre et al. 2011). The dimeric nature of GRFT, with three carbohydrate-binding sites per monomer, may result in HIV aggregation via multivalent interactions between GRFT and gp120 oligosaccharides (Moulaei et al. 2010).

Initial studies indicate that GRFT resists degradation by several proteases *in vitro*, is stable even at high temperatures, is non-irritating in rabbit vaginal irritation (RVI) studies, does not impact cell viability, does not induce pro-inflammatory responses in human peripheral blood mononuclear cells (PBMCs) or epithelial cells, and does not activate T-cells (O'Keefe et al. 2009; Kouokam et al. 2011; Moncla et al. 2011; Huskens et al. 2012). GRFT has a high genetic barrier that avoids the rapid selection of viral resistance (Huskens et al. 2012).

5.1.1 GRFT safety

GRFT was found to be well tolerated when applied subcutaneously to mice as a single dose (50mg/kg) and to guinea pigs (10mg/kg) as chronic dosing (Barton et al. 2014). No experimental animals died as a result of treatment with GRFT, nor were any behavior changes observed. Additionally, intra-peritoneal or subcutaneous treatment with GRFT in mice in a study to assess protection against Japanese encephalitis virus (Ishag et al. 2013) or HCV infection (Meuleman et al. 2011; Takebe et al. 2013) demonstrated that GRFT is tolerated by mice exposed to doses of 5 mg/kg of body weight.

5.1.2 GRFT PK, metabolism and distribution

PK of GRFT after intravenous (IV) or subcutaneous single dosing (10mg/kg or 20mg/kg) in rats has been previously reported (Barton 2014). The PK results in rats showed multiphasic elimination after IV dosing with detectable GRFT in serum at low ng/ml up to 96h post-treatment.

The same study revealed an absorption half-life of 0.5h and late phase elimination half-life between 10 to 17h. Subcutaneous application of GRFT resulted in systemic distribution and high levels of accumulation in blood. GRFT isolated from blood samples retained its *in vitro* antiviral activity against HIV-1 (Barton et al. 2014).

An acute IV study was conducted wherein a single bolus IV injection of GRFT in male and female Sprague-Dawley rats was evaluated by MPI Research (Mattawan, MI). Doses of 6.75, 33.75 and 67.5 mg/kg were well tolerated with no adverse effects noted. The maximum tolerated dose (MTD) is estimated to be at least 67.5 mg/kg – the highest dose tested (Population Council, unpublished data).

5.1.3 Clinical studies

No clinical studies of GRFT have been done.

5.1.4 Carrageenan (CG) as a delivery vehicle

Carrageenan (CG) is the vehicle for GRFT in the PC-6500 gel formulation. CG provides excellent rheological properties, including a viscosity that enables the gel to spread throughout the vagina (Fernández-Romero et al. 2012). CG is derived from seaweed and is generally recognized as safe (GRAS) for consumption and topical application by the US Food and Drug Administration (FDA). Six clinical trials of Carraguard, a CG gel nearly identical to the type used in PC-6500, conducted in over 6,000 women, provide extensive safety data on CG for vaginal use. In all trials, rates of AEs (nearly all categorized as mild) were similar between the CG and placebo groups, with vaginal discharge the most commonly reported event (Kilmarx et al. 2006; van de Wijgert et al. 2007; Kilmarx et al. 2008; Skoler-Karpoff et al. 2008; Carraguard Phase II South Africa Study Team 2010; McLean et al. 2010).

5.2 PC-6500 (GRFT in a CG gel)

PC-6500 is an investigational microbicide candidate comprised of GRFT formulated in a CG gel, which is being evaluated to prevent HIV infection. CG is a gelling agent and has potent antiviral (HPV and HSV) properties. GRFT combined with CG results in increased potency over either active pharmaceutical ingredient (API) alone (GRFT or CG) against HSV as well as against HPV (Levendosky et al. 2015).

5.2.1 *In vitro* and *in vivo* safety of GRFT and PC-6500

GRFT does not cause damage to vaginal or cervical tissues based on *in vivo* and *ex vivo* models. In a previously described immersion model (Barnable et al. 2014), the viability of macaque vaginal tissues following ~18h incubation was compared among unformulated GRFT (0.1-10 μM) and 0.1% GRFT formulated in a 3% CG gel (PC-6500). GRFT alone or formulated in a CG gel did not decrease viability of macaque vaginal tissues at the tested GRFT concentrations and gel dilutions. A log-normal generalized linear mixed model predicting the weight normalized OD_{570} was used. Both unformulated GRFT (up to 10 μM) and the gel formulations had similar safety profiles, as measured by the MTT (tetrazolium salt) assay. Macaque vaginal tissue remained viable with no significant differences detected between unformulated GRFT, the gels, and the respective untreated medium controls (Population Council, unpublished data).

The viability of human ectocervical tissue explants after exposure to CG placebo and PC-6500 was also evaluated to determine if these formulations induce histological changes (Barnable et al. 2014). No decrease in ectocervical tissue viability and no histopathological changes were detected after immersion of tissues in PC-6500 diluted gel (1:100), or after application of neat PC-6500 on the epithelial surface of explants, respectively.

In an HSV-2 mouse infection enhancement model (Fernández-Romero et al. 2012), PC-6500 did not enhance the susceptibility of mice to HSV-2 infection when compared to the D-PBS control (p=0.7152) (Population Council, unpublished data). In a separate experiment, a histological examination of cervicovaginal mucosa after a single vaginal application of PC-6500 in mice revealed no signs of damage to the epithelial architecture. As expected (Fernández-Romero et al. 2012), the Conceptrol reference control profoundly damaged the cervicovaginal tissue (peak damage at 6h, repaired by 24h) as evidenced by epithelial sloughing and exposure of the lamina propria (Population Council, unpublished data). PC-6500 administered vaginally daily for seven days in rhesus macaques resulted in no histopathological changes in cervical and vaginal mucosa, and there were no significant changes in vaginal pH or immune mediators detected (Population Council, unpublished data).

CG alone has not been found to have deleterious effects on vaginal lactobacilli (Moncla et al. 2012). Using the same methods, GRFT had no impact on lactobacilli viability (<1 log_{10} reduction).

5.2.2 Antiviral activity of GRFT and PC-6500

The antiviral activity of GRFT, PC-6500 and CG have been evaluated against different HIV-1 clades that included primary isolates, clones and founder/transmitted viruses in PBMCs using a previously described method (Kizima et al. 2014). GRFT and PC-6500 showed broad and selective antiviral activity against HIV independent of clade, tropism or phenotype. Carrageenan exhibited no antiviral activity, while GRFT or PC-6500 were still potent (EC_{50} values average 0.5nM based on GRFT concentration or ~0.000004 [1/250,000] based on PC-6500 gel dilution). These potent EC_{50} values combined with the lack of cytotoxicity resulted in high therapeutic index (TI>100) values against a spectrum of HIV strains/isolates. Additionally, varying doses of GRFT were tested in formulations containing different gelling agents (0.25% GRFT in CG, 0.1% GRFT in CG [the formulation in PC-6500] and 0.1% GRFT in Carbopol) using the human cervical explant model. All gel formulations achieved comparable activity against HIV in human cervical mucosa independent of the GRFT dose or gelling agent. In the macaque vaginal explant model, GRFT was found to be active against HIV cell-free and cell-associated virus (Population Council, unpublished data).

More recently, PC-6500 was tested *in vivo* to determine if it is more effective in blocking HSV-2 and HPV infection than either GRFT or CG alone, as had previously been demonstrated *in vitro* in stringent, high viral dose models (Levendosky et al. 2015). When Depo-Provera-treated mice were challenged vaginally with 10^6 plaque forming units (pfu) of HSV-2 or $8x10^6$ HPV16 PsV after applying each compound alone (1.9% GRFT or 3% CG) or in combination (0.1% GRFT: 3% CG), neither GRFT nor CG alone afforded significant protection against HSV-2. However, PC-6500 applied 10 minutes or 1h before virus challenge significantly reduced HSV-2 infection (p=0.0352) relative to CG or GRFT alone given 10 minutes prior to challenge (Levendosky et al 2015). Supporting GRFT's moderate EC_{50} against HPV, and its potential mechanism of action (MOA) (Levendosky et al. 2015), GRFT solution significantly protected mice against vaginal HPV when dosed during and after HPV16 PsV challenge (p<0.026 relative to hydroxyethyl cellulose [HEC] "universal placebo"). Much greater protection was afforded by CG alone for up to 8h (p=0.0022 relative to HEC placebo) and the contribution of GRFT in the PC-6500 formulation to this activity could not be determined. Thus, the potent activity of CG in this model masked any additional anti-HPV effect of GRFT in PC-6500 gel.

The window of protection against HSV-2 was evaluated in the vaginal mouse model under less stringent conditions, by applying PC-6500 gel or HEC placebo gel at different times before or after viral challenge. A significant decrease in the percentage of mice infected was observed when PC-6500 versus HEC was administered vaginally at least 4h before infection (p<0.0001), but not when applied 8h before (p=0.2148) or 30 minutes after (p=1.0000) virus challenge (Levendosky et al. 2015). Finally, seminal plasma had no effect on the anti-HSV-2 activity of PC-6500 gel in mice. The impact of seminal plasma on anti-HPV activity was not tested because CG's anti-HPV activity in mice co-exposed with seminal plasma had already been confirmed (Rodriguez et al. 2014).

5.2.3 Preclinical safety and PK/PD of PC-6500

Safety and PK/PD of various GRFT concentrations formulated in 3% CG gel were studied in rhesus macaques (Population Council, unpublished data). Macaques were dosed daily for seven days with 0.01% GRFT, 0.1% GRFT or 0.25% GRFT. No significant changes in vaginal pH, cytokine/chemokine profiles and tissue integrity associated with administration of GC (griffithsin carrageenan gel) or CG gels were detected. Although the data suggested that GC and CG treatments may have different effects on certain bacteria taxonomic groups, these changes were not significant for any comparisons within the study. To assess activity of tissue-associated GRFT, vaginal mucosa was collected 24h post-last gel administration and challenged with SHIV-RT *ex vivo*. Although anti-SHIV activity in the tissues from gel-treated animals was comparable to the placebo (3% CG only) group at this time point, vaginal fluids collected at the same 24h time point potently inhibited *in vitro* HIV infection in samples collected from the 0.1% GRFT or 0.25% GRFT groups. Vaginal fluids from 0.25% and 0.1% GRFT gel groups significantly inhibited HIV-1 *in vitro*, when compared to the 0.01% gel (p<0.005) or CG gel (p<0.001) groups. GRFT concentrations and EC_{50} values in vaginal fluids were strongly correlated (r=0.9691; p<0.0001).

5.2.4 *In vivo* toxicology

The toxicology program for GRFT and PC-6500 was based on FDA guidance obtained during Type B and Type C consultations. The following Good Laboratory Practice (GLP) studies have been completed by the contract research organization, MPI Research (Mattawan, MI).

<u>Local lymph node assay (LLNA), mice</u>: This study evaluated placebo and test gels containing 1 mg/mL (0.1%, PC-6500), 2 mg/mL (0.2%; 2x the clinical concentration), or 3 mg/mL (0.3%; 3x the clinical concentration) GRFT, as well as an additional preparation containing 3 mg/mL GRFT

(0.3%; 3x the clinical concentration) with approximately 20% of the GRFT oxidized, for the potential to cause skin sensitization (allergic contact dermatitis) via the Murine Local Lymph Node Assay (MLLNA). The placebo and all test articles were not identified as potential sensitizers.

14-day vaginal irritation study, rabbits: This study evaluated systemic and local toxicity and vaginal irritation in rabbits following 14 days of once-daily vaginal application of the following gels: placebo and test gels containing 1 mg/mL (0.1%, PC-6500), 2 mg/mL (0.2%; 2x the clinical concentration), or 3 mg/mL (0.3%; 3x the clinical concentration) GRFT, as well as an additional preparation containing 3 mg/mL GRFT (0.3%; 3x the clinical concentration) with approximately 20% of the GRFT oxidized. There were no adverse findings. The no observable-adverse-effect-level (NOAEL) was considered to be the 0.3% GRFT gel (3x the clinical concentration) or 0.3% GRFT gel with 20% of the GRFT oxidized.

14-day rat IV study: This study evaluated systemic and local toxicity, toxicokinetics, and ADA development in rats injected once daily for 14 days with GRFT solution at the following concentrations: 2.1, 4.15, and 8.3 mg/kg. There were no test article-related findings related to cage-side and detailed clinical observations, body weights, food consumption, clinical labs, organ weights, or macroscopic and microscopic pathology. All ADA samples were negative. Systemic exposure to GRFT was independent of sex. Area under the concentration-time curve from 0-24 hours (AUC 0-24) and maximum concentration (C_{max}) values of GRFT increased with increasing dose in a greater than dose-proportional manner across the dose range on Days 1 and 15. The NOAEL was considered to be 8.3 mg/kg/day, the highest dose tested.

14-day rat gel study: The study evaluated systemic and local toxicity and toxicokinetics in rats following 14 days of once-daily vaginal application of the following gels: placebo and test gels containing 1 mg/mL (0.1%; PC-6500), 2 mg/mL (0.2%; 2x the clinical concentration), or 3 mg/mL (0.3%; 3x the clinical concentration) GRFT, as well as an additional preparation containing 3 mg/mL GRFT (0.3%; 3x the clinical concentration) with approximately 20% of the GRFT oxidized. There were no adverse findings. There was little to no observed systemic exposure to GRFT. Serum concentrations were quantifiable in a single sample from the 0.3% GRFT gel (3x) with the 20% oxidized group, 2 samples from a single animal in the 0.3% GRFT (3x) gel group, and 2 animals (at one time point each) in the 0.2% GRFT (2x) gel group. No GRFT was detected in any animal in the 1x group. The NOAEL was considered to be the 3 mg/mL dose (3x the clinical concentration) or 3 mg/mL with 20% oxidized GRFT.

5.3 Phase 1 clinical trial plan

A two-part design is planned for this first-in-human trial of PC-6500. The Population Council does not plan to develop PC-6500 gel beyond this Phase 1 trial. This first-in-human trial is being conducted to inform development of GRFT in other formulations, such as IVRs and FDIs.

5.4 CG placebo (PC-535)

The placebo in this trial is PC-535, which is the CG vehicle for PC-6500. As indicated above in Section 5.1.4, CG is considered to be GRAS by the FDA and there is a large body of evidence supporting the safety of CG for vaginal use.

6. OBJECTIVES

6.1 Primary

1) To evaluate the **safety** of PC-6500 gel used vaginally for a single dose, and then for 14 consecutive days of dosing.
2) To assess the **pharmacokinetics (PK)** of GRFT in blood after a single dose, and then after 14 days of dosing.

6.2 Exploratory

1) To determine the **concentrations** of GRFT and CG in CVLs after a single gel application and after 14 days of daily use of PC-6500 gel.
2) To determine the **pharmacodynamics** (**PD)** of GRFT and CG in CVLs.
3) To evaluate the impact of GRFT gel on gene expression in tissues and on concentrations of genital tract **immune mediators**.
4) To evaluate the impact of GRFT gel on the **vaginal microbiome**.
5) To assess whether topical use of GRFT gel leads to systemic **anti-drug antibodies** (ADA).
6) To measure the association of CVL drug levels with **anti-HIV activity**.
7) To assess the impact of GRFT on **anti-*E. coli* activity** in CVLs.

7. STUDY DESIGN

This is a two-part study. The first part is a single-dose open label (OL) design. The second part employs a randomized, placebo-controlled, double-blind, multiple-dose study design.

7.1 Rationale for Study Design

GRFT is a large biologic molecule that is intended for vaginal rather than systemic administration. In addition, GRFT is being developed for the prevention of HIV infection and not as a therapeutic agent. The concentration of GRFT associated with prevention of HIV *in vitro* and *in vivo* has been established in non-clinical studies (see Section 5). Studies have demonstrated that GRFT is highly potent for HIV prevention and is effective at very low concentrations.

One 4 g dose of PC-6500, designed to provide an adequate vaginal concentration of GRFT for the prevention of HIV, based on preclinical data, will be evaluated. Rising dose tolerance is not the goal of this study because GRFT is likely to be minimally absorbed systemically, if at all. The evaluation of the dose selected (4 g gel formulation) will begin with a single-dose administration of PC-6500 in the OL period to evaluate safety and the extent of absorption. Following the evaluation of the absorption and the safety from the single-dose administration, a 14-day,

randomized, double-blind, placebo-controlled, multiple-dose period will proceed with the same 4 g dose formulation.

7.2 Duration of Study

The study is expected to last approximately 10 months from screening of the first subject in the single-dose OL period through the last subject visit in the randomized period.

7.3 Duration of Treatment

In the OL period, duration of treatment is one day. Each of the seven subjects will receive a single dose of PC-6500. In the randomized period, duration of treatment will be 14 days; each of the subjects will self-administer one of the two assigned study products (PC-6500 or PC-535 [CG placebo]) once daily for 14 consecutive days, with five doses administered under clinical supervision and the remaining nine doses administered at home.

7.4 Duration of Participation

Safety run-in (OL period):
Each subject will have four scheduled visits, including screening (see Figure 1). The screening visit (Visit 0) will be up to 30 days prior to enrollment. Once eligibility is confirmed and the subject is enrolled (Visit 1/Day 1), active follow-up will be eight days and will include two additional visits: Visit 2/Day 2 and Visit 3/Day 8. Total duration of study participation will be up to 40 days, including screening.

Main study (randomized period):
Each subject will have 10 scheduled visits, including screening (see Figure 2). The screening visit (Visit 0) can take place up to 45 days prior to enrollment. Subjects will be enrolled at Visit 1/Enrollment but will not begin dosing until the following week (after biopsies have healed). Dosing begins on Visit 2/Day 1 and lasts 14 days. Once dosing begins, subjects will attend the clinic for: Visit 3/Day 3, Visit 4/Day 8, Visit 5/Day 11, Visit 6/Day 14, Visit 7/Day 15, Visit 8/Day 21 (final safety assessment) and Visit 9/Day 28 (blood draw for ADA testing). Active follow-up from enrollment through study exit will be 35-45 days, depending on the duration of menses. Total study duration will be up to 80-90 days, including screening.

7.5 Description of Participation

Please see Sections 10-12 for detailed procedures and Appendix 1 and Appendix 2 for Schedule of Study Visits and Evaluations.

Safety run-in (OL) period:
Each subject will undergo a screening visit (Visit 0) up to 30 days prior to enrollment (see Figure 1). If eligible, subjects will have three additional scheduled visits: an enrollment/dosing visit (Visit 1), an outpatient blood draw visit (Visit 2), and a safety follow-up/closing visit (Visit 3).

At Visit 1 (Day 1), the subject will undergo a pelvic examination with CVL and vaginal microbiome sampling, after which the clinician will insert one dose of gel vaginally. The subject will remain in the clinic for 12 hours after dosing for multiple blood draws for PK assessments. After the 12-hour blood draw, the subject will be discharged from the clinic and can go home once the study staff has assessed that she tolerated the dose. Subject must have an adult (≥18 years old) who can stay overnight with her until she attends the clinic for the 24h blood draw the following day.

At Visit 2 (Day 2), the subject will return to the clinic for an outpatient visit including a 24-hour blood PK draw (+/-15 minutes within 24 hours of the single dose), safety labs, CVL and vaginal microbiome sampling.

At Visit 3 (Day 8), subjects will return for a final outpatient visit where they will undergo a pelvic examination and a final blood draw for safety labs.

Assessment of vital signs and AEs will occur before, during and after the single dose. Electrocardiograms (EKGs) will be done at enrollment (Visit 1) and on Day 2 (Visit 2), 24 hours after the single dose. Subjects will be asked to refrain from vaginal product use and vaginal activity, including intercourse, from 48 hours prior to Enrollment (Visit 1) through study exit on Day 8 (Visit 3).

Figure 1. STUDY SCHEMA – OL RUN-IN PERIOD

**Visit 0**<br>**Screening (up to 30 days <Enrollment)**		**Visit 1**<br>**Day 1**<br>**Enrollment**	**Visit 2**<br>**Day 2**<br>**24h PK Follow-up**	**Visit 3**<br>**Day 8**<br>**Closing Visit**
	**Begin abstinent 48h before enrollment**	SINGLE DOSE IN CLINIC		
		Multiple PK blood draws through 12h post-dose<br>CVL and vaginal microbiome before dose<br>Safety labs	24h blood draw after dose<br>Safety labs<br>CVL and vaginal microbiome	Pelvic exam<br>Safety labs

Main study (Randomized period):

Each subject will undergo screening up to 45 days prior to enrollment (Visit 0). If eligible, subjects will be seen in 9 additional scheduled visits.

At Visit 1/Enrollment (at least 7 days prior to the first dose), subjects will be enrolled and randomized to study product and to CVL time point, although no gel will be distributed at this visit. At this baseline visit, subjects will undergo a pelvic exam with 3 biopsies: 1 vaginal and 1 cervical biopsy for histology; and 1 cervical biopsy for transcriptome analysis (gene expression). Visit 1 will be scheduled to occur prior to the onset of menses (Menstrual Cycle Day 21-28 for women not on hormonal contraception; Hormonal Cycle day 15-22 for women on hormonal contraception) so that, ideally, the subject will have her menstrual period or withdrawal bleed after the biopsies and before she starts dosing.

At Visit 2 (Day 1), subjects will insert the first dose of their assigned study product in the clinic under direct observation, with a single PK assessment at 4 or 8 hours post-dose, at the same time they have the CVL collected; a swab for vaginal microbiome pre-dose; CVL collection pre-dose and 4h or 8h post-Dose 1 (based on randomization); and a baseline blood test for ADA. Visit 2 should be scheduled on Menstrual Cycle Day 7-10 for those not on hormonal contraception or Hormonal Cycle Day 1-4 for those on hormonal contraception, at least 7 days after Visit 1 to allow for sufficient time for healing, and to occur as soon as possible after menses

has ended. After Visit 2, subjects will continue using their product once daily for a total of 14 consecutive days.

At Visits 3, 4 and 5 (Days 3, 8 and 11), subjects will return to the clinic for outpatient blood draws to assess PK and safety. At those visits, subjects will insert their daily doses in the clinic after the blood draws. At Visit 4 (Day 8), a speculum exam will also be performed prior to insertion of the eighth dose.

At Visit 6 (Day 14), subjects will return to the clinic for the final dose of gel, PK sampling and ADA testing. After administering the final gel dose, subjects will remain in the clinic for up to 8 hours for PK blood draws.

At Visit 7 (Day 15), subjects will return to the clinic for a final (24 hour) PK blood draw and a pelvic exam with the same 3 biopsy specimens (2 cervical and 1 vaginal), CVL collection and a swab for vaginal microbiome.

At Visit 8 (Day 21), subjects will return for a final safety assessment including safety labs, pelvic exam, with a check to make sure biopsies have healed properly, an AE assessment, and a blood draw for ADA testing.

At Visit 9 (Day 28), the closing visit, subjects will have a final blood draw for ADA testing and AEs will be assessed.

During the study, subjects will be asked to record the date and time of each dose administered at home on labels affixed to plastic baggies. Subjects will be asked to bring all used and unused applicators with them to each clinic visit.

Assessment of vital signs and AEs will occur throughout the study. Subjects will refrain from non-study vaginal product use and vaginal activity, including intercourse, from 96 hours prior to enrollment (Visit 1) through Day 17.

Figure 2. STUDY SCHEMA – RANDOMIZED PERIOD

**Visit 0 Screening (≤45d before enrollment)**	**Begin abstinence 96h before enrollment**	**Visit 1 Enrollment/ Randomization**	**Visit 2 Day 1**	**Visit 3 Day 3**	**Visit 4 Day 8**	**Visit 5 Day 11**	**Visit 6 Day 14**	**Visit 7 Day 15**	**Visit 8 Day 21**	**Visit 9 Day 28**
		Baseline biopsies – at least 7d prior to Day 1/ Dose 1	14 x single daily doses →					Post-Dose 14 CVL, biopsies, micro-biome Final PK	Post-gel use safety visit ADA	Closing visit -- out-patient blood draw for ADA
			Dose 1 in clinic Single PK, ADA, CVL, Vaginal micro-biome	Dose 3 in clinic Single PK	Dose 8 in clinic Single PK	Dose 11 in clinic Single PK	Dose 14 in clinic Multiple PK, ADA			

7.6 Endpoints

Primary Endpoints:

1) Safety of PC-6500
   - Number and percent of subjects with TEAEs, SAEs, and TEAEs leading to premature discontinuation.
   - Number, percent and, in the investigator's judgment, medical significance of abnormalities in physical exams, pelvic exams, and biopsies, once product has been administered.
   - Number, percent and, in the investigator's judgment, medical significance of abnormalities in clinical laboratory parameters.

2) Pharmacokinetics (PK) of GRFT

Evaluation of concentrations of GRFT in blood during and after 1 day (the initial dose); then during and after 14 days of daily use of GRFT vaginal gel, with estimates of:

- Area under the time-concentration curve ($AUC_{0\text{-last}}$; $AUC_{0\text{-}\infty}$)
- Peak concentration (C_{max})
- Trough concentration (C_{min})
- Time to peak concentration (T_{max})
- Elimination half-life ($T_{½}$)
- Apparent clearance (Cl/F)

Exploratory Endpoints:

1) Post-treatment concentrations of GRFT and CG in CVLs.
2) Changes between baseline and after treatment in anti-HIV activity of CVLs in cell-based assays and mucosal explants.
3) Changes between baseline and after treatment in transcriptome analysis of gene expression in tissues and in concentrations of immune mediators in CVLs.
4) Changes between baseline and after treatment in the vaginal microbiome.
5) Changes between baseline and after treatment in ADA detection in blood.
6) Association between CVL drug levels with anti-HIV activity.
7) Changes between baseline and after treatment in anti-E. coli activity in CVLs.

7.7 Criteria for Proceeding to the Randomized Period (Main Study)

A review of TEAEs experienced and pelvic examination, physical examination, and laboratory findings will be carried out by the Council's Medical Monitor and Global Medical Safety Director together with the site Principal Investigator (PI) after the seventh subject has finished the OL safety period. If all seven subjects were able to tolerate the single dose, the randomized period will proceed.

7.8 Site

The study will be conducted at the Albert Einstein College of Medicine (Einstein) at the Clinical Research Center (CRC), a component of the Block Institute for Clinical and Translational Research (ICTR) at Einstein and Montefiore Medical Center (MMC). The CRC provides infrastructure for studies of normal and abnormal body function and for the investigation of the cause, progression, prevention, and treatment of human disease. Resources include specialized nursing staff, two outpatient facilities, and support for intensive studies involving adults and children. The latter are conducted in a procedure unit within the hospital designed for infusion studies, PK studies, and exercise experiments.

8. STUDY POPULATION

8.1 Selection of Study Population

The inclusion and exclusion criteria outlined below will be utilized to ensure the appropriate selection of study subjects. A total of 7 women will be enrolled in the OL period and a total of up to 20 women will be enrolled in the randomized period.

8.2 Recruitment

Einstein will employ a targeted recruitment strategy that includes contacting prior microbicide trial subjects who have indicated interest in future studies and given consent to be contacted, as well as advertising in local newspapers. Research Match will be utilized through the ICTR, and flyers will be distributed in the gynecology and medicine practices at MMC and in areas around the Einstein campus where women interested in research primarily work or are graduate students. All study specific recruitment materials and strategies, including advertising and internet-based materials will be reviewed and approved by the Population Council and Einstein IRBs prior to use, and this will be documented.

8.3 Inclusion Criteria

Female subjects must meet all of the criteria listed below to be eligible for inclusion in either study period, except as otherwise indicated:

1) Between 18 and 49 years of age (at screening), inclusive.
2) Willing and able to provide written informed consent.
3) Healthy, based on medical history, vital signs, physical examination, urinalysis, laboratory evaluations for genital infections and laboratory evaluations for hematology, liver and renal function.
4) HIV-negative, as determined by HIV ELISA test at screening.
5) In the absence of the use of exogenous hormone(s), have a self-reported regular menstrual cycle, defined as having a minimum of 21 days and a maximum of 35 days between menses.
6) Normal Pap test at screening or appropriately documented history of Pap test and completed follow-up of any abnormal Pap tests consistent with the American Congress of Obstetricians and Gynecologists (ACOG) practice bulletins #140 and #168. (Women

with atypical cells of undetermined significance [ASCUS] with no evidence of high-risk HPV can be included).

7) Agrees to use effective contraception for the duration of the trial. For the OL phase, subjects must be using a combined oral contraceptive pill or contraceptive patch.

   For the randomized phase, the following forms of contraception (per self-report) are permitted: same-sex relationship, male or female sterilization, combined oral contraceptives (OCs), contraceptive implant, contraceptive patch, intrauterine system (IUS), intrauterine device (IUD), depot medroxyprogesterone acetate (DMPA; Depo-Provera®).

   If hormonal contraception or IUD/IUS is being used, subjects must have been using the method for at least 30 days prior to screening, with no planned change in method during the study.

8) Willing to abstain from sexual intercourse/activity including receptive vaginal, oral, digital, and anal intercourse, and the use of any vaginal products including tampons, male and female condoms, contraceptive sponges, diaphragms, cervical caps, douches, lubricants, and vibrators/dildos; starting 48 hours prior to enrollment (Visit 1) through the final safety visit (Visit 3/Day 8) in the OL period; and starting 96 hours prior to enrollment (Visit 1) through Day 17 (3 days after the final gel dose) in the randomized period.

9) For the OL phase, must have an adult (≥18 years old) who can stay overnight with her until she attends the clinic for the 24h blood draw the following day (Day 2).

10) Agrees to not participate in any other clinical research for the duration of this trial.

8.4 Exclusion Criteria

Subjects who meet any of the following criteria will be excluded from the study:

1) History of or known sensitivity/allergy to any component of either study product.

2) Currently pregnant or breast-feeding, or within 3 months of last pregnancy outcome.

3) Participation in any other clinical research trial involving investigational or marketed products currently or within two months of participation prior to screening, including any trial of a spermicide, microbicide and/or drug.

4) Known bleeding disorder that could lead to prolonged or continuous bleeding with biopsy.

5) Diagnosed with or treated for any STI or pelvic inflammatory disease in the last 3 months. *Note: Women with a history of condylomata or genital herpes who have been asymptomatic for at least six months may be considered for eligibility.*

6) Positive test for *Neisseria gonorrhea* (NG), *Chlamydia trachomatis* (CT), or *Trichomonas vaginalis* (TV) at screening.

7) Symptomatic vulvovaginal candidiasis, bacterial vaginosis (BV), or urinary tract infection (UTI) at screening. **(Subjects who test positive at initial screening may be treated and re-tested once, and reconsidered for screening.)**
8) Presence of any clinically significant genital epithelial findings (e.g. abrasions, ulcerations, or lacerations, or vesicles) suspicious for STIs at screening.
9) History of hysterectomy or menopause.
10) Use of excluded contraceptive methods; for the OL period, all methods are excluded with the exception of a combined OC or contraceptive patch. For the randomized period, Nuvaring®, condoms (male or female), contraceptive sponge, diaphragm, or cervical cap are excluded.
11) History of gynecological surgery or procedure within the past two months.
12) History of uterine prolapse, undiagnosed vaginal bleeding or urethral obstruction within the last 3 months, including unexplained breakthrough bleeding requiring sanitary protection.
13) Known current drug abuse, including illicit drugs, or alcohol abuse.
14) Abnormal finding on laboratory or physical examination or a social or medical condition which, in the opinion of the investigator, would make participation in the study unsafe or would complicate interpretation of data.
15) Unable to comply with study requirements, including but not limited to, attending all study visits, using the gel as directed, observing abstinence as instructed, and use of allowable effective contraceptives.
16) History of latex allergy.

Women who participate in the OL period will be ineligible for the randomized period.

9. STUDY PRODUCT USE

9.1 Description of Study Products

**Active Investigational Gel:**	**Test:** PC-6500 (GRFT in CG gel)<br>4 g dosed once (safety run-in), or once daily for 14 days (main study)
**Placebo Gel:**	**Control:** PC-535 (CG gel)<br>4 g dosed once daily for 14 days (main study)

9.2 Justification, Selection, and Timing of Dose

A 2 mg dose achieved GRFT levels in macaque vaginal fluid that resulted in significant SHIV inhibition in a macaque vaginal explant model and in cell-based assays and resulted in a stable homogeneous formulation. The dose for this trial was based on the potential upper limit of an on-demand formulation, which is likely to be a fast-dissolve insert (FDI) releasing 2-4 mg. Given that a gel is not the target formulation, 4 mg GRFT (4 g PC-6500 gel) was considered to be sufficient for initial safety, PK and PD studies. The total dose administered in the trial will be no more than 1/10th of the NOAEL from the animal toxicology studies.

In the OL period, study clinicians will administer the single dose, consisting of one pre-filled vaginal applicator (HTI Plastics, Lincoln, NE) of PC-6500 gel. Subjects in the randomized period will be instructed to administer one dose of their assigned gel for 14 consecutive days, starting on Study Day 1. Five of the 14 daily doses (Doses 1, 3, 8, 11 and 14) will be administered in the clinic during scheduled visits, under direct clinical observation. These visits should be scheduled at approximately the same time each day to facilitate dosing around the same time as the initial dose (+/- 1 hour of the initial dose). Subjects will be asked to select a convenient time for their home doses that they will be most easily able to adhere to on a daily basis. Study Day 1 should be scheduled as soon as possible after menstruation ends to maximize the likelihood that all 14 days of dosing can occur during the non-bleeding days of the menstrual cycle.

9.3 Summary Table of Study Product Use

9.3.1 OL period

Visit	Study Day	Gel Dosing (Dose#)
Visit #0, Screening	Up to 30 days before enrollment	No gel use
Visit #1, Enrollment	1	SINGLE DOSE IN CLINIC
Visit #2, 24h PK	2	No gel, OUTPATIENT
Visit #3, Safety follow-up/Exit	8	No gel, OUTPATIENT

9.3.2 Randomized period

Visit	Study Day	Gel Dosing (Dose#)
Visit #0, Screening	Up to 45 days before enrollment	No gel use
Visit #1, Enrollment/Randomization	At least 7 days before first dose	No gel use
Visit #2 (1st dose)	1	#1 FIRST DOSE IN CLINIC
	2	#2 Home
Visit #3 (3rd dose)	3	#3 IN CLINIC
	4	#4 Home
	5	#5 Home
	6	#6 Home
	7	#7 Home
Visit #4 (8th dose)	8	#8 IN CLINIC
	9	#9 Home
	10	#10 Home
Visit #5 (11th dose)	11	#11 IN CLINIC
	12	#12 Home
	13	#13 Home
Visit #6 (final dose)	14	#14 IN CLINIC
Visit #7	15	No gel, OUTPATIENT
Visit #8 Safety follow-up	21	No gel, OUTPATIENT
Visit #9 Closing	28	No gel, OUTPATIENT

9.4 Method of Assigning Study Subjects to Study Intervention Groups

All subjects will be sequentially assigned to the next available unique identification (ID) number upon signing the informed consent form, prior to undergoing any screening procedures. For the single-dose OL period of the study, all subjects will receive PC-6500 (i.e. this period will not be blinded) and their ID number (PTID) will be their only ID number. For the main, randomized study period, each subject will receive a PTID at screening. At the outset of the trial, participants have been sequentially assigned to the next available randomization number after confirmation of eligibility at the enrollment visit (Visit 1). However, due to slower than anticipated accrual, it will not be feasible to enroll all 20 women. For the remaining participants who enroll, the Population Council Senior Statistician will assign the kit number and time point assignment, on a case by case basis, to balance the number of women assigned to product vs placebo; and to maintain the balance in post-dose specimen collection time points. Clinic staff will document the randomization number assignments which will determine which kit is given to the subject. The women in the main, randomized study period will be randomly assigned to either PC-6500 (n=up to 14) or PC-535 placebo gel (n=up to 6).

9.5 Randomization

A statistician who is not associated with the trial will create the randomization scheme which will pre-assign subjects to study intervention group. All subjects in the main study will also be stratified (1:1) for PK blood sampling and CVL collection to 4h (n=up to 10) or 8h (n=up to 10) post-Dose 1. Randomization occurred in blocks of ten (10). Each block of 10 was to include 3 placebo assignments and 7 active assignments; however, the final tally of active versus placebo will be unknown until the end of the study. Each block of 10 also includes 5 assignments to PK/CVL collection at 4h and 5 assignments to PK/CVL collection at 8h as indicated in the table below.

Intervention/Specimen Collection Time Stratification	Active	Placebo	Total
4h post-Dose 1	n=7	n=3	n=10
8h post-Dose 1	n=7	n=3	n=10
Total	n=14	n=6	N=20

Applicators will be packaged in kits which will be numbered 1-20 for the main study. The first subject randomized will receive applicators from kit number 1. The second subject will receive applicators from kit number 2, and so on. If a subject needs replacement applicators, the site will contact the Population Council team to be told which back-up applicator numbers should be given to the subject, based on the subject's randomization number. Study staff will receive with a list of kit numbers and specimen time point randomization assignments for all 20 participants. The randomization numbers will not identify PC-6500 or PC-535. The specimen time points will not be blinded – i.e. study staff will know which subjects will be scheduled for 4h and 8h time points when they receive the list from the Population Council.

9.6 Blinding

The two study gels (PC-6500 and PC-535) differ imperceptibly in appearance; both are translucent with a slight light beige color. Both study gels will be packaged identically. Each single-use applicator will be individually wrapped in a sealed package labeled with the name of the sponsor, protocol, and the contents of the package. For the OL period, all applicators will have information about PC-6500 only. For the randomized period, packages will indicate that the contents could be either PC-6500 or PC-535 placebo gel. The label will indicate that the product is limited to investigational use and is only for vaginal use. Population Council and study staff involved with the trial will remain blinded to the treatment assignment (placebo or active gel), however, the time point assigned for CVL specimen collection is not blinded.

9.7 Dispensing Study Product

Clinical drug supply will be provided by the Population Council to the clinical site pharmacy for dispensing. Study gels will be dispensed by the study pharmacist or designee upon receipt of a written prescription from an authorized prescriber. PC-6500 gel or PC-535 placebo gel will be dispensed in identical, pre-packaged, pre-filled, metered-dose single-use HTI applicators to a study staff member. In the OL period, one applicator (plus an extra, for back-up) will be issued when each subject is scheduled to enroll. In the randomized period, the pharmacy will dispense the number of applicators needed for insertion in the clinic and at home to a study staff member. The study staff member will then issue applicators, as needed, to the subjects for insertion in the clinic or at home. Subjects will be instructed to call or visit the clinic if any applicator is damaged or lost. Subjects will be given instructions on storing the product prior to use and retaining all used and unused applicators to return to the clinic at each study visit.

9.8 Concomitant Medications and Devices

9.8.1 Allowed

1) Any combined OC pill or patch (OL) or any hormonal contraception except NuvaRing® (randomized)
2) Vitamins and nutritional supplements (except mannose)
3) Anti-motility agents, oral antiemetic therapies
4) OTC analgesics and eye drops
5) Antihistamines

9.8.2 Prohibited

1) Any hormonal contraceptive other than combined OC pill or patch (OL); NuvaRing® (randomized)
2) Barrier contraceptives: condoms (male or female), sponge, diaphragm, cervical cap
3) Vaginal and rectal products (including, but not limited to vaginal medications, spermicidal products, lubricants, douches)
4) ARV therapy for prevention or treatment
5) HSV-2 treatments: Acyclovir, valacyclovir or other medication
6) Systemic steroids
7) Systemic antibiotics
8) Antihypertensive medications, including diuretics
9) Investigational therapies for any medical condition
10) Mannose (unless used as treatment for a severe reaction; See 15.4)

If a subject is taking any medication that is not included in the lists above, the Investigator should contact the Population Council Study Manager prior to enrollment.

9.9 Restrictions

Subjects are not permitted to participate in another clinical trial, including any trial of a spermicide, microbicide and/or drug, while participating in this study or in the two months prior to being screened. Subjects are required to abstain from sexual intercourse/activity, including receptive vaginal, oral, digital, and anal intercourse, and the use of any vaginal products including tampons, male and female condoms, contraceptive sponges, diaphragms, cervical caps, douches, lubricants, and vibrators/dildos starting from 48 hours before enrollment through Visit 3/Day 8 in the OL period, and from 96 hours before enrollment through Day 17 in the randomized period.

9.10 Study Product Exposure

In the OL period, a study nurse or clinician will administer the single dose of gel for each of the seven subjects. For the randomized period, subjects will self-administer the 1st, 3rd, 8th, 11th and 14th doses at the clinic, at approximately the same time each day, in the presence of a nurse/clinician. All other doses will be self-administered at home at approximately the same time each day. Subjects will be instructed to save all used applicators in individual zip-locked bags

provided by the study staff, and to return all applicators to the clinic at each visit. The number of applicators will be counted and retained for dye stain assay (DSA) testing. Exposure will be assessed based on the combined measure of in-clinic doses, as observed by the nurse/clinician, and the results of DSA testing on the returned opened applicators that were inserted at home.

9.11 Study Product Packaging and Labeling

Study Intervention:	PC-6500 gel (GRFT in a CG gel)
Dosage Form:	Topical gel formulation
Formulation:	0.1% w/w GRFT in CG gel
Dose:	Each vaginal applicator of PC-6500 delivers 4 mg of GRFT in 4 g of CG gel
Manufacturer:	Population Council, New York, New York
Description:	High-density polypropylene, pre-filled, metered-dose vaginal applicator with a screw top. The applicator will deliver a 4 g dose of PC-6500 gel when applied vaginally. The gel is off-white to light beige, clear to slightly turbid in appearance.
Storage Temp.:	-20°C (+/- 5° C) in pharmacy until distributed to clinic staff;<br>4 ° C (2-8° C) refrigerated in study clinic until distribution to subjects;<br>Room temperature (15°C to 30°C, or 59°F to 86°F) after distribution to study subjects (for up to 7 days)

Study Intervention:	PC-535 gel (CG placebo gel)
Dosage Form:	Topical gel formulation
Formulation:	3% carrageenan gel with identical formulation as PC-6500, excluding GRFT solution
Dose:	Each vaginal applicator of PC-535 delivers 4 g of gel
Manufacturer:	Population Council, New York, New York
Description:	High-density, polypropylene, pre-filled, metered-dose vaginal applicator with a screw top. The applicator will deliver 4 g of PC-535 gel when applied vaginally. The gel is off-white to light beige, clear to slightly turbid in appearance.
Storage Temp.:	-20°C (+/- 5° C) in pharmacy until distributed to clinic staff;<br>4 ° C (2-8° C) refrigerated in study clinic until distribution to subjects;<br>Room temperature (15°C to 30°C, or 59°F to 86°F) after distribution to study subjects (for up to 7 days)

Both gels will be packaged in identical overwraps with labeling that identifies the protocol number, sponsor, storage conditions, and investigational use warning as required by the FDA. In the OL period, gels will be labeled as PC-6500 investigational gel material. In the randomized period, study gels will be labeled as either PC-6500 or PC-535 placebo without indicating which specific gel is within. The contents will be specified via a randomized code known only to the sponsor/manufacturer. The subjects, study coordinator, laboratory staff, and statisticians will not be informed as to the product assignment of the subjects.

9.12 Study Product Storage and Accountability

The Investigator, or an approved designee (e.g., pharmacist, study nurse), will ensure that all study product is stored in a secured area, under the recommended storage condition, and in

accordance with applicable regulatory requirements. The Investigator or an approved designee must maintain adequate records documenting the receipt, use, loss or other disposition of the study product. The Investigator is responsible for ensuring the integrity of the study product prior to dispensing to a study subject. Any issues identified with the study product should be reported to the Council study team representative(s).

The study product will be accounted for in case report forms and drug accountability inventory forms, as instructed by the Council. The forms must identify the study product, with the batch or code numbers, and account for its disposition on a study subject-by-study subject basis, including specific dates and quantities. The forms must be signed by the individual (clinical site personnel) who dispensed the drug, and copies must be provided to the Council.

The clinical site will collect all applicators used at home from each study subject. All returned applicators, whether reported as used or unused will be counted; returned applicators reported as used will be tested using the DSA to confirm vaginal insertion. Unless otherwise authorized by the Council, all unopened study product applicators will be shipped back to the Population Council after product reconciliation by the study monitor. All used applicators – whether in the clinic or returned by the subject after home use – will be shipped to the Population Council as biohazardous material, in accordance with the study site's regulations. After DSA testing, the Population Council will destroy the used applicators in accordance with standard operating procedures (SOPs) for handling biohazardous materials.

10. STUDY PROCEDURES

10.1 Informed Consent Process

The informed consent form (ICF), which explains the protocol and requirements for screening and participation, will be reviewed with potential subjects by a member of the site's study team. During ICF review, the subject's competency will be assessed. Women who choose to be screened will be asked to sign the ICF. The ICF must be signed prior to conducting or performing any study-related procedure. The process of obtaining the subject's informed consent will be documented. The original signed ICF will be stored in the subject's study binder and a copy will be given to her.

10.2 Medical History

A complete medical history will be taken during the screening visit. The medical history will include any previous medical diagnoses, surgical procedures of major organ systems, social and gynecologic history, as well as current medical conditions. The gynecologic history will include previous and current gynecologic surgeries, conditions, infections of the female genital tract, pregnancies, miscarriages, fetal losses including spontaneous or induced abortions, and treatment of any condition.

10.3 Medication History

The medication history is to be taken at screening and will include: generic name, route, regimen and indication of all prescription, OTC, herbal, home-made therapies, vitamins and nutritional supplements taken in the 30 days prior to screening. Any changes or additions to medications occurring during the study period will be documented on a concomitant medications CRF.

10.4 Documentation of HIV Status

HIV testing by any FDA-approved licensed ELISA or EIA, along with a FDA-approved confirmatory test, if indicated, will be performed at screening to document HIV status.

10.5 Physical Examination

A complete physical examination will be performed and documented during Screening (Visit 0) and at the final safety visit (Visit 3, OL; Visit 8, Randomized). At any other visit, a targeted physical examination will be conducted if clinically indicated.

Vital signs will be assessed at every visit. Height will only be assessed at screening. Weight will only be assessed at screening and at the final safety visit (Visit 3, OL; Visit 8, Randomized).

10.6 Pelvic Examination

At screening (both study periods), enrollment, before the first dose (both periods), before the eighth dose (randomized only), and after dosing concludes (both periods), a pelvic examination will be conducted according to the Photo Atlas for Microbicide Evaluation (Bollen et al. 2002) to assess the vulva, perianal area, and, with a naked eye speculum exam to visualize the vagina and cervix. A bimanual exam (with lubricant, as necessary) is to be conducted at screening and at the final safety visit to evaluate the uterus and adnexae; and a breast exam is to be conducted at screening. A pelvic exam will only be conducted at other visits during dosing if a subject reports symptoms. At specified visits, pelvic exams will include tests for RTIs/STIs and collection of vaginal swabs, biopsy and CVL specimens. In addition, although subjects are instructed to remain sexually abstinent during the study, rapid stain identification of human semen (RSID) testing will be performed at designated visits because the presence of semen (from unprotected intercourse) could have an impact on PD evaluations. No subjects will be withdrawn from the study due to positive RSID tests, however, specimens may be excluded from some final exploratory endpoint analyses.

10.7 Biopsies for Histology (randomized period only)

In the main study, at Visit 1 (the enrollment visit—at least 7 days prior to administration of the first dose of study gel) and again at Visit 7 (Day 15), 1 vaginal biopsy specimen and 1 cervical biopsy specimen (3mm x 5mm each) will be collected to evaluate possible histological changes associated with the study treatment. Local anesthesia will be offered prior to vaginal biopsy collection. Biopsy specimens will be sent to a pathologist for evaluation.

10.8 Clinical Laboratory Tests

The following specimens will be collected and evaluated at the MMC laboratory, unless otherwise indicated:

**Hematology:**	**Serum Chemistry:**
• Hematocrit (Hct)	• Albumin (ALB)
• Hemoglobin (Hgb)	• Alkaline phosphatase (ALK-P)
• Platelet count	• Alanine aminotransferase (ALT; SGPT)
• Red blood cell (RBC) count	• Aspartate aminotransferase (AST; SGOT)
• White blood cell (WBC) count with differential	• Blood urea nitrogen (BUN)

HIV, HSV-1 and HSV-2
Coagulation:

- Prothrombin time (PT)
- Activated partial thromboplastin time (PTT)

***Dipstick Urinalysis** (CRC)**:**

- Specific gravity
- pH
- Urine color
- Appearance
- WBC esterase
- Protein
- Glucose
- Ketones
- Occult blood
- Bilirubin
- Urobilinogen
- Nitrite

Pregnancy:
Urine β-hCG (CRC)

- Calcium (Ca^{2+})
- Chloride (Cl^-)
- Creatinine
- Gamma-glutamyl transferase (GGT)
- Glucose
- Lactate dehydrogenase (LDH)
- Phosphorus
- Potassium (K^+)
- Sodium (Na^+)
- Total bilirubin
- Direct bilirubin
- Total protein
- Uric acid

Vaginal/cervical:

- Endocervical swabs for Nucleic Acid Amplification Test (NAAT) for NG, CT, TV
- Pap smear
- pH strips for vaginal pH (CRC)
- Commercially available supplies for Wet Mount (vaginal smear) (CRC)

***If dipstick urinalysis indicates abnormal results, urine specimen will be sent to MMC laboratory for complete urinalysis and, if indicated, urine culture.

10.9 Specimens for Research Purposes

The following specimens will be collected, processed, stored and shipped as outlined in the Specimen Collection Manual (SCM).

- Swab for vaginal microbiome
- Cervicovaginal lavages (CVL) for:
  - GRFT and CG concentrations
  - Immune mediators
  - PD (cell-based assays and mucosal explants)
  - anti-*E. coli* activity
  - Rapid identification of human semen (RSID)
- Tissue (biopsy) for:
  - Transcriptome analysis of gene expression
- Serum for ADA

10.9.1 Tissues: Transcriptome analysis

In the randomized period, cervical tissue biopsies will be collected to assess changes in transcriptome (RNA seq). One cervical biopsy (3mm x 5mm) will be collected at Visit 1/baseline (enrollment, before dosing) and 1 cervical biopsy will be collected after the 14th dose at Visit 7 (Day 15).

10.9.2 CVL and swab: drug concentrations, PD, immune mediators, anti-*E. coli* activity, and vaginal microbiome

CVLs will be collected to determine (1) GRFT and CG concentrations; (2) anti-HIV activity in cell-based assays and mucosal explants; (3) concentrations of genital tract immune mediators; (4) anti-*E. coli* activity; and (5) RSID testing to determine if condom-less sex occurred (NOTE: in the randomized portion of the study, the first RSID test will be done at enrollment using a swab instead of CVL). Each subject in the OL period will have 1 CVL collected at baseline (enrollment, before dosing) and 24h post-dose. Each subject in the randomized period will have 1 CVL collected at Visit 2/Day 1 before the first dose, after the first dose (at 4 or 8h, depending on stratification) and after the 14th dose (Day 15). Vaginal swabs will be collected from all subjects at before the first dose and following the last dose (Day 2 in the OL period; Day 15 in the randomized period) to determine if there are changes in the vaginal microbiome after GRFT administration.

10.9.3 ADA measurements (randomized period only)

Serum will be collected before dosing (Visit 2), Day 14 (Visit 6), Day 21 (Visit 8) and Day 28 (Visit 9) to measure systemic concentrations of ADA.

11. STUDY EVALUATIONS: Open-Label Period

Please refer to Appendix #1 for Schedule of Visits and Procedures (OL period)

The OL period will involve seven (7) women receiving one single 4 g dose of PC-6500 inserted in the clinic by a study nurse/clinician. Safety will be assessed before and after the dose, as well as on Day 8/Visit 3 (one week after dosing). Subjects will remain in the clinic for 12 hours after the dose for PK assessments. They will then return to the clinic 24 hours after dosing for a final PK blood draw (Visit 2/Day 2). Subjects will be exited on Day 8 (Visit 3) after a final safety assessment. Specific procedures to be conducted at each visit are outlined below and in Appendix 1. Further details are available in the Study Specific Procedures (SSP) manual.

Visits 1 and 2 must be scheduled to occur on the specified days in the protocol or will be considered a protocol deviation. Visit 3 should be scheduled on Day 8, 7 days after the single dose (+/- 24 hours).

All PK blood draws must be taken exactly as scheduled, plus/minus 5 minutes up through the 4-hour blood draw, and plus/minus 15 minutes for blood draws thereafter. The exact time of each PK blood draw must be documented and any deviations reported.

11.1 OL Safety Period, Visit 0: Screening Visit (≤30 days before Enrollment)

The Screening Visit may take place up to 30 days prior to Visit 1/Enrollment. If more than 30 days elapse between Visit 0 (Screening) and Visit 1, screening procedures must be repeated.

OL Safety Period<br>Screening Visit<br>(Visit 0)<br>(≤30 days before Enrollment)		
Component		Procedures
Administrative/<br>Regulatory		• Explain study procedures, including need for abstinence, and specific procedures to be performed at this visit<br>• Obtain written informed consent<br>• Assign PTID number<br>• Record locator information<br>• Record demographics<br>• Assess eligibility (partial; completed after lab test results received)<br>• Provide reimbursement for study visit<br>• Schedule next study visit, if applicable
Clinical		• Take medical history<br>• Record medication history/concomitant medications<br>• Assess vital signs, height and weight<br>• Perform complete physical exam<br>• Obtain urine sample(s)<br>• Obtain blood samples<br>• Perform pelvic exam (after confirmation of negative pregnancy test) with bimanual exam and breast exam<br>• Collect pelvic samples for STI testing (and Pap smear[1], if needed)<br>• Treat or prescribe treatment for symptomatic UTI, vaginal candidiasis, and BV; subject may be rescreened after completing treatment<br>• Refer for other findings, as indicated; women found to be pregnant, HIV positive, or with any other exclusionary abnormalities at this visit will end participation here
Behavioral		• HIV risk reduction and pre-/post-HIV test counseling<br>• Protocol adherence counseling to reinforce partnership in research and responsibilities (abstinence, contraception, etc.)
Laboratory	Urine	• β-hCG<br>• Dipstick urinalysis (UA)<br>• Urine culture, if indicated
	Blood	• HIV-1 test, HSV-1, HSV-2<br>• Hematology, Chemistry and Coagulation (see Section 10.8)

[1] If no appropriately documented history of Pap test and completed follow-up of any abnormal Pap tests consistent with ACOG bulletins #140 and #157.

OL Safety Period<br>Screening Visit<br>(Visit 0)<br>(≤30 days before Enrollment)		
Component		Procedures
	Pelvic	• Cervical swab for NAAT for NG, CT and TV<br>• Pap smear, if needed[1]<br>• Vaginal pH and fluid for wet mount microscopy (saline for BV and KOH for vulvovaginal candidiasis), if indicated

If a subject tests positive for HIV or other RTI/STI, she will meet with the site clinician to discuss the results. Subjects will be provided with their HIV test results in the context of post-test counseling. Subjects found to be HIV infected will be referred to available sources of medical and psychosocial care and support, and local research studies for HIV-infected adults. Subjects who test positive for other STIs will be offered treatment and referral for appropriate follow-up and will not be eligible for the trial. Subjects who test positive for other RTIs (BV, candida) or UTIs will be offered treatment and may be rescreened.

11.2 OL Safety Period, Visit 1: Enrollment/Dosing (Day 1)

Enrollment/Visit 1 should occur as early as possible in the subject's menstrual cycle after menses ends. If more than 30 days elapse between Visit 0/Screening and Visit 1/Enrollment, screening procedures must be repeated. No one may be screened more than twice.

Prior to scheduling Visit 1, the Eligibility Criteria (Sections 8.3 and 8.4) should be reviewed to ensure the subject meets all criteria. Visit 1 is the subject enrollment visit and the sole day of study product use.

<table>
<tr><td colspan="3">OL Safety Period
Visit 1
(Study Day 1)
PRE-Dose #1</td></tr>
<tr><td colspan="2">Component</td><td>Procedures</td></tr>
<tr><td colspan="2">Administrative/ Regulatory</td><td>• Confirm eligibility
• Record/confirm locator information
• Review procedures to be performed at this visit</td></tr>
<tr><td colspan="2">Clinical</td><td>• Update concomitant medications
• Assess vital signs
• Provide available test results; if required, treat or prescribe treatment for exclusionary STIs (NG, CT, TV) and discontinue subject;
• Treat or prescribe treatment for symptomatic UTI, vaginal candidiasis, and BV; subject may be rescreened after completing treatment
• Conduct targeted physical exam, if clinically indicated
• Perform EKG
• Obtain relevant urine, blood, and pelvic samples before dose
• Perform pelvic exam after confirmation of negative pregnancy test, before dose
• Collect vaginal swab for microbiome assessment
• Collect baseline CVL for drug concentration, PD, E. coli and RSID testing
• Administer single 4 g vaginal dose of PC-6500; record exact time (hour/minutes) dose inserted.</td></tr>
<tr><td rowspan="3">Laboratory</td><td>Urine</td><td>• β-hCG
• Dipstick UA, if indicated
• Urine culture, if indicated</td></tr>
<tr><td>Blood</td><td>• Baseline PK prior to dosing – record exact time (hours/minutes) of pre-dose blood draw
• Pre-dose Hematology, Chemistry and Coagulation (see Section 10.8)</td></tr>
<tr><td>Pelvic</td><td>• CVL for API concentrations, PD, E. coli and RSID
• Vaginal microbiome
• pH, if clinically indicated
• Wet mount, if clinically indicated</td></tr>
<tr><td colspan="3">SINGLE DOSE ADMINISTERED BY CLINICIAN TO SUBJECT IN CLINIC</td></tr>
</table>

OL Safety Period<br>Visit 1<br>(Study Day 1)<br>POST-Dose #1		
Component		Procedure
Administrative		• Confirm subject has someone to stay with her overnight<br>• Instruct subject to call or text site when she returns home<br>• Provide reimbursement for study visit<br>• Schedule next study visit
Clinical		• Collect used applicator for positive control for DSA testing<br>• Assess AEs<br>• Assess vital signs periodically<br>• Conduct targeted physical exam, if clinically indicated<br>• Conduct pelvic exam, if clinically indicated<br>• Collect vaginal swabs, if clinically indicated<br>• Obtain blood samples for PK at 0.5,1,2,3,4h post-dose (+/- 5 minutes), and 6,8,10,12h post-dose (+/- 15 minutes)<br>• Obtain post-dose blood sample for safety labs as late as possible prior to discharging subject<br>• Obtain urine samples, if clinically indicated
Laboratory	Urine	• Dipstick UA, if indicated<br>• Urine culture, if indicated
	Blood	• Hematology, Chemistry, and Coagulation testing (See Section 10.8)<br>• PK assessments at 0.5,1,2,3,4h post-dose; (+/-5min),6,8,10,12 h post-dose (+/-15 minutes)
	Pelvic	• pH, if indicated<br>• Wet mount, if indicated

Subjects will be instructed to call the site to report any AE experienced during the study.

11.3 OL Safety Period, Visit 2: 24-Hour PK Visit (Day 2)

<table>
<tr><th colspan="3">OL Safety Period
Visit 2
(Study Day 2)</th></tr>
<tr><td colspan="2">Component</td><td>Procedure</td></tr>
<tr><td colspan="2">Administrative/ Regulatory</td><td>• Record/confirm locator information
• Review procedures to be performed at this visit
• Schedule next visit
• Provide compensation</td></tr>
<tr><td colspan="2">Clinical</td><td>• Update concomitant medications
• Assess vital signs
• Perform EKG
• Obtain blood sample for 24h PK and safety labs
• Collect vaginal swab specimen for microbiome assessment
• Collect CVL 24h post-dose for API concentration, PD, E. coli and RSID
• Assess AEs
• Conduct targeted physical exam, if indicated
• Conduct pelvic exam, if indicated
• Collect vaginal swabs, if indicated
• Obtain urine sample, if indicated</td></tr>
<tr><td rowspan="3">Laboratory</td><td>Urine</td><td>• Dipstick UA, only if indicated
• Urine culture, only if indicated</td></tr>
<tr><td>Blood</td><td>• Hematology, chemistry, and coagulation testing (see Section 10.8)
• 24h PK assessment</td></tr>
<tr><td>Pelvic</td><td>• 24h CVL for API concentration, PD, E. coli, RSID
• Vaginal microbiome
• pH, if indicated
• Wet mount, if indicated</td></tr>
</table>

Subjects will be instructed to call the site to report any AE experienced during the study.

11.4 OL Safety Period, Visit 3: Safety Follow-Up/Closing (Day 8)

This visit occurs on Day 8 of the study (7 days after the single dose):

OL Safety Period<br>Visit 3<br>(Study Day 8)		
Component		Procedure
Administrative/ Regulatory		• Record/confirm locator information<br>• Review procedures to be performed at this visit<br>• Provide compensation
Clinical		• Update concomitant medications<br>• Assess vital signs and weight<br>• Conduct complete physical exam<br>• Obtain blood sample<br>• Obtain urine sample<br>• Perform pelvic exam with bimanual exam<br>• Collect vaginal/cervical swabs, only if indicated<br>• Assess AEs
Laboratory	Urine	• β-hCG<br>• Dipstick UA<br>• Urine culture, only if indicated
	Blood	• Hematology, Chemistry and Coagulation testing (see Section 10.8)
	Pelvic	• pH, only if indicated<br>• Wet mount, only if indicated

12. STUDY EVALUATIONS: Randomized Period

Please refer to Appendix #2 for Schedule of Visits and Procedures (Randomized Period).

The randomized period will commence after a review of data from the OL safety period. Subjects in the randomized, double-blind, placebo-controlled portion of the trial will be assigned to PC-6500 or PC-535 placebo gel. Subjects will be instructed to insert 1 dose of gel per day for 14 consecutive days. The first, 3rd, 8th, 11th and 14th doses will be self-administered in the clinic in the presence of a study nurse/clinician at approximately the same time (+/- 30 minutes). The remaining doses will be self-administered at home at around the same time each day.

Pelvic exams will occur at Visit 0 (Screening), Visit 1 (Enrollment), Visits 2 (Day 1) and 4 (Day 8, prior to dosing), Visit 7 (Day 15), Visit 8 (Day 21) and at other times if a subject reports symptoms. At enrollment, 1 vaginal and 2 cervical biopsy specimens will be collected; prior to collection of biopsies, a swab will be collected for RSID testing. A naked eye speculum exam will be conducted at Visit 2/Day 1 prior to administration of Dose 1 to ensure that the biopsies have healed. Subjects will be encouraged to call or visit the clinic at any time between scheduled visits if they experience any AEs, require additional gel supply, or have any questions.

All follow-up visits must occur within 1 day of the scheduled visit (+/- 24 hours). Visit 0/Screening can occur up to 45 days prior to Visit 1/Enrollment. Visit 1/Enrollment must be scheduled when the subject is not on menses, and at least 7 days before Visit 2/Day 1 to allow for sufficient time for the biopsy sites to heal. The final safety visit (Visit 8/Day 21) can occur up to 24h prior to Day 21 and up to 3 days AFTER Day 21 (i.e. Day 24). The Visit 9 final ADA blood draw must occur within 1 day of Day 28 (+/- 24 hours).

12.1 Randomized Period, Visit 0: Screening Visit (≤45 Days before Visit 1/Enrollment)

Visit 0/Screening may occur up to 45 days prior to Visit 1/Enrollment. If more than 45 days elapse between the Screening Visit and Visit 1, screening procedures must be repeated. No one may be screened more than twice.

Randomized Period<br>Screening Visit<br>(≤45 Days before Visit 1/Enrollment)	
Component	• Procedure
Administrative/<br>Regulatory	• Explain study procedures, including need for abstinence, and procedures to be performed at this visit<br>• Obtain written informed consent<br>• Assign PTID number<br>• Collect/record locator information<br>• Collect demographic data<br>• Assess eligibility (partial)<br>• Provide reimbursement for study visit<br>• Schedule next study visit, if applicable
Clinical	• Take medical history;<br>• Document start of last menstrual period (LMP)<br>• Record medication history/concomitant medications<br>• Assess vital signs, height and weight<br>• Perform complete physical exam<br>• Obtain urine sample<br>• Obtain blood samples<br>• Perform pelvic exam (after confirmation of negative pregnancy test) with bimanual exam and breast exam<br>• Collect pelvic samples for STI testing (and Pap smear, if needed)<br>• Treat or prescribe treatment for symptomatic UTI, vaginal candidiasis, and BV; subject may be rescreened after completing treatment<br>• Refer for other findings, as indicated; women who are found pregnant, HIV positive, test positive for STIs or with any other abnormalities at this visit will end participation here

Randomized Period<br>Screening Visit<br>(≤45 Days before Visit 1/Enrollment)		
Component		• Procedure
Behavioral/Counseling		• HIV risk-reduction and pre-/post-HIV test counseling<br>• Protocol adherence counseling to reinforce partnership in research and responsibilities (abstinence, contraception, etc.); **REMIND SUBJECT TO ABSTAIN FROM VAGINAL PRODUCT USE OR SEXUAL ACTIVITY FOR 96 HOURS PRIOR TO ENROLLMENT VISIT**
Laboratory	Urine	• β-hCG<br>• Dipstick UA<br>• Urine culture, only if indicated
	Blood	• HIV-1 test, HSV-1, HSV-2<br>• Hematology, Chemistry, Coagulation (see Section 10.8)
	Pelvic	• Cervical swab for NAAT for NG, CT and TV<br>• Pap smear, if indicated[2]<br>• Vaginal pH and fluid for wet mount microscopy (saline for BV and KOH for vulvovaginal candidiasis), if indicated

12.2 Randomized Period, Visit 1: Enrollment/Baseline (≥ 7 days before Visit 2/Day 1)

Visit 1 of the Randomized Period should occur during the non-bleeding portion of the subject's menstrual cycle to ensure a clear field to obtain the biopsy specimens. Visit 1 should be scheduled for at least 7 days prior to Visit 2/Day 1 and within 45 days of Visit 0/Screening. If more than 45 days elapse between Visit 0/Screening and Visit 1, screening procedures must be repeated. Prior to scheduling Visit 1, the Eligibility Criteria (Sections 8.3 and 8.4) should be reviewed to ensure subject meets all criteria.

Randomized Period<br>Visit 1: Enrollment<br>(≥7 Days before Visit 2/Day 1)	
Component	Procedure
Administrative/<br>Regulatory	• Confirm eligibility<br>• Confirm/record locator information<br>• If eligible, assign randomization number<br>• Follow procedures for randomization assignment<br>• Review procedures to be performed at this visit<br>• Provide reimbursement for this visit
Behavioral/<br>Counseling	• Protocol adherence and contraceptive counseling<br>• Risk reduction counseling, if required, based on STI test results

[2] If no appropriately documented history of Pap test and completed follow-up of any abnormal Pap tests consistent with ACOG bulletins #140 and #157.

Randomized Period<br>Visit 1: Enrollment<br>(≥7 Days before Visit 2/Day 1)		
Component		Procedure
Clinical		• Provide available test results<br>• Treat or prescribe treatment for symptomatic UTI, vaginal candidiasis, and BV; subject can be re-screened once after completing treatment<br>• Treat or prescribe treatment for exclusionary STIs (NG, CT, TV) and discontinue subject<br>• Update concomitant medications<br>• Assess AEs<br>• Assess vital signs<br>• Conduct targeted physical exam, if clinically indicated<br>• Obtain urine sample before pelvic exam<br>• Confirm negative pregnancy test; women with positive pregnancy tests are ineligible and will be discontinued<br>• Perform pelvic exam; if any conditions are detected requiring vaginal swab collection and/or treatment, subject is ineligible and will need to be treated and rescreened; if no abnormalities detected, woman can be enrolled<br>• Collect vaginal swab for RSID testing<br>• Collect biopsy specimens (1 vaginal, 2 cervical)<br>• Assign randomization number/follow randomization procedures
Laboratory	Urine	• β-hCG<br>• Dipstick UA, only if clinically indicated<br>• Urine culture, only if indicated
	Pelvic	• Swab for RSID test<br>• Biopsies (pathology and exploratory)<br>• Vaginal pH, if indicated<br>• Wet mount, if indicated

12.3 Randomized Period, Visit 2: Dose #1 (Day 1)

Visit 2 should occur as early as possible in the subject's menstrual cycle after menses ends. Visit 2 must be scheduled at least 7 days after Visit 1 (Enrollment) to allow sufficient time for biopsy healing. Visit 2 is the first day of study product use. Subjects will insert their first dose under clinical supervision.

Randomized Period<br>Visit 2 (Day 1): Begin dosing<br>PRE-Dose Procedures	
Component	Procedure
Administrative/<br>Regulatory	• Confirm/record locator information<br>• Review procedures to be performed at this visit

Randomized Period<br>Visit 2 (Day 1): Begin dosing<br>PRE-Dose Procedures		
Component		Procedure
Clinical		• Update concomitant medications<br>• Assess AEs<br>• Assess vital signs<br>• Document start of last menstrual period (LMP)<br>• Conduct targeted physical exam, if clinically indicated<br>• Obtain urine sample before 1st dose; subjects with a positive pregnancy test are ineligible and will be discontinued<br>• Obtain pre-dose blood sample for PK, safety labs and ADA testing<br>• Perform pelvic exam (with baseline CVL for API concentration, PD, immune mediators, E. coli, RSID) after confirmation of negative pregnancy test, before first dose; if any conditions are detected requiring vaginal swab collection and/or treatment, subject is ineligible and will be discontinued<br>• Collect swab for vaginal microbiome assessment<br>• Instruct subject to self-administer her first dose under supervision of study clinical personnel AFTER pre-dose sampling; record exact time dose is inserted (hour, minute)
Laboratory	Urine	• β-hCG<br>• Dipstick UA, only if indicated<br>• Urine culture, only if indicated
	Blood	• Pre-dose hematology, chemistry and coagulation (see Section 10.8)<br>• Pre-dose PK sample before 1st dose<br>• Pre-dose ADA testing
	Pelvic	• Vaginal microbiome<br>• CVL (API concentrations, PD, immune mediators, E. coli, RSID)<br>• Vaginal pH, if indicated<br>• Wet mount, if indicated
DOSE #1 SELF-ADMINISTERED IN CLINIC		

Randomized Period<br>Visit 2 (Day 1): Begin dosing<br>POST-Dose Procedures		
Component		Procedure
Administrative/ Regulatory		• Schedule next study visit<br>• Provide reimbursement for visit
Clinical		• Collect used applicator from subject for positive control for DSA testing<br>• Assess AEs<br>• Assess vital signs periodically<br>• Conduct targeted physical exam, if clinically indicated<br>• Obtain single PK blood sample at 4h or 8 hours (+/- 5 minutes) OR 8h (+/- 15 minutes) post-dose, based on randomization<br>• Collect CVL (API concentrations, PD, immune mediators, E. coli, RSID) at 4 or 8h post-dose (+/- 15 minutes), based on randomization<br>• Conduct pelvic exam ONLY if indicated<br>• Collect vaginal/cervical swabs, only if indicated<br>• Dispense applicators and instruct subject on home administration<br>• Dispense pre-labeled plastic bags for collection of used applicators<br>• Instruct subject on labeling and storage procedures for used applicators
Behavioral/ Counseling		• Counseling on adherence, including review of abstinence requirements and reinforcing partnership in research
Laboratory	Urine	• Dipstick UA, only if clinically indicated<br>• Urine culture, only if clinically indicated
	Blood	• Post-dose PK samples at 4 OR 8h post-dose
	Pelvic	• CVL specimen (API concentrations, PD, immune mediators, E. coli, RSID)<br>• Vaginal pH, if indicated<br>• Wet mount, if indicated

Subjects will be instructed to call the site to report any AE experienced during follow-up.

12.4 Randomized Period, Visits 3-5: Doses 3, 8, 11 (Days 3, 8 and 11)

Visits 3, 4 and 5 are short outpatient visits with a single PK blood draw, safety labs, in-clinic dosing, and gel resupply. Visit 4 also includes a pelvic exam and pregnancy test.

<table>
<tr><td colspan="3">Randomized Period
Visits 3, 4 and 5 (Days 3, 8 and 11)
PRE-Dose Procedures</td></tr>
<tr><td colspan="2">Component</td><td>Procedure</td></tr>
<tr><td colspan="2">Administrative/ Regulatory</td><td>• Confirm/record locator information
• Review procedures to be performed at this visit</td></tr>
<tr><td colspan="2">Clinical</td><td>• Collect and document returned applicators for DSA testing
• Update concomitant medications
• Assess vital signs
• Obtain urine for pregnancy test (VISIT 4/DAY 8 ONLY)
• Conduct targeted physical exam, if clinically indicated
• Perform pelvic exam at VISIT 4/DAY 8 ONLY (and at other visits, only if indicated; with relevant vaginal/cervical swabs)
• Obtain blood for PK/safety assessments
• Assess AEs
• Instruct subject to self-administer study product under supervision of study clinical personnel AFTER pre-dose sampling at the same time Dose 1 was inserted (+/- 30 minutes)</td></tr>
<tr><td rowspan="3">Laboratory</td><td>Blood</td><td>• Pre-dose PK sample
• Hematology, chemistry, and coagulation</td></tr>
<tr><td>Urine</td><td>• β-hCG (VISIT 4/DAY 8 ONLY)
• Dipstick UA, only if indicated
• Urine culture, only if indicated</td></tr>
<tr><td>Pelvic</td><td>• Vaginal pH, if indicated
• Wet mount, if indicated</td></tr>
<tr><td colspan="3">SELF-ADMINISTER DOSE (#s 3, 8 and 11) IN CLINIC</td></tr>
</table>

Randomized Period<br>Visits 3, 4 and 5 (Days 3, 8 and 11)<br>POST-Dose Procedures		
Component		Procedure
Administrative/ Regulatory		• Schedule next study visit<br>• Provide reimbursement for visit
Clinical		• Assess vital signs<br>• Conduct targeted physical exam, if clinically indicated<br>• Perform pelvic exam, if indicated<br>• Collect vaginal/cervical swabs, if indicated<br>• Assess AEs<br>• Dispense applicators for home use<br>• Dispense pre-labeled plastic bags for saving used applicators
Behavioral/Counseling		• Protocol/adherence counseling
Laboratory	Blood	• Safety labs, only if indicated
	Urine	• Dipstick UA, only if indicated<br>• Urine culture, only if indicated
	Pelvic	• Vaginal pH, if indicated<br>• Wet mount, if indicated

Subjects will be instructed to call the site to report any AE experienced during the follow-up period.

12.5 Randomized Period, Visit 6: Final Dose (Day 14)

Visit 6 (Day 14) will last up to 10 hours and involves the final day of study product use, administered in the clinic, with PK assessments.

Randomized Period<br>Visit 6: Final study product use<br>(Study Day 14)<br>Pre-Dose Procedures	
Component	Procedure
Administrative/ Regulatory	• Confirm/record locator information<br>• Review procedures to be performed at this visit

<table>
<tr><td colspan="2">Clinical</td><td>Collect and document number of returned applicators for DSA testing
Update concomitant medications
Assess AEs
Assess vital signs
Conduct targeted physical exam, if clinically indicated
Obtain urine, blood, and pelvic samples before dosing
Perform pelvic exam, only if subject reports significant irritation
Collect vaginal/cervical swabs, only if indicated
Instruct subject to self-administer final dose of study product under supervision of study clinical personnel AFTER pre-dose sampling at the same time that Doses 1 and 8 were inserted (+/- 30 minutes)</td></tr>
<tr><td rowspan="3">Laboratory</td><td>Urine</td><td>β-hCG
Dipstick UA, only if indicated
Urine culture, only if indicated</td></tr>
<tr><td>Blood</td><td>Pre-dose PK sample
Pre-dose sample for ADA testing
Hematology, chemistry, and coagulation (see Section 10.8)</td></tr>
<tr><td>Pelvic</td><td>Vaginal pH, if indicated
Wet mount, if indicated</td></tr>
<tr><td colspan="3">DOSE #14 SELF-ADMINISTERED IN CLINIC</td></tr>
<tr><td colspan="3">POST-Dose Procedures</td></tr>
<tr><td colspan="2">Component</td><td>Procedure</td></tr>
<tr><td colspan="2">Administrative/ Regulatory</td><td>Schedule next study visit
Provide reimbursement for visit</td></tr>
<tr><td colspan="2">Clinical</td><td>Collect used applicator from subject for DSA testing
Assess AEs
Assess vital signs
Conduct targeted physical exam, if clinically indicated
Collect blood specimens for PK at 0.5,1,2,4h post-dose (+/- 5 min); and 6, 8h post-dose (+/- 15 min)</td></tr>
<tr><td rowspan="3">Laboratory</td><td>Urine</td><td>Dipstick UA, only if indicated</td></tr>
<tr><td>Blood</td><td>Post-dose PK sampling at 0.5, 1, 2, 4, 6, 8h post-dose 14</td></tr>
<tr><td>Pelvic</td><td>Vaginal pH, if indicated
Wet mount, if indicated</td></tr>
</table>

Subjects will be instructed to call the site to report any AE experienced during the follow-up period.

12.6 Randomized Period, Visit 7: Final PK Assessment (Day 15)

Visit 7 involves the final PK assessment and biopsy collection and will last 1-2 hours.

Randomized Period<br>Study Visit 7: Final PK assessment<br>(Study Day 15)		
Component		Procedure
Administrative/ Regulatory		• Confirm/update locator information<br>• Review procedures for visit<br>• Schedule next study visit<br>• Provide reimbursement for visit
Clinical		• Update concomitant medications<br>• Assess AEs<br>• Assess vital signs<br>• Conduct targeted physical exam, if clinically indicated<br>• Collect blood specimens for 24-hour post-dose PK (+/- 15 minutes) and for safety labs<br>• Perform pelvic exam<br>• Collect vaginal swab specimen for microbiome assessment<br>• Collect CVL (API concentrations, PD, immune mediators, E. coli, RSID)<br>• Collect biopsy specimens (1 vaginal, 2 cervical)<br>• Collect vaginal swabs, if indicated
Laboratory	Urine	• Dipstick UA, only if indicated<br>• Urine culture, only if indicated
	Blood	• Post-dose hematology, chemistry, coagulation (see Section 10.8)<br>• 24-hour PK sample post-Dose 14
	Pelvic	• Post-dose CVL for API concentrations, PD, immune mediators, E. coli, RSID<br>• Post-dose vaginal and cervical biopsies for pathology<br>• Post-dose cervical biopsy for transcriptome analysis<br>• Vaginal microbiome<br>• Vaginal pH, if indicated<br>• Wet mount, if indicated

12.7 Randomized Period, Visit 8: Final Safety Visit (Day 21)

The following activities take place at Visit 8 (Day 21), which is the final safety visit.

Randomized Period<br>Visit 8: Final safety visit<br>(Study Day 21)		
Component		Procedure
Administrative/ Regulatory		• Explain procedures to be performed at this visit<br>• Confirm/update locator information<br>• Provide reimbursement for study visit<br>• Schedule next study visit, if applicable
Clinical		• Update concomitant medications<br>• Assess vital signs and weight<br>• Perform complete physical exam<br>• Obtain urine sample<br>• Obtain blood samples<br>• Perform pelvic exam with relevant sampling, if indicated (after confirmation of negative pregnancy test) and bimanual exam<br>• Assess AEs
Laboratory	Urine	• β-hCG<br>• Dipstick UA<br>• Urine culture, only if indicated
	Blood	• Hematology, Chemistry, Coagulation (see Section 10.8)<br>• ADA
	Pelvic	• Vaginal pH, if indicated<br>• Wet mount, if indicated

12.8 Randomized Period, Visit 9: Closing Visit (Study Day 28)

Day 28 is a short outpatient visit, primarily to obtain a final blood specimen for ADA testing. A final urine pregnancy test will be done and AEs will be assessed. No other procedures are scheduled on Day 28, unless indicated.

<table>
<tr><th colspan="3">Randomized Period
Visit 9: Closing Visit
(Study Day 28)</th></tr>
<tr><td colspan="2">Component</td><td>Procedure</td></tr>
<tr><td colspan="2">Administrative/ Regulatory</td><td>• Explain procedures to be performed at this visit
• Confirm/update locator information
• Provide reimbursement for study visit
• Schedule next study visit, if applicable</td></tr>
<tr><td colspan="2">Clinical</td><td>• Update concomitant medications
• Obtain urine sample
• Obtain blood sample for ADA testing
• Perform physical exam, only if indicated
• Perform pelvic exam, only if indicated
• Assess AEs</td></tr>
<tr><td rowspan="3">Laboratory</td><td>Urine</td><td>• β-hCG
• Dipstick UA, only if indicated
• Urine culture, only if indicated</td></tr>
<tr><td>Blood</td><td>• ADA</td></tr>
<tr><td>Pelvic</td><td>• Vaginal pH, if indicated
• Wet mount, if indicated</td></tr>
</table>

12.9 Unscheduled Visits

Subjects will be encouraged to call or visit the clinic between regularly scheduled follow-up visits if they experience any problems. Unscheduled visits (those between regularly scheduled follow-up visits) may occur at the subject's request or as deemed necessary by the investigator or designee at any time during the study. Unscheduled visits may occur for administrative reasons, such as, if the subject has questions for study staff or requires additional study supplies. Interim contacts and unscheduled visits may also occur in response to AEs experienced by subjects, in which case study staff will assess the reported AE clinically and provide or refer the subject to appropriate medical care. All interim contacts and unscheduled visits will be documented in subject's study records and on applicable case report forms.

13. OTHER STUDY EVALUATIONS/PROCEDURES

13.1 Pharmacokinetic/Pharmacodynamic Sampling

Please refer to the Specimen Collection manual of the SSP manual for details on PK/PD sampling.

13.2 Biohazard Containment

As the transmission of HIV and other blood-borne pathogens can occur through contact with contaminated needles, blood, and blood products, appropriate blood and secretion precautions will be employed by all personnel in the drawing of blood and shipping and handling of all specimens for this study as recommended by the US Centers for Disease Control and Prevention (CDC) and the NIH (US).

All biological specimens will be transported using packaging mandated by the US Code of Federal Regulations (CFR) 42 Part 72.

All dangerous goods and materials, including diagnostic specimens and infectious substances, must be transported according to instructions detailed in the International Air Transport Association (IATA) Dangerous Goods Regulations. Biohazardous waste (including used applicators returned to the study site) will be contained according to institutional, transportation/carrier, and all other applicable regulations.

13.3 Dye Stain Assay

An analytic method utilizing a blue dye stain has been established whereby applicators inserted into the vagina can be distinguished from those that have not. The DSA was developed by the Population Council as an objective marker to assess adherence to gel use in clinical trials. The DSA is designed to help investigators determine if microbicide gels are being used and to complement safety and efficacy analyses. The DSA has been validated for use with multiple applicators containing various microbicide products (Wallace et al. 2004; Wallace et al. 2007; Katzen et al. 2011).

The polypropylene applicators being used for this study were specially designed to undergo DSA testing, and were utilized in the context of a Phase 1 trial of another microbicide candidate delivered using the same applicator (Friedland et al. 2016). Please refer to the SSP manual for further details on collection and storage of applicators, which will be tested using the DSA at the Center for Biomedical Research (CBR) of the Population Council.

13.4 Follow-up Procedures for Subjects who Discontinue Study Product

Subjects who choose to discontinue study product will be terminated from the study. A final safety assessment will be conducted for all women who are terminated early, as outlined in the SSP manual.

13.5 Subjects who Become Pregnant

In the unlikely event that a subject becomes pregnant during the study, she will discontinue study product use immediately. Procedures to be followed to ensure the subject’s safety (and that of the fetus), including pelvic sampling, will be determined based on the investigator’s judgment.

13.6 Protocol Deviations and Violations

All protocol violations and deviations will be recorded in source documentation and the Population Council will be immediately notified of all violations and deviations upon the site's awareness. Protocol deviations and violations will be reviewed by the Council Medical Monitor who will decide on a resolution.

14. SAFETY ASSESSMENTS

14.1 Safety Monitoring

AEs and SAEs will be carefully monitored and recorded throughout this study. The Investigator is responsible for continuous close safety monitoring of all subjects, and for alerting the Population Council (referred to hereafter as Sponsor) if any concerns arise. Subjects will be instructed to contact the study site staff to report any AEs they may experience. At every visit, study staff will ask each subject how they felt since their last visit, and ask if they have experienced any AEs.

The term "investigational product" for this study refers to PC-6500 or PC-535 placebo.

Subjects will also be asked if they have used any new medications, or changed any current medication regimens (both prescription and OTC medications).

14.2 Adverse Events Definitions and Reporting Requirements

14.2.1 Definition of Terms

Adverse Event: An AE is defined as any untoward medical occurrence associated with the use of a drug in humans, whether or not considered drug related. As such, an AE can be an unfavorable or unintended sign (including an abnormal laboratory finding), symptom or disease temporally associated with the use of investigational product or study participation, whether or not considered related to the product or study participation. In addition to novel events, an AE may be an exacerbation of a pre-existing medical condition that was present prior to study entry. This definition will be applied from the time a potential subject signs the ICF.

Treatment Emergent Adverse Events (TEAEs) are defined as any AE that begins on or after the first does of the study drug and up to seven days after the final study drug does, or pre-existing conditions that worsened during the same timeframe.

Non-Treatment Emergent AEs are defined as those reported between the date the informed consent form was signed and the first dose of study drug, and more than seven days after the last study drug dose.

14.2.2 Exacerbation of Pre-Existing Medical Condition

An increase in the frequency or severity/intensity of a medical condition that is present before study consent should be reported as an AE. Any medical condition that is present before study consent and that does not deteriorate should not be reported as an AE.

14.2.3 Reporting Period for Adverse Events

The reporting period for AEs is the period immediately following the signing of the ICF through seven days after the final dose in the OL period and through 14 days after the final dose in the randomized period of the study.

For the purpose of data analysis, if a non-serious AE has not resolved at the end of the study-reporting period, it will be documented as still ongoing in the CRFs. However, the Investigator must make every effort to follow the event to stabilization or resolution and report the findings/outcome to the Sponsor as soon as possible. A minimum of 3 attempts to follow up with the subject with different methods of communication (e.g., telephone, certified mail, email) should be documented.

14.2.4 Recording Adverse Events

The Investigator is responsible for assessing all AEs that occur at any time during the study. The Investigator must make a determination of relatedness, seriousness, and intensity in the CRFs for each AE.

All AEs will be recorded in the appropriate CRFs. AEs will be recorded using a recognized medical term or diagnosis that accurately reflects the event and includes the following information (when applicable):

- Date condition or event started.
- Date of resolution. If the event has not resolved at the end of the study reporting period, it will be documented as still present or ongoing in the CRFs. However, every effort should be made to obtain the outcome.
- Indication of whether the condition was pre-existing prior to study entry or not, and if yes, whether it has worsened in the severity/intensity or frequency, in which case it is reported as an AE. Conditions present prior to study entry that do not worsen in severity/intensity or frequency are not considered AEs.
- Intensity/Severity: AEs that change in intensity are recorded at the intensity level that is the most severe, as reported by the subject or observed by the Investigator.
- Relationship to investigational product, as evaluated by the Investigator.
- Possible etiologies and whether the event meets criteria as a serious adverse event (SAE) and therefore requires immediate notification to the Sponsor.
- Seriousness: If the event meets 1 or more of the regulatory criteria in the definition of an SAE (see below), the event should be classified as serious. See below for SAEs.

14.3 Serious Adverse Events Definitions and Reporting Requirements

14.3.1 Serious Adverse Event

An AE is considered “serious” if, in the view of either the Investigator or Sponsor, it:

- Results in death.
- Is life-threatening. (This term refers to an event in which the subject was at risk of death at the time of the event; it does not refer to an event that hypothetically might have caused death if it were more severe.)
- Requires inpatient hospitalization or prolongation of existing hospitalization. (In general, hospitalization signifies that the subject has been admitted [usually involving at least an overnight stay] to the hospital or emergency ward for observation and/or treatment that would not have been appropriate in the physician’s office or outpatient setting. Complications that occur during hospitalization are AEs or SAEs as defined above. If a complication prolongs hospitalization or fulfills any other serious criteria, the event is

serious. When in doubt as to whether "hospitalization" occurred or was necessary, the AE should be considered serious. Hospitalization for elective treatment or procedures of a pre-existing condition that did not worsen from baseline is not considered an AE.)

- Results in persistent or significant disability/incapacity or substantial disruption of the ability to conduct normal life functions.
- Is a congenital anomaly/birth defect.
- Is an important medical event that may not result in death, be life threatening, or require hospitalization but, based upon appropriate medical judgment, may jeopardize the subject and may require medical or surgical intervention to prevent one of the outcomes listed in the definition above. Examples of such medical events include allergic bronchospasm requiring intensive treatment in the emergency room or at home, blood dyscrasias or convulsions that do not result in patient hospitalization, or the development of drug dependency or drug abuse.

In addition, cancer and drug overdose are included in the Sponsor's classification of an SAE.

14.3.2 Study Reporting Period for Serious Adverse Events

The reporting period for SAEs is the period immediately following the signing of the ICF through seven days after the last dose in the OL period and through 14 days after the last dose in the randomized period of the study.

SAEs must be followed until resolution, even if it extends beyond the study reporting period. Resolution of an SAE is defined as the return to baseline status or stabilization of the condition with the expectation that it will remain chronic.

14.3.3 Severity/Intensity

Grading of AEs and SAEs will follow the Division of AIDS Table for Grading the Severity of Adult and Pediatric Adverse Events (DAIDS AE Grading Table), Version 2.0, November 2014. For grading female genital events, please refer to Addendum 1 (Female Genital Table for Use in Microbicide Studies), which can be found in Appendix 5.

14.3.4 Relationship to Test Article

The Investigator is responsible for assessing the relationship between the AE and the investigational product. The Investigator must determine whether there is a reasonable possibility that the investigational product caused or contributed to an AE. The relationship assessment, based on clinical judgment, often relies on the following:

- A temporal relationship between the event and administration of investigational product;
- A plausible biological mechanism for the investigational product to cause the AE;
- Another possible etiology of the AE; or
- Previous report of similar AEs associated with the investigational product or other agents in the same class.

The terms used to assess the relationship of an event to the investigational product are:

**Causality Assessment**	**Criteria for Assessment (note: re-challenge will not be done in this study)**
Highly Probably Related	The experience occurs immediately following investigational product administration, related pharmacologically (not related to underlying condition/concurrent disease or other drugs or chemicals)
Probably Related	The experience follows a reasonable temporal sequence from the time of drug administration and follows a known response pattern to the suspected investigational product and cannot be reasonably explained by other factors such as the subject's clinical state, therapeutic intervention or concomitant therapy.
Possibly Related	The experience follows a reasonable temporal sequence from the time of drug administration and/or follows a known response pattern to the investigational product but could have been produced by other factors such as the subject's clinical state, therapeutic intervention or concomitant therapy.
Unlikely Related	Improbable temporal relationship. The experience was most probably produced by other factors such as the subject's clinical state, therapeutic intervention or concomitant therapy and does not follow a known response pattern to the investigational product.
Not Related	There is not a reasonable possibility that the AE is related to the investigational product; when an AE is assessed as not related to the investigational product, an alternative etiology, diagnosis or explanation for the AE should be provided. If new information becomes available, the relationship of any AE should be reviewed again and updated, as required.
Insufficient data to assess	Selection of this rating should usually not occur in a clinical study because the Investigators have an obligation to obtain and provide this information. In exceptional instances, this rating may be used in order to avoid delay in initial reporting of fatal or life-threatening SAEs from clinical studies. Such cases should include documentation in the comments section of the CRFs/database with rationale for why an assessment could not be made.

14.4 Notification to Sponsor of Immediately Reportable Events (IRE)

All SAEs and AEs that led to premature discontinuation from the study (Immediately Reportable Events) require immediate reporting to the Sponsor whether expected or unexpected, related or unrelated. As the Sponsor of this Investigational New Drug (IND), the Population Council is responsible for complying with the reporting requirements of SAEs to the FDA in accordance with 21 CFR 312.50. The Sponsor will be responsible for the appropriate recording, review and compliance with regulatory reporting requirements to the FDA of the SAE. When any SAE or an AE that led to premature discontinuation, regardless of causality, is encountered during this study at an Investigator's site, the Investigator must immediately notify the Sponsor by e-mail: safety@popcouncil.org.

All IRE (SAE or AE that led to premature discontinuation from the study) reports must be submitted within 24 hours from the time the site staff is notified of the event to the Population Council Safety Desk (e-mail: safety@popcouncil.org or if there is a problem with e-mail, call: 202-237-9410).

Additional supporting documentation for SAEs should be provided whenever possible (with subject name redacted) to verify the medical diagnosis, including hospital discharge summaries and death certificates/autopsy reports (where applicable), surgical procedure summaries, histology reports, and imaging reports.

The Investigator should cooperate and furnish additional information, including copies of pertinent records if necessary, to assist the Sponsor's personnel in their evaluation of the event.

> **NOTE: Investigators should not wait to collect the additional information needed to fully document the event before submitting an IRE report to the Population Council.**

E-mail the following information to the Population Council Safety Desk (e-mail: safety@popcouncil.org):

1. IRE Report Form
2. Medical History CRFs
3. AE CRFs
4. Prior/Concomitant Medications CRFs
5. Relevant laboratory/diagnostic test results and medical record progress notes

Upon receipt of an IRE the Sponsor's Medical Safety Director will review the report promptly. SAEs will be reported to regulatory authorities in accordance with applicable national regulations and the Sponsor's SOP.

The Investigator will submit AE information in accordance with local regulatory agencies or other local authorities' requirements. This reporting will include site IRB-mandated reporting of AEs, SAEs and other relevant safety information.

14.5 Medical Emergencies and Emergency Protocol Deviations

In medical emergencies, the Investigator will use medical judgment and remove the subject from immediate harm. The Investigator will then immediately notify the Sponsor and the IRB regarding the type of emergency and the course of action taken.

The Investigator shall notify the Medical Safety Director, the Medical Monitor and the reviewing IRB(s) of any deviation from the investigational plan to protect the life or physical wellbeing of a subject in an emergency. Such notice shall be given as soon as possible, but in no event later than 48 hours after the emergency occurred. Except in such an emergency, prior approval by the Sponsor is required for any changes in or deviations from the protocol. All deviations must be documented.

The following contact will be used for all communications with the study Medical Safety Director:

Mohcine Alami, MD
Director, Global Medical Safety
Population Council
4301 Connecticut Avenue NW Suite 280
Washington, DC 20008
(202) 237-9410
malami@popcouncil.org

14.6 Unblinding Procedures (randomized period only)

Although it is advantageous to retain the blind for all subjects prior to final study analysis, when a serious adverse reaction (serious, unexpected and related to the investigational product) is judged reportable on an expedited basis, the blinding should be broken for the subject. Only interventions determined to be active will be reported to the FDA by the Sponsor. Unblinded interventions of placebo do not need to be reported to the FDA.

A statistician not involved in the trial will generate and be responsible for keeping the randomization schedule. Investigators must submit all unblinding requests for SAEs to the Population Council for evaluation and final decision. The decision to unblind will be made by the Sponsor, in consultation with the site Investigator.

The Medical Safety Director, a physician or their designee will determine immediately within 24 hours or at the earliest possible time if the blind should be broken.

The Medical Safety Director will provide written authorization to Regulatory Affairs, the "Holder" of the randomization schedule or their designee to break the blind and notify in writing the study PI immediately within 24 hours.

If the blind is broken (e.g., for reasons of subject safety only) and determined to be an active intervention, these cases should be reported to the FDA in an IND safety report according to 21 CFR 312.32(c).

14.7 Sponsor Reporting Obligations

The Sponsor will forward all reportable AEs to the appropriate regulatory authorities and the Investigator according to 21 CFR 312, and any other applicable regulations.

15. CLINICAL MANAGEMENT

Guidelines for clinical management and temporary product hold/permanent discontinuation of study product are outlined in this section. In general, the Investigator has the discretion to hold study product temporarily at any time if s/he feels that continued product use would be harmful to the subject or interfere with treatment deemed clinically necessary. Unless otherwise specified below, the Investigator should immediately consult the Council's Medical Monitor for further guidance on resuming study product, continuing the hold temporarily, or progressing to permanent discontinuation of study product. The Investigator will document all temporary product holds and permanent discontinuations on applicable CRFs. AEs that led to premature discontinuation will be reported immediately to the Sponsor using the IRE form.

15.1 Grading System

Grading of AEs should follow the DAIDS AE Grading Table, Version 2.0, November 2014. For grading female genital events, please refer to Addendum 1 (Female Genital Table for Use in Microbicide Studies). Tables can be found in Appendix 5. The final determination of the grade will be by the consensus of the PI, the Medical Monitor, and the Medical Safety Director for this study.

15.2 Dose Modification Instructions

No dose modifications will be undertaken during this study.

15.3 Discontinuation of Study Product in Response to Adverse Events

15.3.1 Grade 1 AEs and Unrelated Grade 2 AEs

In general, individual subjects who develop a Grade 1 AE regardless of relatedness, or an unrelated Grade 2 AE may continue use of their assigned study product per protocol, at the Investigator's discretion.

15.3.2 Related Grade 2 AEs; Grade 3 and 4 AEs

Individual subjects who develop a related Grade 2 AE, or Grade 3 AE, regardless of relatedness to study drug, will be evaluated by the PI, Medical Monitor and Medical Safety Director for possible discontinuation from the study. Individual subjects who develop a Grade 4 AE, regardless of relatedness to study drug, will be evaluated by the PI, Medical Monitor and Medical Safety Director and discontinued from the study.

15.3.3 Halting enrollment

In the circumstance where any of the following criteria are met and confirmed by the PI, the Medical Monitor, and the Medical Safety Director for this study, enrollment in this study will be stopped:

- Two Grade 3 AEs that are similar and attributed to the active study drug;
- Any Grade 4 AE attributed to the active study drug.

15.4 Management of Specific Toxicities

There are no known specific toxicities of GRFT, but because it is a mannose binding lectin, mannose could be an antidote for unexpected toxicity.

15.5 Criteria for Early Termination of Study Participation

Subjects may voluntarily withdraw from the study for any reason at any time. In addition, the Investigator may withdraw subjects from the study for any of the following reasons:

- Indeterminate or positive HIV-1 rapid test
- Acquisition of an STI
- Other medical reasons, including symptomatic vaginal infection diagnosed after enrollment
- Pregnancy
- Report of use of post-exposure prophylaxis (PEP) for HIV exposure
- Report of use of prohibited medications as described in Section 9.8.2
- Subject is unable or unwilling to comply with required study procedures or has failed to follow protocol requirements, including failure to use study product, in a manner judged by the site investigator to be severe enough to affect study outcomes

- Subject might be put at undue risk to their safety and wellbeing by continuing product use, according to the judgment of the Investigator.
- The study Sponsor, government or regulatory authorities, including FDA and Office for Human Research Protections (OHRP), or site IRB terminates the study prior to its planned end date.

Every reasonable effort should be made to complete a final evaluation of subjects who withdraw or are withdrawn from the study prior to completing follow-up. Study staff members will record the reason(s) for all withdrawals in subject's study records.

15.6 Subject Withdrawal

Subjects withdrawing from the study will not be replaced. Safety data will be included in the analysis from any subjects who withdraw. All efforts should be made to contact any subject who decides to withdraw from the trial. All final safety evaluations should be performed. The main reason for discontinuation prior to completing follow up will be recorded. If a subject discontinues due to an AE, the reasons must be submitted on the IRE forms for SAEs and AE leading to discontinuation. The Council's Study Manager will be informed with respect to enrollment and withdrawals from the study.

15.7 Pregnancy and Pregnancy Outcome

Pregnant women are excluded from this study. If a subject becomes pregnant at any time during the course of the study, she will discontinue study product use immediately and will be terminated from the study. The subject will be followed beyond the last study visit until the outcome of her pregnancy. Pregnancy-related data will be collected using a pregnancy CRF for any pregnancy detected during the study. Follow-up for pregnancy outcome, birth defect or major anomaly outcome, and any associated AE/SAE will be reported on the CRFs and to the Council.

Pregnancy testing will be performed at weekly study visits and subjects will be encouraged to report all signs or symptoms of pregnancy to study staff. The Investigator will counsel any subject who becomes pregnant regarding possible risks to the fetus according to site SOPs.

16. QUALITY CONTROL AND ASSURANCE

The study will be conducted in accordance with ICH Good Clinical Practice (GCP) and the SOPs of the Population Council and the study site.

The Council will conduct a site initiation visit prior to initiation of data collection.

During the conduct of the trial, the Council or its designees will conduct periodic monitoring visits to ensure that the protocol and GCPs are being followed. The monitors may review source documents to confirm that the data recorded on CRFs is accurate. The Investigator and institution will allow the Council's monitors or its designees and appropriate regulatory authorities direct access to source documents to perform this verification.

The trial site may be party to review by the Population Council or local IRB and/or to quality assurance audits performed by the Council and/or its designees, and to inspection by appropriate regulatory authorities. It is important that the Investigator(s) and their relevant personnel are

available during monitoring visits and possible audits or inspections, and that sufficient time is devoted to the process.

The study products (PC-6500 and PC-535) will be manufactured at the Population Council's Center for Biomedical Research (CBR) in a lab certified in accordance with current Good Manufacturing Practices (GMP).

17. STATISTICAL METHODS

17.1 Overview and Summary of Design

This is a Phase 1 single-site, double-blind, parallel-group, randomized, placebo-controlled trial, preceded by an OL active treatment safety run-in period. Subjects in the OL period (n=7) will receive only one dose of gel, administered in the clinic. The main, randomized period of the study will enroll up to 20 subjects dosing once per day for 14 consecutive days with a mix of in-clinic and at-home dosing. Product randomization will be approximately 7:3 (PC-6500:PC-535) and subjects will also be randomized (approximately 1:1) to one of two time points for CVL specimen collection after the first dose. Details will be described in a statistical analysis plan (SAP).

17.2 General Considerations

In general, descriptive statistics will be presented for demographic and baseline characteristics and for each of the endpoints for each treatment group and for the two treatment groups combined. Point estimates and corresponding 2-sided 95% confidence intervals will be presented for endpoints, where appropriate.

17.3 Determination of Sample Size

No formal sample size calculations were carried out for this study. A sample size of 20 subjects (14 active), allowing for a 10% drop out rate, is considered sufficient to evaluate the safety of PC-6500 and to assess the PK of GRFT.

17.4 Analysis Populations

The "all subjects" population includes all subjects who have been enrolled into the study.
The "safety population" includes all subjects who have inserted at least one dose of the study gel.
The "randomized population" includes all subjects who were randomized to treatment.

17.5 Endpoints

17.5.1 Primary

Safety

- Number and percent of subjects with TEAEs, SAEs, and TEAEs leading to premature discontinuation.
- Number, percent and, in the investigator's judgment, medical significance of abnormalities in physical exams, pelvic exams, and biopsies once product has been administered.
- Number, percent and, in the investigator's judgment, medical significance of abnormalities in clinical laboratory parameters, once product has been administered.

Pharmacokinetics (PK)
Evaluation of concentrations of GRFT in blood during and after a single dose of PC-6500 (the initial dose); then during and after 14 days of daily use of PC-6500 vaginal gel, with an estimation of:

- Area under the time-concentration curve ($AUC_{0\text{-last}}$; $AUC0_{-\infty}$)
- Peak concentration (C_{max})
- Trough concentration (C_{min})
- Time to blood concentration (T_{max})
- Elimination half-life ($T_{½}$)
- Apparent clearance (Cl/F)

Details will be elaborated in the SAP.

17.5.2 Exploratory

1) Post-treatment concentrations of GRFT and CG in CVLs.
2) Changes between baseline and after treatment in anti-HIV activity of CVLs in cell-based assays and mucosal explants.
3) Changes between baseline and after treatment in transcriptome analysis of gene expression in tissues and in concentrations of immune mediators in CVL.
4) Changes between baseline and after treatment in the vaginal microbiome.
5) Changes between baseline and after treatment in ADA detection in blood.
6) Association between CVL drug levels with anti-HIV activity.
7) Changes between baseline and after treatment in anti-*E. coli* activity in CVLs.

17.6 Safety Analysis

Safety summaries will be performed on the safety population.

AEs will be coded in accordance with the current version of the Medical Dictionary for Regulatory Activities (MedDRA). Summary of all AEs will be based on TEAEs which include all events that occur on or after the first dosing date and on or before the 7th day after the last dosing date in the OL period of the study and on or before the 14th day after the last dosing date in the randomized period.

The number and percent of the subjects for each AE and SAE will be summarized by system organ class and preferred term for each treatment group.

The summaries of physical exam (including pelvic exam) results will be presented by treatment group for each visit and for the changes from baseline to each visit. Shift tables will be presented to summarize the changes of abnormalities for each parameter.

Changes in vital signs will be summarized for each treatment group.

Clinical laboratory evaluations will be summarized by treatment group for each visit. The abnormalities for each parameter will be summarized for each treatment group. Changes in baseline values and in absolute values for each laboratory parameter will be summarized for each treatment group using shift tables and summary statistics.

17.7 Statistical Analysis of PK Measurements

If absorption is observed, then PK analysis will be performed. The aim of the PK analysis for this study is to gather exploratory information on absorption, distribution and elimination (not to compare bioavailability using multiple formulations or doses). Drug concentrations and drug exposure will be determined using blood samples for GRFT concentrations.

Blood concentrations of GRFT will be assessed at multiple time points during the trial. In the OL period, sampling will occur before dosing and at 0.5, 1, 2, 3, 4, 6, 8, 10, 12 and 24h after the single dose. In the randomized portion, sampling will occur before dosing on Days 1, 3, 5, 8, 11 and 14; on Day 1 at 4h or 8h post-dose, according to the randomization scheme; on Day 14 at 0.5, 1, 2, 3, 4, 6, 8h post-dose; and on Day 15, 24h post-dose 14. If absorption occurs, C_{max}, C_{min}, T_{max}, $T_{1/2}$, apparent clearance (Cl/F), $AUC_{0\text{-}last}$; and $AUC_{0\text{-}\infty}$ after dosing through the end of the elimination period will be computed as the data allow. For all PK concentrations and computed PK parameters, the number of observations, means, standard deviations, medians, first and third quartiles, and ranges will be presented.

17.8 Statistical Analysis of Exploratory Endpoints

Concentrations of GRFT and CG in CVLs will be measured for each subject before and after the first dose. All subjects in the OL period will have CVLs collected 24h post-dose. In the main study, one-half of subjects will be randomized to CVL specimen collection at 4h after the first dose (n=10) and the other half will be randomized to CVL specimen collection 8h after the first dose (n=10). Mean concentration, minimum and maximum will be calculated and concentrations will be summarized for subjects sampled at the three different time points.

Log-normal generalized linear mixed models will be used to analyze changes between baseline and post-gel use in anti-HIV activity of CVL in explants and in concentrations of immune mediators in CVL.

Anti-HIV activity in CVL will be also assessed using a TZM-bl assay with the $HIV\text{-}1_{ADA\text{-}M}$ laboratory strain. Assays will be performed on serial dilutions to establish a dose-response curve. GraphPad Prism software will be used to prepare curve-fitting analysis and to calculate the median cytotoxicity concentration (CC_{50}) and the half-maximal effective concentration values (EC_{50}, based on CVL dilution or drug concentrations) for all CVLs. Correlations between levels of GRFT in CVL and its anti-HIV activity will be calculated.

Transcriptome will be analyzed using Deseq2 and Voom software to evaluate statistical significance of changes in gene expression in tissue between baseline and post gel application.

Vaginal swabs will be collected to determine potential changes in microbiome post-gel exposure relative to pre-exposure. The unique fraction metric (UniFrac) will be used to measure the differences between microbial communities using the phylogenetic information. Linear discriminant analysis Effect Size (LEfSe) will be employed to compare the microbiome communities before and after gel application.

ADA will be assessed qualitatively both in baseline serum samples and in serum samples collected after daily GRFT use (Day 14), one week after the last gel application (Day 21) and two weeks after the last gel application (Day 28). Shift tables will be presented by treatment arm to summarize changes in detection of ADA between baseline and Days 14, 21 and 28.

Mean E. coli inhibitory activity of CVL samples collected at baseline and at different time points after gel application will be calculated. Comparisons of the inhibitory activity of CVLs collected at different time points after gel application and versus CVL collected at baseline will be performed by paired t-tests or Wilcoxon-signed rank tests.

For all the computed PD parameters, number of observations, means, standard deviations, medians, and ranges will be presented by treatment arm and specimen collection time point.

RSID testing of CVLs or vaginal swabs will be done to determine if unprotected (condomless) sex occurred because semen could have an impact on some exploratory analyses. Specimens that test positive may be eliminated from some of the final analyses, to be further described in the SAP.

17.9 Exposure Analysis

Exposure to study drug will be measured as the total number of applicators used by subjects. The number of applicators inserted will be calculated as the total number of applicators inserted in the clinic (OL and randomized period) plus the number of applicators inserted at home (randomized period), per DSA testing. Exposure will be summarized by treatment group with means, standard deviations, medians, 1st and 3rd quartiles and ranges.

18. DATA HANDLING

18.1 Data Management Responsibilities

Study CRFs will be developed by the Population Council in conjunction with the study site. DataFax's electronic data capture (EDC) system will be used to record study data collected at the site on electronic case report forms (eCRFs).

The site will be responsible for entering the data electronically through the iDataFax application. Screening data should be entered as soon as possible and prior to enrollment. After enrollment, data should be entered within 7 days of each subject's visit. Periodically, the monitor will review study documents to verify compliance with the protocol. The accuracy of the data compared to source documentation will also be reviewed.

As a result of the data review process, corrections or changes to the data may be required. Discrepancies or questions concerning the data may be sent to the site in addition to queries that may be triggered during data entry of the eCRFs. The site will respond promptly to requests for clarifications and corrections.

18.2 Source Documents

The study site will maintain source data/documents in accordance with Population Council policy. The Investigator will maintain, and store securely, complete, accurate and current study records throughout the study. In accordance with US regulations regarding testing investigational products, the Investigator will maintain all study documentation for at least two years following the date of marketing approval for the study products being tested for the indication for which they were studied. If no marketing application is filed, or if the application is not approved, the records will be retained for two years after the investigation is discontinued and the US FDA is notified.

Study records must be maintained on site for the entire period of study implementation. Thereafter, instructions for record storage will be provided by the Council. No study records may be moved to an off-site location or destroyed prior to receiving approval from the Council.

19. CLINICAL SITE MONITORING

Study monitoring will be carried out by a Clinical Research Associate (CRA) contracted by the Population Council, or a Population Council staff monitor, in accordance with the current Council monitoring SOP. Monitoring visits will consist of a site initiation visit, periodic monitoring visits and a site close-out visit. Site visits will include the following:

- Review informed consent forms, subject eligibility, procedures, and documentation.
- Assess compliance with the study protocol, GCP guidelines, and applicable US regulatory requirements, including CFR Title 45 Part 46 and Title 21 Parts 50, 56, and 312.
- Perform 100% source document verification to ensure the accuracy and completeness of study data.
- Verify proper collection and storage of biological specimens.
- Verify proper storage, dispensing, and accountability of investigational study products.
- Assess implementation and documentation of internal site quality management procedures.
- The Investigator will allow study monitors to inspect study facilities and documentation (e.g., ICFs, clinic and laboratory records, other source documents, CRFs), as well as observe the performance of study procedures.
- The Investigator also will allow inspection of all study-related documentation by authorized representatives of the Population Council, FDA, OHRP and local and US regulatory authorities, IRBs and study staff.

A site visit log will be maintained at the study site to document all visits.

20. HUMAN SUBJECTS PROTECTION

The Investigator must ensure that each study subject, or her legally acceptable representative, is fully informed about the nature and objectives of the trial and possible risks associated with participation. The Investigator, or a person designated by the Investigator, will obtain written informed consent from each subject or the subject’s legally acceptable representative before any trial-specific activity is performed. The ICF used in this trial, and any changes made during the course of the trial, must be prospectively approved by the relevant IRBs before use. The Investigator will retain the original of each subject’s signed consent.

The site Investigator will make efforts to minimize risks to subjects. Before beginning the study, the Investigator will have obtained IRB approval and the Council will have submitted the protocol to the FDA. The Investigator will permit audits by the Council, FDA, OHRP and local and US regulatory authorities, IRBs and study staff or any of their appointed agents.

20.1 Institutional Review Boards

The participating institution is responsible for assuring that this protocol, the associated site-specific ICFs, and study-related documents (such as subject education and recruitment materials) are reviewed and approved by an IRB responsible for oversight of research conducted

at the study site. Any amendments to the protocol must be approved by the responsible IRBs prior to implementation.

Subsequent to the initial review and approval, the responsible IRBs must review the study at least annually. The Investigator will submit safety and progress reports to the IRBs at least annually and within three months after study termination or completion. These reports will include the total number of subjects enrolled in the study, the number of subjects who completed the study, all changes in the research activity, and all unanticipated problems involving risks to human subjects or others.

20.2 Study Coordination

The Population Council, Inc. (Sponsor) holds the IND application for this study. Assignment of all Sponsor responsibilities for this study will be specified in a Clinical Trials Agreement (CTA) executed by the Population Council and Einstein. Study implementation will be directed by this protocol, which may not be amended without prior written approval from the Sponsor. Standardized study-specific training will be provided to the site by the Sponsor.

Close coordination between protocol team members (Sponsor and Study Site) is necessary to track study progress, respond to queries about proper study implementation, and address other issues in a timely manner. The Sponsor will address issues related to study eligibility and AE management and reporting as needed to assure consistent case management, documentation, and information sharing across sites. Rates of accrual, adherence, follow-up, and AE incidence will be monitored closely by the Council Study Manager.

20.3 Risk-Benefit Statement

20.3.1 Risks

It is not expected that this trial will expose subjects to unreasonable risk. However, as this is the first time GRFT is being tested in humans, there may be unforeseen risks.

The main risk for subjects in the main study is the pain, discomfort, and possibility of bleeding from the vaginal biopsies (and less so, the cervical biopsies).

In addition, phlebotomy may lead to excessive bleeding, discomfort, feelings of dizziness or faintness, and/or bruising, swelling and/or infection. Pelvic examination may cause mild discomfort and/or embarrassment. Taking of pelvic specimens may cause spotting during or after the pelvic exam. Disclosure of HIV and STI status may cause worry, sadness or depression.

Participation in clinical research includes the risks of loss of confidentiality and discomfort with the personal nature of questions when discussing sexual behaviors. Although the study site will make every effort to protect subject privacy and confidentiality, it is possible that a subject's involvement in the study could become known to others, and that social harms may result. For example, in an effort to comply with local requirements to report communicable diseases including HIV-1, sites may be required to report these diseases to local health authorities.

20.3.2 Benefits

Subjects in this study will experience no direct benefit. Subjects and others may benefit in the future from information learned from this study. Specifically, information learned in this study may lead to the development of safe and effective interventions to prevent HIV transmission. Subjects also may appreciate the opportunity to contribute to the field of HIV-prevention research.

Subjects will receive HIV and STI testing, physical examinations, and pelvic examinations. If a subject tests positive for HIV or other STIs, she will meet with the site investigator to discuss the results.

Women found to be HIV infected will be referred to available sources of medical and psychosocial support, and to local research studies for HIV-infected adults. Women who test positive for other RTI/STI or develop other medical conditions identified as part of the study screening and/or follow-up procedures will be offered treatment and referred to MMC for management of the condition.

20.3.3 Compensation

Subjects will be compensated for each clinic visit, ranging from approximately $50 for a short outpatient visit to $250 for a 12-hour PK visit. At each visit, subjects will be provided with or reimbursed for refreshments and/or meals, as appropriate.

20.4 Informed Consent Process

Written informed consent will be obtained from each study subject prior to screening. Separate written informed consent will be obtained for long-term specimen storage and possible future testing, although consent for long-term specimen storage is not required for study participation. In obtaining and documenting informed consent, the Investigator will comply with applicable local and US regulatory requirements and will adhere to GCP and to the ethical principles that have their origin in the Declaration of Helsinki. Clinic staff must document the informed consent process, including assessment of the subject's competence to consent.

Subjects will be provided with a copy of the ICF. The informed consent process will cover all elements of informed consent required by research regulations. In addition, the informed consent process will address the following topics of specific importance to this study:

- The unknown safety and unproven efficacy of GRFT
- The possibility of being randomized to the placebo (randomized period only)
- The need to abstain from sexual intercourse during the study (beginning 48h before enrollment through Day 8 [OL] or 96h before enrollment through Day 17 [randomized])
- The importance of adherence to the study visit and procedures schedule
- The potential medical risks of study participation, particularly, painful vaginal biopsies (randomized) and what to do if such risks are experienced
- The lack of benefits of study participation
- The distinction between research and clinical care
- The right to withdraw from the study at any time

20.5 Confidentiality

All study procedures will be conducted in private and every effort will be made to protect subject privacy and confidentiality to the extent possible.

All study-related information will be stored securely at the study site. All subject information will be stored in locked areas with access limited to study staff. All laboratory specimens, study data,

and administrative forms will be identified by PTIDs only to maintain confidentiality. All records that contain names or other personal identifiers, such as locator forms and informed consent forms, will be stored separately from study records identified by code number. All local databases will be secured with password protected access systems. Forms, lists, logbooks, appointment books, and any other listings that link subjects' ID numbers to identifying information will be stored in a separate, locked file in an area with limited access. Subjects' study information will not be released without their written permission, except as necessary for review, monitoring, and/or auditing by the following:

- Representatives of the US Federal Government, including the US FDA, the US OHRP
- Representatives of Population Council and its monitors
- Representatives of Einstein and Montefiore
- Study staff

21. PUBLICATION POLICY

The Council policies and a Clinical Trial Agreement between the Council and Einstein will govern publication of the results of this study.

REFERENCES

Abdool Karim Q, Abdool Karim SS, Frohlich JA, et al. (2010). Effectiveness and safety of tenofovir gel, an antiretroviral microbicide, for the prevention of HIV infection in women. *Science* 329(5996): 1168-1174.

Alexandre KB, Gray ES, Pantophlet R, et al. (2011). Binding of the mannose-specific lectin, griffithsin, to HIV-1 gp120 exposes the CD4-binding site. *J Virol* 85(17): 9039-9050.

Auvert B, Taljaard D, Lagarde E, et al. (2005). Randomized, controlled intervention trial of male circumcision for reduction of HIV infection risk: the ANRS 1265 Trial. *Plos Medicine* 2(11): e298.

Baeten JM, Donnell D, Ndase P, et al. (2012). Antiretroviral prophylaxis for HIV prevention in heterosexual men and women. *N Engl J Med* 367(5): 399-410.

Baeten JM, Palanee-Phillips T, Brown ER, et al. (2016). Use of a Vaginal Ring Containing Dapivirine for HIV-1 Prevention in Women. *N Engl J Med* 375(22): 2121-2132.

Bailey R, Moses S, Parker C, et al. (2007). Male circumcision for HIV prevention in young men in Kisumu, Kenya: a randomised controlled trial. *Lancet* 369(9562): 643-656.

Banerjee K, Michael E, Eggink D, et al. (2012). Occluding the mannose moieties on human immunodeficiency virus type 1 gp120 with griffithsin improves the antibody responses to both proteins in mice. *AIDS Res Hum Retroviruses* 28(2): 206-214.

Barnable P, Calenda G, Ouattara L, et al. (2014). A MIV-150/zinc acetate gel inhibits SHIV-RT infection in macaque vaginal explants. *PLoS One* 9(9): e108109.

Barton CL (2014). Evaluation of the safety and pharmacokinetic profile of the broad spectrum antiviral lectin griffithsin. Department of Pharmacology and Toxicology, University of Louisville School of Medicine: 186.

Barton C, Kouokam JC, Lasnik AB, et al. (2014). Activity of and effect of subcutaneous treatment with the broad-spectrum antiviral lectin griffithsin in two laboratory rodent models. *Antimicrob Agents Chemother* 58: 120–127.

Bollen L, Kilmarx P and Wiwatwongwana P (2002). *Photo Atlas for Microbicide Evaluation*. Bangkok, Thailand MOPH-U.S. CDC Collaboration.

Carraguard Phase II South Africa Study Team. (2010). Expanded safety and acceptability of the candidate vaginal microbicide Carraguard(R) in South Africa. *Contraception* 82(6): 563-571.

Chatterjee A, Ratner DM, Ryan CM, et al. (2015). Anti-Retroviral Lectins Have Modest Effects on Adherence of Trichomonas vaginalis to Epithelial Cells *In Vitro* and on Recovery of Tritrichomonas foetus in a Mouse Vaginal Model. *PLoS One* 10(8): e0135340.

Cohen MS, Chen YQ, McCauley M, et al. for the HPTN 052 Study Team (2011). Prevention of HIV-1 infection with early antiretroviral therapy. *N Engl J Med* 365(6): 493-505.doi:10.1056/NEJMoa1105243.

Elias C and Heise L (1994). Challenges for the development of female-controlled vaginal microbicides. *AIDS* 8(1): 1-9.

Emau P, Tian B, O'Keefe B R, et al. (2007). Griffithsin, a potent HIV entry inhibitor, is an excellent candidate for anti-HIV microbicide. *J Med Primatol* 36(4-5): 244-253.

FACTS. (2015). FACTS 001 Study. Retrieved November 19, 2015. Available at https://factsconsortium.wordpress.com/facts-001-study.

Fernández-Romero JA, Abraham CJ, Rodriguez A, et al. (2012). Zinc acetate/carrageenan gels exhibit potent activity *in vivo* against high-dose herpes simplex virus 2 vaginal and rectal challenge. *Antimicrob Agents Chemother* 56(1): 358-368.

Garg S, Goldman D, Krumme M, et al. (2010). Advances in development, scale-up and manufacturing of microbicide gels, films, and tablets. *Antiviral Res* 88(Suppl 1): S19-29.

Grant RM, Lama JR, Anderson PL, et al. (2010). Preexposure chemoprophylaxis for HIV prevention in men who have sex with men. *N Engl J Med* 363(27): 2587-2599.

Gray R, Kigozi G, Serwadda D, et al. (2007). Male circumcision for HIV prevention in men in Rakai, Uganda: a randomised trial. *Lancet* 369(9562): 657-666.

Huskens D and Schols D (2012). Algal lectins as potential HIV microbicide candidates. *Mar Drugs* 10(7): 1476-1497.

Ishag H, Li C, Huang L, et al. (2013). Griffithsin inhibits Japanese encephalitis virus infection *in vitro* and *in vivo*. *Arch Virol* 158: 349-358.

Katzen LL, Fernández-Romero JA, Sarna A, et al. (2011). Validation of a dye stain assay for vaginally inserted hydroxyethylcellulose-filled microbicide applicators. *Sex Transm Dis* 38(11): 1050-1055.

Kilmarx PH, Blanchard K, Chaikummao S, et al. (2008). A randomized, placebo-controlled trial to assess the safety and acceptability of use of carraguard vaginal gel by heterosexual couples in Thailand. *Sex Transm Dis* 35(3): 226-232.

Kilmarx PH, van de Wijgert JH, Chaikummao S, et al. (2006). Safety and acceptability of the candidate microbicide Carraguard in Thai Women: findings from a Phase II Clinical Trial. *J Acquir Immune Defic Syndr* 43(3): 327-334.

Kizima L, Rodriguez A, Kenney J, et al. (2014). A potent combination microbicide that targets SHIV-RT, HSV-2 and HPV. *PLoS One* 9(4): e94547.

Kouokam JC, Huskens D, Schols D, et al. (2011). Investigation of griffithsin's interactions with human cells confirms its outstanding safety and efficacy profile as a microbicide candidate. *PLoS One* 6(8): e22635.

Levendosky K, Mizenina O, Martinelli E, et al. (2015). Griffithsin and carrageenan combination to target HSV-2 and HPV. *Antimicrob Agents Chemother* 59(12): 7290-7298.

McCormack S, Dunn DT, Desai M, et al. (2016). Pre-exposure prophylaxis to prevent the acquisition of HIV-1 infection (PROUD): effectiveness results from the pilot phase of a pragmatic open-label randomised trial. *Lancet* 387(10013): 53-60.

McLean CA, van de Wijgert JH, Jones HE, et al. (2010). HIV genital shedding and safety of Carraguard use by HIV-infected women: a crossover trial in Thailand. *AIDS* 24(5): 717-722.

Meuleman P, Albecka A, Belouzard S, et al. (2011). Griffithsin has antiviral activity against hepatitis C virus. *Antimicrob Agents Chemother* 55(11): 5159-5167.

Molina J-M, Capitant C, Spire B, et al. (2015). On-demand preexposure prophylaxis in men at high risk for HIV-1 infection. N Engl J Med 373(23): 2237-2246.

Moncla BJ, Pryke K, Rohan LC, et al. (2011). Degradation of naturally occurring and engineered antimicrobial peptides by proteases. *Adv Biosci Biotechnol* 2(6): 404-408.

Moncla BJ, Pryke K, Rohan LC, et al. (2012). Testing of viscous anti-HIV microbicides using Lactobacillus. *J Microbiol Methods* 88(2): 292-296.

Mori T, O'Keefe BR, Sowder RC, 2nd, et al. (2005). Isolation and characterization of griffithsin, a novel HIV-inactivating protein, from the red alga Griffithsia sp. *J Biol Chem* 280(10): 9345-9353.

Moulaei T, Shenoy SR, Giomarelli B, et al. (2010). Monomerization of viral entry inhibitor griffithsin elucidates the relationship between multivalent binding to carbohydrates and anti-HIV activity. *Structure* 18(9): 1104-1115.

Nel A, van Niekerk N, Kapiga S, et al. (2016). Safety and efficacy of a dapivirine vaginal ring for HIV prevention in women. *N Engl J Med* 375(22): 2133-2143.

Nixon B, Stefanidou M, Mesquita PM, et al. (2013). Griffithsin protects mice from genital herpes by preventing cell-to-cell spread. *J Virol* 87(11): 6257-6269.

O'Keefe BR, Vojdani F, Buffa V, et al. (2009). Scaleable manufacture of HIV-1 entry inhibitor griffithsin and validation of its safety and efficacy as a topical microbicide component. *Proc Natl Acad Sci U S A* 106(15): 6099-6104.

Rodriguez A, Kleinbeck K, Mizenina O, et al. (2014). *In vitro* and *in vivo* evaluation of two carrageenan-based formulations to prevent HPV acquisition. *Antiviral Res* 108: 88-93.

Skoler-Karpoff S, Ramjee G, Ahmed K, et al. (2008). Efficacy of Carraguard for prevention of HIV infection in women in South Africa: a randomised, double-blind, placebo-controlled trial. *Lancet* 372(9654): 1977-1987.

Takebe Y, Saucedo CJ, Lund G, et al. (2013). Antiviral lectins from red and blue-green algae show potent *in vitro* and *in vivo* activity against hepatitis C virus. *PLoS One* 8(5): e64449.

Thigpen MC, Kebaabetswe PM, Paxton LA, et al. (2012). Antiretroviral preexposure prophylaxis for heterosexual HIV transmission in Botswana. *N Engl J Med* 367(5): 423-434.

Tolley EE, Friedland B, Gafos M, et al. (2014). Socioeconomic and behavioral factors influencing choice, adherence and success of microbicide formulations. J Neves and B Sarmento. Singapore, Pan Stanford Publishing.

UNAIDS (2014). The Gap Report. Geneva, Switzerland, Joint United Nations Programme on HIV/AIDS.

UNAIDS (2015). Empower young women and adolescent girls: Fast-Track the end of the AIDS epidemic in Africa.

van de Wijgert JH, Braunstein SL, Morar NS, et al. (2007). Carraguard Vaginal Gel Safety in HIV-Positive Women and Men in South Africa. *J Acquir Immune Defic Syndr* 46(5): 538-546.

van der Straten A, Montgomery ET, Musara P, et al., for the Microbicide Trials Network-003D Study Team (2015). Disclosure of pharmacokinetic drug results to understand nonadherence. *AIDS* 29(16): 2161-2171.

Wallace A, Thorn M, Maguire RA, et al. (2004). Assay for establishing whether microbicide applicators have been exposed to the vagina. *Sex Transm Dis* 31(8): 465-468.

Wallace AR, Teitelbaum A, Wan L, et al. (2007). Determining the feasibility of utilizing the microbicide applicator compliance assay for use in clinical trials. *Contraception* 76(1): 53-56.

Wawer MJ, Makumbi F, Kigozi G, et al. (2009). Circumcision in HIV-infected men and its effect on HIV transmission to female partners in Rakai, Uganda: a randomised controlled trial. *Lancet* 374(9685): 229-237.

Ziolkowska NE, O'Keefe BR, Mori T, et al. (2006). Domain-swapped structure of the potent antiviral protein griffithsin and its mode of carbohydrate binding. *Structure* 14(7): 1127-1135.

Ziolkowska NE, Shenoy SR, O'Keefe BR, et al. (2007). Crystallographic, thermodynamic, and molecular modeling studies of the mode of binding of oligosaccharides to the potent antiviral protein griffithsin. *Proteins* 67(3): 661-670.

Appendix 1: Schedule of Visits and Procedures for OL Safety Run-In (n=7)

**Visit/Procedure**		**Visit 0**	**Visit 1**			**Visit 2**	**Visit 3**	**Unscheduled Visits**
Study Day		*≤30d before Day1*	*Enrollment/Day 1*			*Day 2*	*Closing/ Day 8*	
		Screening	*Pre-dose*	**Single Dose in clinic**	*Post-dose*	*24h post-dose*	*n/a*	
Informed consent		X						
Assign participant ID number		X						
Record/Confirm locator information		X	X			X	X	X
Demographics		X						
Assess eligibility		X	X					
Medical history		X						
Record medication history/ concomitant meds		X	*		*	*	*	*
HIV-1 with confirmatory test		X						
HSV-1 and HSV-2 test		X						
EKG			X			X		
Urine pregnancy test		X	X				X	*
Vital signs§		X	X		X	X	X	*
Physical exam***		X	*		*	*	X	*
Clinical labs (hematology, chemistry, coagulation)		X	X		X	X	X	*
Urinalysis		X	*		*	*	X	*
Urine culture		*	*		*	*	*	*
Pelvic exam (with breast exam at screening)		X	X		*	*	X	*
Vaginal swab for wet mount		*	*		*	*	*	*
Vaginal strip for pH		*	*		*	*	*	*
NAAT for NG/CT/TV		X						
Treat or prescribe treatment for RTI/STI		*	*		*	*	*	*
Pap smear**		*						
Counseling: HCT, risk reduction; adherence		X	*		X	X	X	*
Collect applicator for DSA testing (positive control)					X			
**1º Endpoint: Safety**	AEs		X		X	X	X	X
**1º Endpoint: PK**	Blood		X		X (0.5,1,2,3,4, 6,8,10,12h post-dose)	X (24h post-dose)		
**Exploratory:**	CVL (API concentration, PD, E. coli & RSID)		X			X		
	Swab for vaginal microbiome		X			X		

*If indicated. **If no appropriately documented history of Pap test and completed follow-up of any abnormal Pap tests consistent with ACOG bulletins #140 and #168.***Complete exam at screening and Visit 3 (Day 8); targeted exam at other visits only if indicated. §Weight only at screening; height at screening and at the final safety visit (Day 8).

Appendix 2: Schedule of Visits and Procedures for Randomized Period (n=20)

**Visit Number**	**Visit 0**	**Visit 1**	**Visit 2**			**Visit 3**			**Visit 4**			**Visit 5**			**Visit 6**			**Visit 7**	**Visit 8**	**Visit 9**	**Unsche-duled Visit**
Cycle Day		*21-28 (not on HC) 15-22 (if on HC)*	*7-10 (not on HC) 1-4 (if on HC)*																		
Study Day	*≤45d before Enrollment*	*≥7d before Day 1/ Dose 1*	*1*			*3*			*8*			*11*			*14*			*15*	*21*	*28*	
Procedure	*Screening*	*Enrollment/ Baseline*	*Pre-dose*		*Post-dose*	*Pre-dose*		*Post-dose*	*Pre-dose*		*Post-dose*	*Pre-dose*		*Post-dose*	*Pre-dose*		*Post-dose*		*Safety*	*Closing*	
Informed consent	X																				
Assign ID number	X																				
Record/confirm locator information	X	X	X			X			X			X			X			X	X	X	X
Demographics	X																				
Assess eligibility	X	X																			
Randomize to treatment group and time point		X																			
Medical history	X																				
Medication history/ concomitant meds	X	*	*	Dose #1 in clinic		*	Dose #3 in clinic		*	Dose #8 in clinic		*	Dose #11 in clinic		*	Dose #14 in clinic		*	*	*	*
HIV-1 & confirmatory test	X																				
HSV-1 and HSV-2 test	X																				
Urine pregnancy test	X	X	X			*			X			*			X				X	X	*
Vital signs§	X	X	X		X	X		X	X		X	X		X	X		X	X	X	*	*
Physical exam***	X	*	*		*	*		*	*		*	*		*	*		*	*	X	*	*
Clinical labs (hematology, chemistry, coagulation)	X	*	X		*	X		*	X		*	X		*	X		*	X	X	*	*
Urinalysis	X	*	*		*	*		*	*		*	*		*	*		*	*	X	*	*
Urine culture	*	*	*		*	*		*	*		*	*		*	*		*	*	*	*	*
Pelvic exam (with breast exam at screening)	X	X	X		*	*	Dose #3	*	X	Dose #8 in	*	*		*	*	Dose #14	*	X	X	*	*
NAAT for NG/CT/TV	X																				

Visit Number		Visit 0	Visit 1	Visit 2			Visit 3			Visit 4			Visit 5			Visit 6			Visit 7	Visit 8	Visit 9	Unscheduled Visit
Cycle Day			*21-28 (not on HC) 15-22 (if on HC)*	*7-10 (not on HC) 1-4 (if on HC)*																		
Study Day		*≤45d before Enrollment*	*≥7d before Day 1/ Dose 1*	*1*			*3*			*8*			*11*			*14*			*15*	*21*	*28*	
Procedure		*Screening*	*Enrollment/ Baseline*	*Pre-dose*		*Post-dose*	*Pre-dose*		*Post-dose*	*Pre-dose*		*Post-dose*	*Pre-dose*		*Post-dose*	*Pre-dose*		*Post-dose*		*Safety*	*Closing*	
Swab for RSID test			X																			
Pap smear**		*			Dose #1 in clinic									Dose #11 in clinic								
Vaginal swab for wet mount		*	*	*		*	*		*	*		*	*		*	*		*	*	*	*	*
Vaginal strip for pH		*	*	*		*	*		*	*		*	*		*	*		*	*	*	*	*
Treat or prescribe treatment for RTI/STI		*	*	*			*			*			*			*			*	*	*	*
Counseling: (HIV pre-/post-test/ risk reduction); adherence; contraception		X	X	*		X	*		X	*		X	*		X	*		X	X	X	*	*
Collect used applicators for DSA testing						X			X			X			X			X				*
Distribute applicators, bags & labels						X			X			X			X							*
Collect applicators used at home							X			X			X			X						*
**1º End-point: Safety**	AEs		X	X		X	X		X	X		X	X		X	X		X	X	X	X	*
	Biopsies (1 vaginal 1 cervical)		X																X			
**1º End-point: PK**	Blood			X		X (4 OR 8h post-dose 1)	X			X			X	Dose #11		X	Dose #14 in clinic	X (0.5,1,2,4,6,8h post-dose 14)	X (24h post dose 14)			
**Expl: API concen-tration, PD,**	CVL			X		X (4 or 8h post-dose 1)													X			

Visit Number		Visit 0	Visit 1	Visit 2			Visit 3			Visit 4			Visit 5			Visit 6			Visit 7	Visit 8	Visit 9	Unsche-duled Visit
Cycle Day			*21-28 (not on HC) 15-22 (if on HC)*	*7-10 (not on HC) 1-4 (if on HC)*																		
Study Day		*≤45d before Enrollment*	*≥7d before Day 1/ Dose 1*	*1*			*3*			*8*			*11*			*14*			*15*	*21*	*28*	
Procedure		*Screening*	*Enrollment/ Baseline*	*Pre-dose*		*Post-dose*	*Pre-dose*		*Post-dose*	*Pre-dose*		*Post-dose*	*Pre-dose*		*Post-dose*	*Pre-dose*		*Post-dose*		*Safety*	*Closing*	
**genital tract immune mediators E. coli and RSID**																						
**Explor: ADA**	Blood			X												X				X	X	
**Explor: Gene expres-sion in tissues**	Cervical Biopsy		X		**Dose #1 in clinic**														X			
**Explor: vaginal micro-biome**	Swab			X															X			

* = if indicated; ** If no appropriately documented history of Pap test and completed follow-up of any abnormal Pap tests consistent with ACOG bulletins #140 and #168.

***Complete physical exam at screening and Visit 8 (Day 21); targeted exam at other visits, only if clinically indicated. §Height only at screening; weight at screening and Visit 8/Day 21

Appendix 3. Documentation of Informed Consent for OL Safety Run-In

ALBERT EINSTEIN COLLEGE OF MEDICINE
MONTEFIORE MEDICAL CENTER

DOCUMENTATION OF INFORMED CONSENT AND HIPAA AUTHORIZATION

Introduction

You are being asked to participate in a research study called ***A Phase 1 Trial to Evaluate the Safety, Pharmacokinetics (PK) and Pharmacodynamics (PD) of PC-6500 (Griffithsin [GRFT] in a Carrageenan Gel) in Healthy Women.*** Your participation is voluntary — it is up to you whether you would like to participate. It is fine to say “no” now or at any time after you have started the study. If you say “no,” your decision will not affect any of your rights or benefits or your access to care.

The researcher in charge of this project is called the “Principal Investigator.” Her name is Dr. Marla Keller. You can reach Dr. Keller at:

Albert Einstein College of Medicine
1300 Morris Park Avenue
Block Building, Room 512
Bronx, NY 10461
718-430-3240

For questions about the research study, or if you believe you have an injury, contact the Principal Investigator or the IRB.

Support for this research study is provided by: The Population Council through a grant from the US Agency for International Development (USAID).

The Institutional Review Boards (IRBs) of the Population Council and the Albert Einstein College of Medicine and Montefiore Medical Center have approved this research study. The Einstein IRB # is in the stamp in the upper right hand corner. If you have questions regarding your rights as a research subject you may contact Einstein’s IRB at 718-430-2253 or by mail:

Einstein IRB
Albert Einstein College of Medicine
1300 Morris Park Ave., Belfer Bldg. #1002
Bronx, New York 10461

Why is this study being done?

This study involves research about microbicides. A microbicide is a substance that women can insert into the vagina to prevent HIV (human immunodeficiency virus) infection and other sexually transmitted infections (STIs). This study is testing a new microbicide being developed that contains griffithsin (GRFT) in a carrageenan (CG) gel, also called “PC-6500.” The main purpose of this study is to test the safety and pharmacokinetics (what the body does to the drug) of PC-6500. The study will also explore how PC-6500 works in the body (pharmacodynamics).

GRFT is a protein that comes from a type of algae found in the ocean. In laboratory testing and in animal studies, scientists have shown that GRFT is very effective in preventing HIV. However, GRFT has not yet been tested to see if it is safe or effective for preventing HIV in people.

For this study, GRFT has been formulated in a carrageenan gel. Carrageenans come from seaweed and are used in many foods and cosmetics, including toothpaste and baby formula. Carrageenan is Generally Recognized as Safe (GRAS) by the U.S. Food and Drug Administration (FDA) for use in foods and on the skin.

PC-6500 (GRFT in a carrageenan gel) is not approved by the FDA. This means that PC-6500 is an experimental product that can only be used in research studies. Your participation in the study will help us to learn about the safety of PC-6500 in women. **This is the first time PC-6500 is being tested in people.**

This is a Phase 1 study, which means researchers are testing PC-6500 in a small group of women to make sure it is safe for vaginal use and to find out if it causes any side effects. The products being used in this study are supplied by the Population Council, a non-profit organization based in New York.

Why am I being asked to participate?

You are being asked to participate because you have expressed an interest in being in this study. You will be screened by the study staff members to make sure that you are eligible to participate in this study.

You may qualify to take part in this study if you meet the following requirements:

- Are 18-49 years old.
- Are HIV negative.
- Are not pregnant.
- Have no major illnesses, including hepatitis.
- Are not taking any medicines that are not allowed while taking part in this study.
- Are using a combined oral contraceptive pill or the contraceptive patch as your method of birth control during the study.
- Have a regular menstrual period.
- Have someone who can stay with you overnight after you receive your first dose (Day 1) until you return to the clinic the following morning (Day 2).
- Are willing to abstain from sex during the study.
- Agree not to be in any other clinical studies while taking part in this study.
- Agree to undergo all study procedures, follow all instructions and come to all study visits.

We want you to know what the study is about before you decide if you want to join. This consent form gives you information about the study. Once you read this form, and if you agree to take part in the study, you will be asked to sign your name on this form. You will be given a copy of this consent form to keep. **However, signing this consent form does not guarantee you will be in the study, as you may not qualify.**

The study staff will talk with you about it and answer your questions.

<u>How many people will take part in the research study?</u>

If you are eligible and choose to take part, you will be one of 7 women in this study. There will be only one study site for this study, the Albert Einstein College of Medicine.

<u>How long will I take part in this research?</u>

You will have 4 scheduled visits, including screening. The screening visit will be up to 30 days before you enroll in the study. Once we have made sure you are eligible and if you choose to enroll, you will be in the study for 8 days. Your total study participation will be about 40 days, including screening.

**VISIT 0**		**VISIT 1**		**VISIT 2**		**VISIT 3**
**Screening**	→	**Enrollment**<br>Single Dose	→	**Follow-up**	→	**Exit**
≤ 30 days prior to Enrollment		**Day 1**		**Day 2**		**Day 8**

<u>What will happen if I participate in the study?</u>

Screening Visit – Visit 0 (up to 30 days before enrollment):

The screening visit will take about 2 hours.

- The purpose of this visit is to make sure that you understand what the study is about and that you are willing and eligible to take part in the study.
- A member of the study team will talk to you about the study, review this informed consent form with you and answer your questions. You will be asked if you want to take part in the study. If you choose to take part, you will be asked to sign this informed consent form and you will be given a copy.
- If you take part in this study, you will be required to:
  - **Remain sexually abstinent for about 10 days – starting 2 days before your enrollment visit through your last study visit.** For this study, being sexually abstinent includes not having vaginal or anal sex, not participating in receptive oral or digital (finger) sex, and not masturbating (either with fingers or sex toys), or any other type of sexual activity not mentioned here.
  - **Use combined oral contraception (pills) or a contraceptive patch as your method of birth control for the entire time you are in the study.** The clinician will ask you about your birth control method and will tell you if your method of birth control is allowed for this trial. If your birth control method is not allowed, you will not be eligible to take part in this study.
- You will be asked some questions about your health and you will be given a complete physical exam. Your temperature, height, weight, blood pressure, and pulse will be taken. You will also give a urine sample to check your health and to see if you are pregnant. If you are pregnant, you will not be able to take part in this study.
- A member of the clinic staff will take about 2-3 tablespoons of blood for laboratory tests to check your health and to test for HIV and herpes simplex virus infection.

  - If you test positive for HIV, you will receive appropriate counseling and will be referred for treatment if necessary. If you are HIV-positive you will not be able to take part in this study, and no more blood will be taken from you.
  - If you have genital herpes virus infection without any symptoms, you may still take part in this study.
- You will have a pelvic exam to check to see if your vagina and cervix are healthy. The clinician will check inside your vagina (using a plastic instrument called a speculum to see inside the vagina). During the exam, swabs will be used to take samples from your vagina and cervix to test for sexually transmitted infections (STIs), including Gonorrhea, Chlamydia, and Trichomonas.
  - If you test positive for Gonorrhea, Chlamydia, or Trichomonas, you will receive appropriate counseling and will be referred for treatment. You will not be able to take part in this study.
  - If you test positive for other reproductive tract infections (bacterial vaginosis [BV] or candidiasis [yeast infection]), or if you have a urinary tract infection (UTI), you will be given treatment and can be rescreened for the study after you have completed treatment.
- If you require a Pap test, cells from your cervix will be collected using a small brush and sent to a lab. If abnormal cells are found in your cervix, you will not be able to take part in this study. You will be referred for follow up.
- You will be asked to answer a few questions about your background, including your age, marital status, education, race, and ethnicity.
- You will be asked not to use any products inside the vagina from 2 days before the next visit (enrollment) until you complete the study.
- You will be reminded not to engage in sexual activity starting 48 hours (2 days) before enrollment.

Enrollment Visit – Visit #1 (Study Day 1):

This visit will take about 13 hours.

- We will review the results of the lab tests done at the Screening Visit. If you are eligible based on your lab test results, and if you choose to enroll, you will continue. If you are ineligible, you will receive any necessary treatment and/or referrals and your participation will end.
- You will be asked some questions about your health since your last visit, and you will be given a physical exam, if needed.
- Your temperature, weight, blood pressure, and pulse will be taken. You will also give a urine sample to check your health and to see if you are pregnant. If you are pregnant, you will not be able to take part in this study.
- You will have an electrocardiogram (EKG) test to examine your heart rhythms. This is done by attaching several electrodes to the skin on your chest and each arm and leg. The electrodes are attached to a machine that will measure the electrical activity of your heart, which will be reviewed by a doctor.
- A member of the clinic staff will take about 2 tablespoons of blood for lab tests to check your health before you receive your dose of gel and again at the end of your visit.
- You will have a pelvic exam using a speculum. If you have symptoms, swabs will be used to take samples from your vagina and cervix to test for infection. If you have an infection, you will be treated or referred for treatment and may not be eligible to participate.
- Before your dose is inserted, you will have a saline solution squirted into your vagina to collect a specimen called a “lavage” that will be sent to the lab for testing. A swab will also be used to take a sample of the fluid from your vagina. These specimens will help scientists to learn more about how PC-6500 gel works in the body.

- After the pelvic exam, the clinician will use a plastic applicator to insert a single dose of PC-6500 gel into your vagina.
- You will have blood drawn 10 times during your visit to see how PC-6500 is affected by the body and how long it can be measured in the blood after dosing. To use only one needle stick for all of the blood draws, you may choose to have a small tube (a saline lock) put into a vein in your arm for the first blood draw that will be left in place during your stay in the clinic; it will be removed once all blood draws have been completed. You may also choose to have individual needle sticks for each blood draw. During each blood draw, approximately 2 teaspoons of blood will be taken. The first blood draw will be before the gel is inserted. After the dose has been inserted, you will have additional blood draws as follows:

  - 30 minutes after the dose
  - 1 hour after the dose
  - 2 hours after the dose
  - 3 hours after the dose
  - 4 hours after the dose
  - 6 hours after the dose
  - 8 hours after the dose
  - 10 hours after the dose
  - 12 hours after the dose

- While you are at the clinic, your vital signs will be taken periodically to see how your body is responding to the gel.
- You will be given meals and snacks while you are at the clinic.
- You may have a physical exam before leaving the clinic, if the clinician thinks it is necessary.
- You will be able to return home after the clinician has checked that you have tolerated your dose of gel.
- You will be asked to call the clinic when you arrive home; if you do not call the clinic, a study staff member will call you to make sure you are alright.

Visit #2 (Study Day 2):

This visit will take about 1-2 hours.

- You will be asked some questions about your health since your last visit.
- You may be given a physical exam if one is needed.
- Your temperature, blood pressure, and pulse will be taken. You may also be asked to give a urine sample to check your health.
- You will have an EKG.
- A member of the clinic staff will take about 2 tablespoons of blood for lab tests.
- You will have a pelvic exam with a speculum to check your vagina and cervix for any changes after the gel was inserted. A swab will be used to take a sample of the fluid from your vagina. You will have a lavage (saline solution squirted into your vagina) that will be sent to the laboratory for testing. These tests will help researchers understand how the gel affects your body.

Closing Visit – Visit #3 (Study Day 8):

This visit will take about 2 hours and will be your last study visit.

- This visit will take place 7 days after your dose of gel was inserted.
- You will be asked some questions about your health since your last visit and you will be given a physical exam, like at the screening visit. Your temperature, weight, blood pressure, and pulse will be taken. You will also give a urine sample to check your health and to see if you are pregnant.
- A member of the clinic staff will take 2 tablespoons of blood for lab tests to check your health and for testing to learn more about the gel.
- You will have a pelvic exam. During the pelvic exam, a nurse or doctor will check inside your vagina (with a speculum), to see if you have any changes in your vagina following gel use. If you have any symptoms, samples will be taken from your vagina and cervix for testing. If you have an infection, you will be treated or referred.

Unscheduled Visits:

It may be necessary for you to make additional visits and complete additional procedures. This could happen if any of the study procedures listed above need to be repeated (for example, if there are unexpected abnormal results); if there are difficulties in sample shipping, processing, or testing; and/or if you are having any symptoms or changes in your physical condition.

A description of this clinical trial is available on www.ClinicalTrials.gov, as required by U.S. law. This Web site will include a summary of the results, but will not include information that can identify you. You can search this Web site at any time.

Will there be testing for HIV?

Yes, HIV testing will be done during this research study. The following is important information about HIV, HIV testing, and your test results:

- HIV causes AIDS and can be spread through sexual activity, sharing needles, by pregnant women to their fetuses, and through breastfeeding infants.
- There is treatment for HIV that can help you stay healthy.
- People with HIV or AIDS should adopt practices to protect people in their lives from becoming infected with HIV.
- HIV testing is voluntary and can be done anonymously at a public testing center. However, testing is required if you want to be in this research study.
- The law protects the confidentiality of HIV-related test results.
- The law prohibits discrimination based on your HIV status and services are available to address any discrimination.
- If as a result of being screened for this study you are INITIALLY diagnosed with HIV, the results must be reported to the New York State Department of Health for contact tracing purposes.
- If as a result of being screened for this study you are diagnosed with HIV, you will be given HIV counseling or a referral for HIV counseling.

Genetic testing

This study will not involve genetic research or genetic testing.

Specimen Banking (Future Use and Storage)

We will store your specimens and information about you in a “biobank,” which is a library of information and specimens (tissue and blood) from many studies. These specimens and information can be linked to you. In the future, researchers can apply for permission to use the specimens and information for new studies to prevent, diagnose or treat disease, including genetic research. If you agree to the future use, some of your de-identified genetic and health information (not linked to you) may be placed into one or more scientific databases. These may include databases maintained by the federal government. Your specimens and information may be kept for a long time, perhaps longer than 50 years. You may remove your consent for future research at any time by contacting the Principal Investigator named on the first page of this consent form or the IRB office at 718-430-2237. If you do, we will destroy remaining specimens and information, but if these were already shared with other researchers, we cannot get them back.

You can choose not to participate in the biobank and still be part of the main study and this will not affect your treatment at this facility.

INITIAL ONE (1) OF THE FOLLOWING OPTIONS
_______ I consent to have my specimens and information about me used for future research studies.
_______ I do NOT consent to have my specimens and information about me used for future research studies. The specimens and information will be destroyed at the end of the study.

Information Banking (Future Use and Storage)

We will store information about you in a “bank,” which is a library of information from many studies. This information can be linked to you. In the future, researchers can apply for permission to use the information for new studies to prevent, diagnose or treat disease, including genetic research. If you agree to the future use, some of your de-identified genetic and health information (not linked to you) may be placed into one or more scientific databases. These may include databases maintained by the federal government. Your information may be kept for a long time, perhaps longer than 50 years. You may remove your consent for future research at any time by contacting the Principal Investigator named on the first page of the consent form or the IRB office at 718-430-2237. If you do, we will destroy the information in the bank, but if the information was already shared with other researchers, we cannot get it back.

You can choose not to participate in the bank and still be part of the main study and this will not affect your treatment at this facility.

INITIAL ONE (1) OF THE FOLLOWING OPTIONS
_______ I consent to have my information used for future research studies.
_______ I do NOT consent to have my information used for future research studies. The information will be destroyed at the end of the study.

INITIAL YOUR CHOICE BELOW
I consent to be contacted in the future to learn about:
_______ New research protocols that I may wish to join.

_______ General information about research findings.
_______ I do not want to be contacted at all.

Will I be paid for being in this research study?

You will receive a total of $400 if you complete all 4 visits, including screening. You will be compensated $50 for screening, $250 for Visit 1, $50 for Visit 2, and $50 for Visit 3. You will receive your reimbursement through a pre-loaded debit card called a ClinCard, which you must keep for the entire study. If you choose to withdraw from the study before all visits are completed, you will be paid only for the visits you completed. You will be compensated at the end of each visit you complete. This compensation is for your time, food, and travel related to participating in the study.

Some researchers may develop tests, treatments or products that are worth money. You will not receive payment of any kind for your specimens and information or for any tests, treatments, products or other things of value that may result from the research.

Will it cost me anything to participate in this study?

There will be no cost to you to participate in the study. However, we will not compensate for travel costs, missing work and/or childcare.

Taking part in this study will not involve added costs to you. All study drugs will be given free of charge by the Population Council.

What will happen if I am injured because I took part in this study?

If you are injured as a result of this research, only immediate, essential, short-term medical treatment, as determined by the participating hospital, will be available for the injury without charge to you personally.

- No monetary compensation will be offered.
- You are not waiving any of your legal rights by signing this informed consent document.
- If additional treatment is required as a result of a physical injury related to the research, necessary medical treatment will be provided to you and billed to your insurance company or to you as part of your medical expenses.

Immediately report any discomforts, problems or injuries you experience during the course of your participation in the study to Dr. Marla Keller at (718) 430-3240.

What else do I have to do?

- You must tell the research study doctor about any past and present diseases or allergies you are aware of and about all medications you are taking, including “over-the-counter” remedies and nutritional supplements or herbs.
- If you do not feel well at any time, call your doctor or the research study doctor immediately.
- ***Some drugs may cause a reaction that, if not treated promptly, could be life-threatening. It is important that you report all symptoms, reactions and other complaints to the research study doctor.***

- If you think you have become pregnant, contact your research study doctor immediately.
- If any other doctor recommends that you take any medicine, please inform him/her that you are taking part in a research study. You should give the other doctor the research study doctor's name and phone number.
- You may carry out all your normal daily activities, except vaginal product use and sexual activity, as restricted (abstinence from 48 hours before enrollment through Day 8).

Confidentiality

We will keep your information confidential. Your research records will be kept confidential and your name will not be used in any written or verbal reports. Your information will be given a code number and separated from your name or any other information that could identify you. The form that links your name to the code number will be kept in a secure manner and only the Investigator and study staff will have access to the file. All information will be kept in a secure manner and computer records will be password protected. Your study information and specimens will be kept as long as they are useful for this research.

Medical information collected during the research, such as test results, may be entered into your Montefiore electronic medical record and will be available to clinicians and other staff at Montefiore who provide care to you.

The only people who can see your research records are:

- the research team and staff who work with them
- clinicians and staff at Albert Einstein/Montefiore who review your records for your care and individuals who are authorized to monitor or audit the research
- Population Council study staff and monitors hired by the Population Council
- groups that review research (the Einstein IRB, Population Council IRB, the Office for Human Research Protections, and the US FDA)

The people who receive your health information may not be required by privacy laws to protect it and may share your information with others without your permission, if permitted by laws governing them. All of these groups have been asked to keep your information confidential.

Are there any risks to me?

GRFT (PC-6500)
It is not expected that this trial will expose you to any unreasonable risk. However, as this is the first time GRFT is being tested in humans, there may be risks that we are unaware of. Some women who have used other vaginal products have experienced genital irritation during or after use. Since this is the first time GRFT is being used vaginally, it is not known whether or not the gel will cause any irritation or discomfort.

Blood draws
Blood draws may lead to excessive bleeding, discomfort, feelings of dizziness or faintness, and/or bruising, swelling and/or infection. Rarely, the vein where we inserted the needle will become sore or red. Sometimes, a temporary harmless "black and blue" mark may develop. Very rarely, fainting may occur.

Pelvic exams

The pelvic exams during the study may cause you mild discomfort and/or embarrassment. When the study staff takes pelvic specimens, this may cause spotting during or after the pelvic exam. Disclosure of HIV and STI status may cause worry, sadness or depression.

Abstinence

If you do not follow the study instructions and engage in sexual intercourse during the study (48 hours before enrollment through Day 8), and if you do not use a condom, you may be at risk of getting a sexually transmitted infection, including HIV.

Risks to women who are or may become pregnant

The effect of GRFT on an embryo or fetus (developing baby still in the womb), or on a breastfeeding infant, is unknown and may be harmful. Because of these unknown risks, women cannot take part in this study if they are:

- Pregnant
- Trying to become pregnant
- Breastfeeding or sharing breast milk

If you are sexually active and able to become pregnant, you must agree to use one of the birth control methods listed below. You must use birth control for the duration of the study. If you miss a period, or think you might be pregnant during the study, you must tell the study doctor immediately.

Acceptable birth control methods for use in this study are:

- Birth control pills or patches

Birth control methods that are not allowed:

- Any other hormonal method (injections or implants)
- Barrier methods (such as a condom or diaphragm) used with a spermicide (a foam, cream, or gel that kills sperm)
- NuvaRing®
- Sterilization
- Intrauterine device or intrauterine system

Allergic reaction to study drug

Any drug can cause an allergic reaction, which could be mild or more serious and can even result in death. Common symptoms of an allergic reaction are rash, itching, skin problems, swelling of the face and throat, or trouble breathing. If you are having trouble breathing, call 911 immediately.

New findings

If we learn any significant new findings during the study that might influence your decision to participate, we will contact you and explain them.

Unknown risks

We have described all the risks we know. However, because this is research, there a possibility that you or an embryo or fetus will have a reaction that we do not know about yet and is not expected. If we learn

about other risks, we will let you know what they are so that you can decide whether or not you want to continue to be in the study.

Taking study drug with other medications
The following medications are not allowed during the course of the study:

- Vaginal and rectal products (including, but not limited to vaginal medications, spermicidal products, lubricants, douches)
- Antiretroviral therapy for prevention or treatment
- HSV treatment: Acyclovir, valacyclovir or other medication
- Systemic steroids
- Systemic antibiotics
- Antihypertensive medications, including diuretics
- Investigational therapies for any medical condition

Taking these drugs and PC-6500 together may cause side effects or impact the study in a negative way.

For your safety during this study, call your study doctor BEFORE you take any:

- New medications prescribed by your doctor
- Other medications sold over-the-counter without a prescription
- Dietary or herbal supplements

Are there possible benefits to me?
You will not experience any direct benefit personally from participating in this study. An indirect benefit of taking part in this study is that you are helping researchers gain knowledge and understanding of a new microbicide (drug that may prevent infection from HIV) and that a new microbicide may help others in the future.

What choices do I have other than participating in this study?
You can choose not to participate in the study.

Are there any consequences to me if I decide to stop participating in this study?
No. If you decide to take part, you are free to stop participating at any time without giving a reason. However, some of the information may have already been entered into the study and that will not be removed. The researchers and the sponsor may continue to use and share the information they have already collected.

To revoke (take back) your consent and authorization, you must contact the Principal Investigator in writing at the address on page 1 of this form. However, you may first call or speak to the Principal Investigator and she will stop collecting new information about you. If you take back your consent and authorization, you will not be allowed to continue to participate in this research study.

If you decide to stop taking part in the study for any reason, we will ask you to make a final study visit to make sure you are alright.

The final study visit will take about ½ hour.

At this visit we will:

- check your blood pressure, temperature, weight and pulse
- perform a pelvic and/or physical exam, if needed

Can the study end my participation early?

It is possible that you may be removed from the study without your consent in the event of any of the following:

- If you have an adverse reaction or side effect to PC-6500 gel.
- If you need a treatment that is not allowed in this study.
- If you do not keep appointments.
- If continuing the study is harmful to you.
- If you become pregnant.
- If the study is canceled or ends early.

Your responsibilities as a research volunteer are as follows:

- To attend each study visit as scheduled.
- To contact the study clinic if you have any problems or cannot attend a visit.
- To ask any questions when you do not understand something that has been said.
- To tell the study staff, nurse or doctor if you do not feel well or if you have any side effects, even if you do not think they are important.

CONSENT TO PARTICIPATE

I have read the consent form and I understand that it is up to me whether or not I participate. I know enough about the purpose, methods, risks and benefits of the research study to decide that I want to take part in it. I understand that I am not waiving any of my legal rights by signing this informed consent document. I will be given a signed copy of this consent form.

Printed name of participant	Signature of participant	Date	Time
Printed name of the person conducting the consent process	Signature	Date	Time

Appendix 4. Documentation of Informed Consent for Randomized Period

ALBERT EINSTEIN COLLEGE OF MEDICINE
MONTEFIORE MEDICAL CENTER

DOCUMENTATION OF INFORMED CONSENT AND HIPAA AUTHORIZATION

Introduction

You are being asked to participate in a research study called ***A Phase 1 Trial to Evaluate the Safety, Pharmacokinetics (PK) and Pharmacodynamics (PD) of PC-6500 (Griffithsin [GRFT] in a Carrageenan Gel) in Healthy Women.*** Your participation is voluntary -- it is up to you whether you would like to participate. It is fine to say “no” now or at any time after you have started the study. If you say “no,” your decision will not affect any of your rights or benefits or your access to care.

The researcher in charge of this project is called the “Principal Investigator.” Her name is Dr. Marla Keller. You can reach Dr. Keller at:

Albert Einstein College of Medicine
1300 Morris Park Avenue
Block Building, Room 512
Bronx, NY 10461
718-430-3240

For questions about the research study, or if you believe you have an injury, contact the Principal Investigator or the IRB.

Support for this research study is provided by: The Population Council through a grant from the US Agency for International Development (USAID).

The Institutional Review Boards (IRBs) of the Population Council and of the Albert Einstein College of Medicine and Montefiore Medical Center have approved this research study. The Einstein IRB # is in the stamp in the upper right hand corner. If you have questions regarding your rights as a research subject you may contact Einstein’s IRB office at 718-430-2253 or by mail:

Einstein IRB
Albert Einstein College of Medicine
1300 Morris Park Ave., Belfer Bldg. #1002
Bronx, New York 10461

Why is this study being done?

This study involves research about microbicides. A microbicide is a substance that women can insert into the vagina to prevent HIV (human immunodeficiency virus) infection and other sexually transmitted infections (STIs). This study is testing a new microbicide gel being developed that contains griffithsin (GRFT) in a carrageenan (CG) gel, also called “PC-6500.” The main purpose of this study is to test the safety and pharmacokinetics (what the body does to the drug) of PC-6500. The study will also explore how PC-6500 works in the body (pharmacodynamics).

GRFT is a protein that comes from a type of algae found in the ocean. In laboratory testing and in animal studies, scientists have shown that GRFT is very effective in preventing HIV. However, GRFT has not yet been tested to see if it is safe or effective for preventing HIV in people.

For this study, GRFT has been formulated in a carrageenan gel. Carrageenans come from seaweed and are used in many foods and cosmetics, including toothpaste and baby formula. Carrageenan is Generally Recognized as Safe (GRAS) by the U.S. Food and Drug Administration (FDA) for use in foods and on the skin.

PC-6500 (GRFT in a carrageenan gel) is not approved by the FDA. This means that PC-6500 is an experimental product that can only be used in research studies. Your participation in the study will help us to learn about the safety of PC-6500 in women. PC-6500 has been tested in 7 women who each received a single dose of gel under close observation. None of the women experienced any problems. PC-6500 is now being tested in 20 women here at Albert Einstein College of Medicine.

This is a Phase 1 study, which means researchers are testing PC-6500 in a small group of women to make sure it is safe for vaginal use and to find out if it causes any side effects. The products used in this study are supplied by the Population Council, a non-profit organization based in New York.

Why am I being asked to participate?

You are being asked to participate because you have expressed an interest in being in this study. You will be screened by the study staff members to make sure that you are eligible to participate in this study.

You may qualify to take part in this study if you:

- Are 18-49 years old.
- Are HIV negative.
- Are not pregnant.
- Have no major illnesses, including hepatitis.
- Are not taking any medicines that are not allowed while taking part in this study.
- Are willing to abstain from sexual intercourse (not having sex) or any sexual activity during the study.
- Are taking an effective method of birth control during the study.
- Have a regular menstrual period.
- Agree to not be in any other clinical studies while taking part in this study.
- Agree to undergo all study procedures, follow all instructions and come to all study visits.

We want you to know what the study is about before you decide if you want to join. This consent form gives you information about the study. Once you read this form, and if you agree to take part in the study, you will be asked to sign your name on this form. You will be given a copy of this consent form to keep. **However, signing this consent form does not guarantee you will be in the study, as you may not qualify.**

The study staff will talk with you about it and answer your questions.

How many people will take part in the research study?

If you are eligible and choose to take part, you will be one of 20 women in this study. There will be only one study site for this study, the Albert Einstein College of Medicine.

How long will I take part in this research?

You will have 10 scheduled visits, including screening. The screening visit will take place up to 45 days before you enroll in the study. If you are eligible and choose to enroll, you will have 9 additional visits. You will be in the study for 35-45 days, depending on how long your menstrual period lasts. Your total study participation will be up to 80-90 days, including screening.

**Visit 0 Screen-ing**	→	**Visit 1 Enroll-ment**	→	**Visit 2**	→	**Visit 3**	→	**Visit 4**	→	**Visit 5**	→	**Visit 6**	→	**Visit 7**	→	**Visit 8**	→	**Visit 9**
Up to 45 days before enroll-ment		*7 days before Dose 1 (biopsy)*		*Day 1 First dose*		*Day 3 Follow-up*		*Day 8 Follow-up (Pelvic exam)*		*Day 11 Follow-up*		*Day 14 Last dose*		*Day 15 (biopsy)*		*Day 21 Final safety visit*		*Day 28 Clos-ing*

What will happen if I participate in the study?

If you are eligible and you choose to enroll in this trial, you will be randomly assigned to 1 of 2 study groups: PC-6500 gel (carrageenan gel with griffithsin) or to a placebo gel called PC-535, which is carrageenan gel without the griffithsin in it. You will be assigned to a group by random chance (like flipping a coin). Carrageenan gels have been used in many microbicide trials by thousands of women (and men) and have been found to be safe, but have not been found to prevent HIV. Up to 14 women will be assigned to use PC-6500 and up to 6 women to the placebo (PC-535) gel. Neither you nor the study staff will know which gel you are using, and both gels look the same. No matter which gel you are using, you will follow the same procedures and have the same care.

Screening Visit – Visit 0 (up to 45 days before enrollment)

The screening visit will take about 2 hours.

- The purpose of this visit is to make sure that you understand what the study is about and that you are willing and eligible to take part in the study.
- A member of the study team will talk to you about the study, review this informed consent form with you and answer your questions. You will be asked if you want to take part in the study. If you choose to take part, you will be asked to sign this informed consent form and you will be given a copy.
- If you take part in this study, you will be required to:
  - **Remain sexually abstinent for about 28 days – starting 4 days before your enrollment visit (Visit 1) through Day 17 (3 days after your last dose).** For this study, being sexually abstinent includes not having vaginal or anal sex, not participating in receptive oral or digital (finger) sex, and not masturbating (either with fingers or sex toys), or any other type of sexual activity not mentioned here.
  - **Use an effective method of birth control for the entire time you are in the study.** The study clinician will ask you about your birth control method and will tell you if your method is allowed for this trial. If your birth control method is not allowed, you will not be eligible to take part in this study.

- A member of the clinic staff will take about 2-3 tablespoons of blood for laboratory tests to check your health and to test for HIV and herpes simplex virus infection.
  - If you test positive for HIV, you will receive appropriate counseling and will be referred for treatment if necessary. If you are HIV-positive you will not be able to take part in this study, and no more blood will be taken from you.
  - If you have genital herpes virus infection without any symptoms, you may still take part in this study.
- You will be asked some questions about your health and you will be given a complete physical exam (like the one you receive from your personal doctor). Your temperature, height, weight, blood pressure, and pulse will be taken. You will also give a urine sample to check your health and to see if you are pregnant.
  - If you are pregnant, you will not be able to take part in this study.
- You will have a pelvic exam to check to see if your vagina and cervix are healthy. The clinician will check inside your vagina (using a plastic instrument called a speculum to see inside the vagina). During the exam, swabs will be used to take samples from your vagina and cervix to test for sexually transmitted infections (STIs), including Gonorrhea, Chlamydia and Trichomonas.
  - If you test positive for Gonorrhea, Chlamydia, or Trichomonas, you will receive appropriate counseling and will be referred for treatment. You will not be able to take part in this study.
  - If you test positive for other reproductive tract infections (bacterial vaginosis [BV] or candidiasis [yeast infection]), or if you have a urinary tract infection (UTI), you will be given treatment and can be rescreened for the study after you have completed treatment.
- If you require a Pap test, cells from your cervix will be collected using a small brush and sent to a lab.
  - If abnormal cells are found in your cervix, you will not be able to take part in this study. You will be referred for follow-up.
- You will be asked to answer a few questions about your background, including your age, marital status, education, race, and ethnicity.
- You will be asked not to use any products inside the vagina from 2 days before your enrollment visit (Visit 1) through Visit 8 (Day 21 Safety Visit).
- You will be reminded not to have sex starting 96 hours (4 days) before enrollment through Day 17 (3 days after your last dose).

Enrollment Visit – Visit #1:

This visit will take about 2 hours.

- We will review the results of the lab tests done at the Screening Visit. If you are eligible based on your lab test results, and if you choose to enroll, you will continue. If you are ineligible, you will receive any necessary treatment and/or referrals and your participation will end.
- You will be asked some questions about your health since your last visit, and you will be given a physical exam, if needed.
- Your temperature, weight, blood pressure, and pulse will be taken. You will also give a urine sample to check your health and to see if you are pregnant. If you are pregnant, you will not be able to take part in this study.
- A member of the clinic staff will take about 2 tablespoons of blood for lab tests.

- You will have a pelvic exam using a speculum. If you have any symptoms, swabs will be used to take samples from your vagina and cervix for testing. If you have an infection, you will be treated or referred for treatment and may not be eligible to participate.
  - During this exam, 3 small samples of tissue will be taken (2 from your cervix, 1 from your vagina) for testing to see what happens to PC-6500 in the body. This procedure is called a biopsy. The small piece of tissue is taken with special forceps (similar to tweezers). You may feel a pinch when the tissue is taken. During the biopsy, you may be asked to cough just as the sample is being taken to reduce or relieve any pain or soreness you may have when the tissue sample is taken. The areas where the biopsies will be taken will be gently cleaned with saline before the biopsies are taken. A gel (benzocaine) can be put on the biopsy areas to numb them. You may also take Tylenol® up to one hour before the procedure if you wish. Instructions on how to care for your biopsies will be reviewed.
- A swab will be taken from your vagina for testing to make sure there is no semen present. If semen is found (indicating you have had sexual intercourse), it will mean we cannot do the necessary tests to find out how the gel is working in your body.
- Although you will not begin gel use until your next visit, you will be randomly assigned to use PC-6500 gel or PC-535 placebo. You will also be randomly assigned to 1 of 2 time points at Visit 2 (either 4 hours or 8 hours after your first dose of gel) when you will have a blood sample and some vaginal fluid collected.
- You will be scheduled to return to the clinic one week later for Visit 2.

Visit #2 (Day 1)

This visit will take up to 9 or 10 hours.

Visit 2 should occur as early as possible in your cycle after menstruation ends. At Visit 2 you will start using the study gel and will insert your first dose in the clinic.

- You will be asked some questions about your health since your last visit, and you will be given a physical exam, if needed.
- Your temperature, blood pressure, and pulse will be taken. You will also give a urine sample to check your health and to see if you are pregnant. If you are pregnant, you will not be able to continue in this study.
- A member of the clinic staff will take 2 tablespoons of blood for lab tests.
- You will have a pelvic exam with a speculum to make sure the biopsies have healed. A swab will be used to take a sample of the fluid from your vagina that will be sent to a laboratory to see how the gel affects your body. If you have any symptoms, additional swabs will be used to take samples from your vagina and cervix for testing. If you have an infection, you will be treated or referred.
- You will be given a plastic applicator that has study gel in it. A study clinician will show you how to insert the gel inside of your vagina. She will observe your first application of the study gel. You will be asked to give the applicator back to study staff after you insert the gel.
- You will have blood drawn 2 times during your visit to see how PC-6500 is affected by the body and how long it can be measured in the blood after dosing. During each blood draw, approximately 2 teaspoons of blood will be taken. The first blood draw will be before you insert your dose of gel. The second blood draw will be 4 hours or 8 hours after you apply the gel, depending on the time you are assigned at enrollment.

- Before and after your first dose of gel, you will have a saline solution squirted into your vagina to collect a specimen that will be sent to the laboratory for testing. These specimens, called “lavages,” will help scientists to learn more about how the gel works in the body. The first lavage will be taken before you insert the gel. The second lavage will be taken 4 or 8 hours after your first dose of gel, depending on the time you are assigned at enrollment (Day 2).
- While you are at the clinic, your vital signs will be taken periodically to see how your body is responding to the gel.
- You will be given meals and snacks while you are at the clinic.
- You may have a physical exam before leaving the clinic, if the clinician thinks it is necessary.
- You will be able to return home after the clinician has checked that you have tolerated your dose of gel.

You will be given applicators filled with gel to take home as well as instructions to remind you how to insert the gel when you are at home. You will be asked to insert one applicator of the gel each day at the same time of day, starting the next day. You will be asked to save each used applicator in an individual plastic bag. On each bag, you will be asked to write the time and date that you inserted the applicator. You will also be instructed on how to save all of the applicators after you use them, and bring them back to the clinic on your next study visit.

Visits 3, 4 and 5 (Days 3, 8 and 11)

Visits 3, 4 and 5 will each take about 2 hours.

- You will be asked some questions about your health since your last visit, and you will be given a physical exam, if needed.
- A member of the clinic staff will take 2 tablespoons of blood for lab tests.
- Your temperature, blood pressure, and pulse will be taken.
- **On Day 8 (Visit 4),** and at other visits if the study staff thinks it is necessary, you will also give a urine sample to check your health and to see if you are pregnant. If you are pregnant, you will not be able to continue in this study.
- **On Day 8 (Visit 4),** and at other visits if the study staff thinks it is necessary, you will have a pelvic exam with a speculum.
- At each visit you will be given an applicator that has study gel in it. You will be asked to insert the gel at the clinic and to give the used applicator back to study staff after you insert it.
- You will also be given applicators to use at home between your clinic visits.

Visit 6 (Day 14)

This visit will last up to 10 hours. This visit will include inserting the **final dose.**

- You will be asked some questions about your health since your last visit, and you will be given a physical exam if needed.
- A member of the clinic staff will take 2 tablespoons of blood for lab tests.
- Your temperature, blood pressure, and pulse will be taken. You will also give a urine sample to check your health and to see if you are pregnant.

- You will be given an applicator that has study gel in it. You will be asked to insert the gel in the clinic and give the used applicator back to study staff after you insert it.
- You will have blood drawn 7 times during your visit to see how PC-6500 is affected by the body and how long it can be detected. To use only one needle stick for all of the blood draws, you may choose to have a small tube (a saline lock) put into a vein in your arm for the first blood draw that will be left in place during your stay in the clinic; it will be removed once all blood draws have been completed. You may also choose to have a needle stick for each blood draw instead of the saline lock. During each blood draw, approximately 2 teaspoons of blood will be taken. Blood draws will happen:

  - Before you apply the gel;
  - 30 minutes after you apply the gel;
  - 1 hour after you apply the gel;
  - 2 hours after you apply the gel;
  - 4 hours after you apply the gel;
  - 6 hours after you apply the gel; and
  - 8 hours after you apply the gel.

- While you are at the clinic, your vital signs will be taken periodically to see how your body is responding to the gel.
- You will be given meals and snacks while you are at the clinic.
- You may have a physical exam before leaving the clinic, if the study clinician thinks it is necessary.

Visit 7 (Day 15)

This visit will last 1-2 hours.

- You will be asked some questions about your health since your last visit, and you will be given a physical exam if needed.
- A member of the clinic staff will take 2 tablespoons of blood for lab tests.
- Your temperature, blood pressure, and pulse will be taken.
- You will have a pelvic exam with a speculum.
  - A swab will be used to take a sample of fluid from your vagina. You will have a lavage to collect a specimen that will be sent to the laboratory for testing. These tests will help scientists understand more about how the gel works in the body.
  - During this exam, 3 biopsies will be taken (2 from your cervix, 1 from your vagina). A gel (benzocaine) can be put on the biopsy areas to numb them. You may take Tylenol® up to one hour before the procedure. Instructions on how to care for your biopsies will be reviewed.

Visit 8 (Day 21)

This visit will last 1-2 hours.

- You will be asked some questions about your health since your last visit, and you will be given a physical exam.
- A member of the clinic staff will take about 2 tablespoons of blood for lab tests.
- Your temperature, weight, blood pressure, and pulse will be taken. You will also give a urine sample to check your health and to see if you are pregnant.

- You will have a pelvic exam with a speculum to check your vagina and cervix for any abnormalities (redness, swelling, etc.) that might have been caused by using the study gel. If you have any symptoms, samples will be taken from your vagina and cervix for testing. If you have an infection, you will be treated or referred for treatment.

Visit 9 (Day 28)

This visit will last less than an hour.

- You will be asked some questions about your health since your last visit.
- You will be given a physical exam, if the study clinician thinks it is necessary.
- A member of the clinic staff will take less than 1 tablespoon of blood for testing related to the study.
- Your temperature, blood pressure, and pulse will be taken. You will also give a urine sample to see if you are pregnant and, if necessary, to see if you have an infection.

Unscheduled Visits:

It may be necessary for you to make additional visits and complete additional procedures. This could happen if any of the study procedures listed above need to be repeated (for example, if there are unexpected abnormal results); difficulties in sample shipping, processing, or testing; and/or if you are experiencing any symptoms or changes in your physical condition.

A description of this clinical trial is available on www.ClinicalTrials.gov, as required by US law. This Web site will include a summary of the results after the study is finished, but will not include information that can identify you. You can search this Web site at any time.

What If I become pregnant during the study?

If you miss a period, or think you might be pregnant during the study, you must tell the study doctor immediately. If you become pregnant, you must stop taking the study drug. The study doctor will ask for your permission to collect information about the outcome of your pregnancy and the condition of your newborn.

Will there be testing for HIV?

Yes, HIV testing will be done during this research study. The following is important information about HIV, HIV testing, and your test results:

- HIV causes AIDS and can be spread through sexual activity, sharing needles, by pregnant women to their fetuses, and through breastfeeding infants.
- There is treatment for HIV that can help you stay healthy.
- People with HIV or AIDS should adopt practices to protect people in their lives from becoming infected with HIV.
- HIV testing is voluntary and can be done anonymously at a public testing center. However, testing is required if you want to be in this research study.
- The law protects the confidentiality of HIV-related test results.
- The law prohibits discrimination based on your HIV status and services are available to address any discrimination.

- If as a result of participation in this study you are INITIALLY diagnosed with HIV, the results must be reported to the New York State Department of Health for contact tracing purposes.
- If as a result of participation in this study you are diagnosed with HIV, you will be given HIV counseling or a referral for HIV counseling.

Genetic Testing

This study will not involve genetic research or genetic testing.

Specimen Banking (Future Use and Storage)

We will store your specimens and information about you in a “biobank,” which is a library of information and specimens (tissue and blood) from many studies. These specimens and information can be linked to you. In the future, researchers can apply for permission to use the specimens and information for new studies to prevent, diagnose or treat disease, including genetic research. If you agree to the future use, some of your de-identified genetic and health information (not linked to you) may be placed into one or more scientific databases. These may include databases maintained by the federal government. Your specimens and information may be kept for a long time, perhaps longer than 50 years. You may remove your consent for future research at any time by contacting the Principal Investigator named on the first page of this consent form or the IRB office at 718-430-2237. If you do, we will destroy any remaining specimens and information but if these were already shared with other researchers, we cannot get them back.

You can choose not to participate in the biobank and still be part of the main study and this will not affect your treatment at this facility**.**

INITIAL ONE (1) OF THE FOLLOWING OPTIONS
_______ I consent to have my specimens and information about me used for future research studies.
_______ I do NOT consent to have my specimens and information about me used for future research studies. The specimens and information will be destroyed at the end of the study.

Information Banking (Future Use and Storage)

We will store information about you in a “bank,” which is a library of information from many studies. This information can be linked to you. In the future, researchers can apply for permission to use the information for new studies to prevent, diagnose or treat disease, including genetic research. If you agree to the future use, some of your de-identified genetic and health information (not linked to you) may be placed into one or more scientific databases. These may include databases maintained by the federal government. Your information may be kept for a long time, perhaps longer than 50 years. You may remove your consent for future research at any time by contacting the Principal Investigator named on the first page of the consent form or the IRB office at 718-430-2237. If you do, we will destroy the information in the bank, but if the information was already shared with other researchers, we cannot get it back.

You can choose not to participate in the bank and still be part of the main study and this will not affect your treatment at this facility**.**

INITIAL ONE (1) OF THE FOLLOWING OPTIONS
_______ I consent to have my information used for future research studies.
_______ I do NOT consent to have my information used for future research studies. The information will be destroyed at the end of the study.

INITIAL YOUR CHOICE BELOW
I consent to be contacted in the future to learn about:
_______ New research protocols that I may wish to join.
_______ General information about research findings.
_______ I do not want to be contacted at all.

Will I be paid for being in this research study?

You will receive a total of $850 if you complete all 10 visits, including screening. You will be compensated $50 for screening, $125 for Visit 1, $75 for Visit 2, $50 for Visit 3, $50 for Visit 4, $50 for Visit 5, $175 for Visit 6, $125 for Visit 7, $50 for Visit 8 and $100 for Visit 9. You will receive your reimbursement through a pre-loaded debit card called a ClinCard, which you must keep for the entire study. If you choose to withdraw from the study before all visits are completed, you will be paid only for the visits you completed. This compensation is for your time, food, and travel related to participating in the study.

Participants in this study may receive more than $600 in a calendar year for their participation. The IRS requires that we report this as income. Therefore, you must provide your social security number if you wish to receive these payments.

Some researchers may develop tests, treatments or products that are worth money. You will not receive payment of any kind for your specimens and information or for any tests, treatments, products or other things of value that may result from the research.

Will it cost me anything to participate in this study?

There will be no cost to you to participate in the study. However, we will not compensate for travel costs, missing work and/or childcare.

Taking part in this study will not involve added costs to you. All study drugs will be given free of charge by the Population Council.

What will happen if I am injured because I took part in this study?

If you are injured as a result of this research, only immediate, essential, short-term medical treatment, as determined by the participating hospital, will be available for the injury without charge to you personally.

- No monetary compensation will be offered.
- You are not waiving any of your legal rights by signing this informed consent document.

- If additional treatment is required as a result of a physical injury related to the research, necessary medical treatment will be provided to you and billed to your insurance company or to you as part of your medical expenses.

Immediately report any discomforts, problems or injuries you experience during the course of your participation in the study to Dr. Marla Keller at (718) 430-3240.

What else do I have to do?

- You must tell the research study doctor about any past and present diseases or allergies you are aware of and about all medications you are taking including "over-the-counter" remedies and nutritional supplements or herbs.
- You must take your study drug as instructed, returning any unused study drug (including any empty applicators), at the scheduled visits.
- If you do not feel well at any time, call your doctor or the research study doctor immediately.
- ***Some drugs may cause a reaction that, if not treated promptly, could be life-threatening. It is important that you report all symptoms, reactions and other complaints to the research study doctor.***
- If you think you have become pregnant, contact your research study doctor immediately.
- If any other doctor recommends that you take any medicine, please inform him/her that you are taking part in a research study. You should give the other doctor the research study doctor's name and phone number.
- You may carry out all your normal daily activities, except vaginal product use and sexual activity, as restricted (abstinence from 96 hours before enrollment through Day 17).

Confidentiality

We will keep your information confidential. Your research records will be kept confidential and your name will not be used in any written or verbal reports. Your information will be given a code number and separated from your name or any other information that could identify you. The form that links your name to the code number will be kept in a secure manner and only the Investigator and study staff will have access to the file. All information will be kept in a secure manner and computer records will be password protected. Your study information and specimens will be kept as long as they are useful for this research.

Medical information collected during the research, such as test results, may be entered into your Montefiore electronic medical record and will be available to clinicians and other staff at Montefiore who provide care to you.

The only people who can see your research records are:

- the research team and staff who work with them
- clinicians and staff at Albert Einstein/Montefiore who review your records for your care and individuals who are authorized to audit or monitor the research
- Population Council study staff and monitors hired by the Population Council.
- groups that review research (the Einstein IRB, Population Council IRB, the Office for Human Research Protections, and the US FDA)

The people who receive your health information may not be required by privacy laws to protect it and may share your information with others without your permission, if permitted by laws governing them. All of these groups have been asked to keep your information confidential.

Are there any risks to me?

GRFT (PC-6500)

It is not expected that this trial will expose you to any unreasonable risk. However, as this is the first time GRFT is being tested in humans, there may be risks that we are unaware of. Some women who have used other vaginal products have experienced genital irritation during or after use. The first 7 women who inserted a single dose of GRFT vaginally experienced no problems. However, since this is the first time GRFT is being used vaginally for 14 days, it is not known whether or not it will cause any irritation or discomfort.

Carrageenan gel (PC-535)

Thousands of women have used carrageenan-containing gels that have been tested in clinical trials around the world. The results of these trials showed that carrageenan gel was safe for vaginal use and was not likely to irritate the vagina. Side effects, which were uncommon and mild, included vaginal discharge and discomfort.

Blood draws

Blood draws may lead to excessive bleeding, discomfort, feelings of dizziness or faintness, and/or bruising, swelling and/or infection. Rarely, the vein where we inserted the needle will become sore or red. Sometimes, a temporary harmless “black and blue” mark may develop. Very rarely, fainting may occur.

Pelvic exams

The pelvic exams during the study may cause you mild discomfort and/or embarrassment. When the study staff takes pelvic specimens, this may cause spotting during or after the pelvic exam. Disclosure of HIV and STI status may cause worry, sadness or depression.

Risks of biopsies

You may have some spotting of blood or bleeding, light drainage consisting of a light amount of blood or clear fluid from the place where tissue is taken after the biopsy procedure is completed. Some patients have cramping and/or pain after the tissue sample is taken. Most women have little or no discomfort (pain) after the tissue sample is taken. These side effects are normally seen after a biopsy. You will receive a small amount of anesthesia or something to numb the area where the tissue will be taken. It is a medicine like Novocain when the dentist gives an injection to treat a dental cavity in your tooth. You will feel numbness in the area where tissue is taken during and after the procedure. There may or may not be some discomfort after the numbness goes away.

All of the above side effects are expected to be mild to moderate. You may not experience any of these side effects or you may experience one or more of them. If you experience any of these side effects and you have them when the study ends, the study staff will follow-up with you until you are better.

Abstinence

If you do not follow the study instructions and engage in sexual intercourse during the study (from 96 hours before enrollment through Day 17), and if you do not use a condom, you may be at risk of getting an STI, including HIV.

Risks to women who are or may become pregnant
The effect of GRFT on an embryo or fetus (developing baby still in the womb), or on a breastfeeding infant, is unknown and may be harmful. Because of these unknown risks, women cannot take part in this study if they are:

- Pregnant
- Trying to become pregnant
- Breastfeeding or sharing breast milk

If you are sexually active and able to become pregnant, you must agree to use one of the birth control methods listed below. You must use birth control for the duration of the study.

Acceptable birth control methods for use in this study are:

- Hormonal methods, such as birth control pills, patches, injections, or implants
- Intrauterine device (IUD)
- Tubal ligation or male partner sterilization
- Same-sex relationship

Birth control methods that are not allowed:

- Barrier methods (such as a condom or diaphragm) used with a spermicide (a foam, cream, or gel that kills sperm)
- NuvaRing®

If you miss a period, or think you might be pregnant during the study, you must tell the study doctor immediately. If you become pregnant, you must stop taking the study drug. The study doctor will ask for your permission to collect information about the outcome of your pregnancy and the condition of your newborn.

Allergic reaction to study drug
Any drug can cause an allergic reaction which could be mild or more serious and can even result in death. Common symptoms of an allergic reaction are rash, itching, skin problems, swelling of the face and throat, or trouble breathing. If you are having trouble breathing, call 911 immediately.

New findings
If we learn any significant new findings during the study that might influence your decision to participate, we will contact you and explain them.

Unknown risks
We have described all the risks we know. However, because this is research, there a possibility that you or an embryo or fetus will have a reaction that we do not know about yet and is not expected. If we learn about other risks, we will let you know what they are so that you can decide whether or not you want to continue to be in the study.

Taking study drug with other medications

The following medications are not allowed during the course of the study:

- Vaginal and rectal products (including, but not limited to vaginal medications, spermicidal products, lubricants, douches)
- Antiretroviral therapy for prevention or treatment
- HSV treatment: Acyclovir, valacyclovir or other medication
- Systemic steroids
- Systemic antibiotics
- Antihypertensive medications, including diuretics
- Investigational therapies for any medical condition

Taking these drugs and PC-6500 together may cause side effects or impact the study in a negative way.

For your safety during this study, call your study doctor BEFORE you take any:

- New medications prescribed by your doctor
- Other medications sold over-the-counter without a prescription
- Dietary or herbal supplements

Are there possible benefits to me?

You will not experience any direct benefit personally from participating in this study. An indirect benefit of taking part in this study is that you are helping researchers gain knowledge and understanding of a new microbicide (drug that may prevent infection from HIV) and that a new microbicide may help others in the future.

What choices do I have other than participating in this study?

You can choose not to participate in the study.

Are there any consequences to me if I decide to stop participating in this study?

No. If you decide to take part, you are free to stop participating at any time without giving a reason. However, some of the information may have already been entered into the study and that will not be removed. The researchers and the sponsor may continue to use and share the information they have already collected.

To revoke (take back) your consent and authorization, you must contact the Principal Investigator in writing at the address on page 1 of this form. However, you may first call or speak to the Principal Investigator and she will stop collecting new information about you. If you take back your consent and authorization, you will not be allowed to continue to participate in this research study.

If you decide to stop taking part in the study for any reason, we will ask you to make a final study visit to make sure you are alright. We will also ask you to return all unused study drug and applicators at this visit.

The final study visit will take about a half hour to an hour.

At this visit, we will:

- Check your blood pressure, temperature, weight, and heart rate
- Perform a pelvic exam and/or physical exam, if needed

Can the study end my participation early?

It is possible that you may be removed from the study without your consent in the event of any of the following:

- If you have an adverse reaction or side effect to GRFT (PC-6500) gel or the placebo (PC-535) gel.
- If you need a treatment that is not allowed in this study.
- If you do not keep appointments.
- If you do not use the gel product that you are assigned as instructed.
- If you are not able to follow study instructions.
- If continuing the study is harmful to you.
- If you become pregnant.
- If the study is canceled or ends early.

Your responsibilities as a research volunteer are as follows:

- To attend each study visit as scheduled.
- To use the study gel as instructed.
- To contact the study clinic if you have any problems or cannot attend a visit.
- To ask any questions when you do not understand something that has been said.
- To tell the study staff, nurse or doctor if you do not feel well or if you have any side effects, even if you do not think they are important.

CONSENT TO PARTICIPATE

I have read the consent form and I understand that it is up to me whether or not I participate. I know enough about the purpose, methods, risks and benefits of the research study to decide that I want to take part in it. I understand that I am not waiving any of my legal rights by signing this informed consent document. I will be given a signed copy of this consent form.

Printed name of participant	Signature of participant	Date	Time
Printed name of the person conducting the consent process	Signature	Date	Time

Appendix 5. DAIDS Grading Criteria

US Department of Health and Human Services, National Institutes of Health, National Institute of Allergy and Infectious Diseases, Division of AIDS. Division of AIDS (DAIDS) Table for Grading the Severity of Adult and Pediatric Adverse Events, Version 2.0. [November 2014]. Available from http://rsc.tech-res.com/clinical-research-sites/safety-reporting/daids-grading-tables. Accessed January 31, 2017.

DAIDS_AE_Grading_Table_v2_NOV2014-1.p

Addendum_1_Female_Genital_Grading_Tab
