# Supplementary material for: Results of a phase 1, randomized, placebo-controlled first-in-human trial of griffithsin formulated in a carrageenan vaginal gel
Source: PLoS One. 2022 Jan 20;17(1):e0261775. doi: 10.1371/journal.pone.0261775 (PMC8775213; doi:10.1371/journal.pone.0261775)
Supplement: S1 Table — (DOCX) [file pone.0261775.s005.docx]

|  |  | Cervix  Squamous epithelium | Cervix Stroma | Vagina  Squamous epithelium | Vagina  Stroma |
| --- | --- | --- | --- | --- | --- |
| Group | Visit | Score | Score | Score | Score |
| PC-6500 | Baseline | 0 | 1 * | 0 | 0 |
|  | Day 15 | 0 | 1 | 0 | 2 |
| PC-6500 | Baseline | 1 | 1 | 1 | 1 |
|  | Day 15 | 0 | 1 | 1 | 1 |
| PC-6500 | Baseline | 1 | 1 | 1 | 1 |
|  | Day 15 | 1 | 2 | 1 | 2 |
| PC-6500 | Baseline | 0.5 | 1.5 + | 0 | 0.5+ |
|  | Day 15 | 0.5 | 0.5 | 1 | 1+ |
| PC-6500 | Baseline | 1.5+ | 2.5+ | 1+ | 1+ |
|  | Day 15 | 1 | 1 | 1 | 1+ |
| PC-6500 | Baseline | 1+ | 1+ | 0 | 1+ |
|  | Day 15 | 1 | 0 | 0 | 1 |
| PC-6500 | Baseline | 0 | no stroma | 1 | 1.5 |
|  | Day 15 | 0.5 | 1.5 | 1+ | 1+ |
| PC-6500 | Baseline | 1 | 1 | 1 | 1 |
|  | Day 15 | 1 | no stroma | 0.5 | 1 |
| PC-6500 | Baseline | 0.5 | 1+ | 1+ | 1+ |
|  | Day 15 | 1 | 1 | 0.5+ | 0.5-1 |
| PC-6500 | Baseline | 1+ | 1+ | 1+ | no stroma |
|  | Day 15 | 0.5-1 | 0.5-1 | 0 | 0.25+ |
| PC-6500 | Baseline | 1 | 1 | 1 | 1 |
| PC-6500 | Baseline | 0.5 | 1+ | 1+ | 1.5 |
| placebo | Baseline | 0 | 1 | 1 | 2 |
|  | Day 15 | 1 | 1 | 0 | 1 |
| placebo | Baseline | 1 | 1 | 1 | 0 |
|  | Day 15 | 1+ | 1+ | 0.5 | 2 |
| placebo | Baseline | 0 | no stroma | 0 | 1 |
|  | Day 15 | 0.5 | 1+ | 1+ | 2+ |

**S1 Table. Histopathology**

Per high-power (400x) field, a score of 0 = no inflammatory cells,  score 0.5 = up to ~12 cells, score of 1.0 = up to ~25 cells, score of 1.5 = up to ~ 37 cells, score of 2 = up to ~ 50 cells, score of 2.5= up to ~ 62 cells, score of 3.0 = > 75 (~100+ inflammatory cells)

* - minimal stroma present
